# Development and internal–external validation of an interpretable machine learning model integrating clinical and polygenic predictors for coronary artery disease: a multicenter retrospective cohort study

**DOI:** 10.3389/fcvm.2026.1936899

**Published:** 2026-09-24

**Authors:** Fangmeng Lei, Xiangwen Xi, Zihe Dang, Shaoqing Cao, Yanqing Shen, Wenjing Hu, Qiang Huang, Weiwei Zhang, Yanwei Yin, Wenlin Lu

**Affiliations:** 1 Department of Cardiology, Wuxi No.2 People's Hospital, Wuxi, China; 2 Department of Cardiology, The Second Affiliated Hospital of Hainan Medical University, Haikou, China; 3 Department of Cardiology, The Affiliated Central Hospital of Jiangnan University, Wuxi, China

**Keywords:** coronary artery disease, explainable artificial intelligence, machine learning, polygenic risk score, risk stratification

## Abstract

**Background:**

Accurate primary-prevention risk stratification for coronary artery disease (CAD) remains challenging. We developed and internally validated a framework integrating clinical variables with a polygenic risk score (PRS).

**Methods:**

This three-center retrospective cohort included 2,067 adults without baseline CAD. The primary target was 5-year CAD cumulative incidence, with non-coronary death treated as a competing event. Clinical, PRS-only, and integrated cause-specific eXtreme Gradient Boosting (XGBoost) models were evaluated using leave-one-center-out internal–external validation. Performance assessment included cumulative/dynamic area under the curve (AUC), inverse-probability-of-censoring-weighted Brier score, calibration, and competing-risk decision-curve analysis. PRS associations were estimated using Fine–Gray regression, and model behavior was examined using SHapley Additive exPlanations (SHAP).

**Results:**

During a median follow-up of 6.8 years, 215 participants experienced the primary endpoint; 163 events occurred within 5 years, and the 5-year cumulative incidence was 8.4%. Each 1-SD increase in the principal component-adjusted PRS was associated with the primary endpoint (adjusted subdistribution hazard ratio, 1.37; 95% CI, 1.21–1.55; *p* < 0.001). Based on pooled out-of-center predictions, the integrated model had a 5-year AUC of 0.779 (95% CI, 0.746–0.812), compared with 0.762 (95% CI, 0.728–0.796) for the clinical model; the difference was 0.017 (95% CI, −0.003–0.038). Its Brier score was 0.071, observed-to-expected ratio was 1.01, and calibration slope was 0.97. The integrated model showed a small net-benefit advantage over the clinical model at thresholds of approximately 8%–15%. SHAP ranked PRS, age, and low-density lipoprotein cholesterol highest and showed a concordant interaction pattern between PRS and low-density lipoprotein cholesterol. Five-year cumulative incidences were 2.7%, 7.2%, and 19.8% in the prespecified low-, intermediate-, and high-risk categories (Gray's test *p* < 0.001).

**Conclusions:**

The PRS was independently associated with incident CAD. Integration with clinical variables yielded a modest, statistically uncertain improvement in discrimination and satisfactory calibration during internal–external validation. External validation is required before clinical implementation.

## 1 Introduction

Coronary artery disease (CAD) remains a major cause of death and disability worldwide and imposes a substantial burden on patients and healthcare systems (1). Primary prevention depends on identifying individuals at increased risk before clinical disease becomes apparent so that modifiable risk factors can be managed appropriately (2). Conventional risk algorithms, including the Framingham Risk Score and the Pooled Cohort Equations, combine demographic and clinical characteristics to estimate future cardiovascular risk. Although these tools have supported population-level prevention, their calibration and clinical classification may vary when they are applied to populations that differ from their original derivation cohorts (3).

A polygenic risk score (PRS) summarizes inherited susceptibility by combining the weighted effects of numerous common genetic variants. Because germline genetic variation is present before conventional risk factors become clinically evident, a PRS may provide information that complements measurements obtained during routine clinical assessment. Studies of CAD have shown that genome-wide PRSs can identify substantial gradients in coronary risk (4). However, the performance and individual-level agreement of published scores are not uniform, and their use requires clear reporting of score selection, variant coverage, allele harmonization, population structure, and ancestry-related transportability (5). These considerations are particularly important when a score developed predominantly from European-ancestry genome-wide association data is evaluated in an East Asian population.

Machine-learning methods can accommodate nonlinear predictor effects and interactions without requiring all functional relationships to be specified in advance. Previous studies using routinely collected clinical data have reported modest improvements in cardiovascular risk discrimination with machine-learning algorithms compared with conventional risk models (6, 7). Nevertheless, improved discrimination alone does not establish the validity or clinical usefulness of a prediction model. Evaluation should also account for calibration, prediction error, censoring, competing events, and variation in performance across clinical settings. For multicenter data, random splitting may overstate transportability because patients from the same institutions can contribute to both model development and validation. Internal–external validation by center provides a more informative assessment of performance across participating institutions.

Interpretability is another consideration when complex prediction models are evaluated for potential clinical use. The SHapley Additive exPlanations (SHAP) framework quantifies the contribution of each predictor to model output and can provide both global feature rankings and participant-level explanations (8). SHAP dependence and interaction values can also describe patterns learned by a fitted model. These patterns reflect model behavior and should not be interpreted independently as evidence of biological interaction or causality. Combining interpretable model outputs with formal regression-based interaction analyses can provide complementary, although still observational, evidence regarding relationships between genetic and clinical predictors.

This three-center retrospective cohort study therefore aimed to develop and internally validate a fixed-horizon prediction framework for incident CAD by integrating routine clinical variables with the published genome-wide polygenic score for CAD (GPS_CAD). The primary prediction target was 5-year CAD cumulative incidence, with non-coronary death treated as a competing event. Clinical, PRS-only, and integrated eXtreme Gradient Boosting (XGBoost) models were evaluated using leave-one-center-out internal–external validation and compared with a cause-specific Cox clinical model. The analysis assessed discrimination, prediction error, calibration, and decision-analytic performance and used SHAP to characterize predictor contributions and the interaction pattern between the PRS and low-density lipoprotein cholesterol (LDL-C) learned by the final model.

## 2 Methods

### 2.1 Study design

This three-center retrospective cohort study was conducted to develop and internally validate an interpretable machine learning model integrating clinical data and a PRS to predict incident CAD. Clinical data and pre-existing genotyping records were obtained from adult patients evaluated at Wuxi No. 2 People's Hospital, The Second Affiliated Hospital of Hainan Medical University, and The Affiliated Central Hospital of Jiangnan University between January 1, 2015, and December 31, 2021. Baseline was defined as the date of the first eligible clinical evaluation at which all inclusion criteria were met. Follow-up extended from baseline until the first occurrence of the primary CAD endpoint, non-coronary death, the last documented clinical contact, or the administrative end of follow-up on December 31, 2024, whichever occurred first. The primary prediction target was the 5-year cumulative incidence of CAD, with the 3-year cumulative incidence evaluated in a sensitivity analysis. Model development and validation were performed using leave-one-center-out internal–external cross-validation, with each participating center serving once as the validation dataset and the other two centers serving as the development dataset. The study protocol was approved by the Institutional Review Board of the coordinating center, Wuxi No. 2 People's Hospital [Approval No. (2023) Y-67], and by the ethics committees of the participating centers. The requirement for written informed consent was waived because the study used de-identified electronic health records and pre-existing biobank data.

### 2.2 Study population

Adults aged ≥18 years with linked electronic health record and genotype data were identified at the three participating centers. Structured records were screened for cardiovascular risk factors and previous CAD, biobank linkage was used to confirm genotype availability, and two clinical investigators independently verified eligibility, resolving disagreements by consensus. Screening and exclusion counts with mutually exclusive reasons were recorded for the participant-flow diagram.

Participants were eligible if they met all of the following criteria: (1) age ≥18 years at baseline; (2) an ascertainable baseline date, available baseline clinical assessment, determinable follow-up time, and genotype data that passed individual-level quality control; (3) no documented history of CAD before baseline, including myocardial infarction, angina pectoris, or coronary revascularization; and (4) at least one cardiovascular risk factor, defined as hypertension (systolic blood pressure ≥130 mmHg, diastolic blood pressure ≥80 mmHg, or current antihypertensive treatment) according to the American College of Cardiology/American Heart Association guidelines (9), type 2 diabetes mellitus (fasting plasma glucose ≥126 mg/dL, hemoglobin A1c ≥ 6.5%, or use of glucose-lowering medication) according to the American Diabetes Association criteria (10), dyslipidemia or current statin treatment according to the American Heart Association/American College of Cardiology guideline (11), or age >50 years.

Exclusion criteria were: (1) genotype data that failed quality control or otherwise precluded PRS calculation; (2) inability to determine the baseline date or follow-up time; or (3) a documented life-limiting condition associated with an expected survival of less than 1 year at baseline, including active advanced malignancy, end-stage kidney disease requiring dialysis, or severe hepatic failure. Follow-up duration and post-baseline CAD status were not eligibility criteria. Participants without a documented CAD endpoint were retained until their last valid clinical contact or the administrative end of follow-up. Missing values in individual clinical predictors did not result in participant exclusion.

### 2.3 Outcomes and data collection

The primary outcome was the first occurrence of a composite CAD endpoint comprising non-fatal myocardial infarction (MI), ischemia-driven coronary revascularization, or coronary death. Potential MI events were identified using International Classification of Diseases, Tenth Revision codes I21.x and I22.x and were adjudicated according to the Fourth Universal Definition of Myocardial Infarction (12). Coronary revascularization included PCI, identified using International Classification of Diseases, Ninth Revision, Clinical Modification procedure codes 00.66, 36.01, 36.02, 36.05–36.07, and 36.09, and CABG, identified using codes 36.10–36.19; every procedure was verified against catheterization or operative records. A revascularization qualified as ischemia-driven when it was performed for an acute coronary syndrome or when an angiographic coronary stenosis of at least 50% was accompanied by ischemic symptoms or objective evidence of myocardial ischemia (13, 14). Coronary death was defined as death attributable to acute MI, ischemic heart disease, or sudden cardiac death with clinical or investigative evidence supporting a coronary cause (13). Deaths without sufficient evidence of a coronary cause were not classified as primary endpoint events.

When more than one endpoint component was recorded on the same calendar day, the event was classified hierarchically as coronary death, non-fatal MI, and coronary revascularization. Components occurring on different dates were classified according to the earliest qualifying event. PCI or CABG performed on the same day as a qualifying MI was therefore classified as MI unless an adjudicated coronary death occurred on that date. The event date was the date of the first qualifying component. Death from any cause other than an adjudicated coronary death before the primary endpoint was defined as non-coronary death and treated as the competing event. Participants without a primary or competing event were censored at the last documented clinical contact or on December 31, 2024, whichever occurred first. The prediction outcome was the individual cumulative incidence of the primary CAD endpoint within 5 years after baseline; the corresponding 3-year cumulative incidence was evaluated in a sensitivity analysis. A separate endpoint sensitivity analysis used a hard coronary composite defined as the first occurrence of non-fatal MI or coronary death. Coronary revascularization without a qualifying MI was not counted as an event in this analysis, and follow-up continued until a hard coronary endpoint, non-coronary death, censoring, or the administrative end of follow-up.

A common electronic algorithm identified potential endpoints from diagnostic and procedure codes, biomarkers, electrocardiographic and imaging findings, angiography and operative reports, discharge summaries, and death records. Two cardiologists independently adjudicated each potential endpoint using a standardized form while blinded to baseline predictors and each other's classifications. Disagreements were resolved by a third blinded senior cardiologist. Complete coding and adjudication rules are provided in Supplementary File S1.

Clinical data were extracted using a standardized data dictionary. The 31 prespecified clinical predictors comprised demographic and physiological measures, lifestyle and family-history variables, cardiovascular and metabolic conditions, laboratory measurements obtained within 7 days of baseline, and baseline medication use. Smoking was coded as current versus non-current. Premature family history was defined as atherosclerotic cardiovascular disease in a first-degree male relative before age 55 years or female relative before age 65 years. Estimated glomerular filtration rate was calculated using the 2021 Chronic Kidney Disease Epidemiology Collaboration creatinine equation (15). These variables represented the principal SCORE2 risk domains and additional routinely available factors (16). Together with the PRS, they comprised the 32 candidate predictors; their complete definitions, units, coding, and sources are provided in Supplementary Table S1.

For the secondary China-PAR comparison, baseline waist circumference and residential information were additionally extracted to classify geographic region and urbanization status. These variables were used only to calculate the China-PAR score and were not included among the 31 candidate clinical predictors. Genomic DNA was extracted from pre-archived peripheral blood mononuclear cell samples and genotyped using the Illumina Infinium Global Screening Array. Genotype quality control was performed using PLINK in accordance with established procedures (17). Samples were excluded for a genotype call rate below 98%, discordance between reported and genetically inferred sex, or heterozygosity outside ±3 SD from the cohort mean. Variants were excluded for a call rate below 98%, minor allele frequency below 1%, or Hardy–Weinberg equilibrium *p* < 1.0 × 10⁻⁶. Within the study cohort, principal component analysis was performed after linkage disequilibrium pruning to identify ancestry outliers and calculate the first 10 genetic principal components (18). These components were used for ancestry adjustment of the PRS as described in Section 2.4 and were not counted among the 32 candidate predictors. Separately, for ancestry visualization, East Asian reference samples from the 1,000 Genomes Project Phase 3 were restricted to the same quality-controlled, linkage-disequilibrium-pruned autosomal variants. Principal-component loadings were estimated in the reference samples, and participants from the three study centers were projected onto these loadings. Genotype imputation, allele harmonization, and PRS calculation are described in Section 2.4.

### 2.4 Construction of polygenic risk score

The coronary artery disease polygenic risk score was calculated using the published GPS_CAD developed by Khera et al., corresponding to PGS Catalog accession PGS000013 (19). The original score was constructed using LDpred with a causal-variant fraction of *ρ* = 0.001 and contained weights for 6,630,150 variants. The published variant weights were applied directly; all successfully harmonized variants that met the genotype and imputation quality criteria were included in the score.

Genotype coordinates were aligned to GRCh37/hg19 before imputation. Following the array-level quality-control procedures described in Section 2.3, genotypes were pre-phased using SHAPEIT4 (20) and imputed using Minimac4 (21) with the East Asian ancestry samples from the 1,000 Genomes Project Phase 3 reference panel (22). Imputed variants were retained when the imputation quality was INFO ≥0.80 and the minor allele frequency was ≥1%. The 98% call-rate threshold described in Section 2.3 was applied to directly genotyped variants before imputation and was not reapplied to imputed dosages.

Variants were harmonized using chromosome, base-pair position, reference allele, and effect allele. Alleles were complemented when strand orientation could be determined unambiguously. Palindromic A/T and C/G variants were retained only when their orientation could be resolved using the reference-panel allele frequency; otherwise, they were excluded. Variants with incompatible alleles, duplicated genomic positions, unresolved strand orientation, or unavailable published weights were not included. Before model development, a fixed cross-center scoring set was defined as the intersection of harmonized variants that met all prespecified quality criteria in each of the three centers, and this identical variant set was used to calculate the raw PRS for every participant. The raw PRS was calculated as PRS=*Σ*(*β*ᵢ × Gᵢ), where *β*ᵢ denotes the published log-odds weight for variant i and Gᵢ denotes the imputed effect-allele dosage ranging from 0 to 2. The numbers of published, successfully imputed, harmonized, and scored variants, together with unweighted and effect-weighted coverage, were recorded overall and by center.

Population-structure adjustment and score standardization were performed separately within each leave-one-center-out validation cycle. Principal-component loadings were estimated from linkage-disequilibrium-pruned variants in the two development centers, and participants from the held-out center were projected onto these loadings. Within the development data, the raw PRS was regressed on PC1–PC10, and the residuals were standardized using the development-set mean and SD. For participants in the held-out center, the raw PRS was adjusted using the regression coefficients estimated in the development data and standardized using the same development-set mean and SD. PC1–PC10 were therefore not entered as separate predictors in the XGBoost models. After completion of internal–external validation, the raw PRS used in the final full-cohort model was regressed on PC1–PC10 in the complete cohort, and the resulting residuals were standardized using their full-cohort mean and SD. For descriptive cumulative incidence analyses, the full-cohort PC-adjusted PRS was categorized as low (<−1 SD), intermediate (−1 to <1 SD), or high (≥1 SD). For descriptive summaries, a separate raw PRS Z-score was calculated using the mean and SD of the entire study cohort; this descriptive score was not used for model training or validation. PRS reporting followed the Polygenic Risk Score Reporting Standards (23). The complete imputation, harmonization, and scoring workflow is provided in Supplementary File S2, and variant coverage is summarized in Supplementary Table S2.

### 2.5 Sample size justification

Sample-size adequacy was assessed before model fitting using the framework proposed by Riley et al. for time-to-event prediction models (24). The calculation included all 31 candidate clinical variables and the PRS before feature selection. Based on their prespecified coding, these variables corresponded to 32 candidate predictor parameters. An anticipated C-statistic of 0.75, a Cox–Snell R^2^ of 0.15, a target global shrinkage factor of 0.90, and a maximum absolute difference of 0.05 between the apparent and adjusted Nagelkerke R^2^ were specified. The primary prediction horizon was 5 years. The calculation used an overall primary-endpoint rate of 0.0176 per person-year and a mean follow-up of 5.90 years, corresponding to a 5-year cumulative incidence of 8.4%. Calculations were performed using pmsampsize with a survival-outcome specification. The minimum sample sizes were 1,756 participants with 183 events to achieve a global shrinkage factor of at least 0.90, 1,183 participants with 123 events to limit the difference between the apparent and adjusted Nagelkerke R^2^ to 0.05, and 1,756 participants with 183 events to estimate the overall 5-year risk with adequate precision. At the latter sample size, the estimated 95% CI for the assumed 5-year risk was 7.2%–9.6%. The largest requirement, 1,756 participants with 183 events, was adopted as the minimum sample size. The available sample of 2,067 participants with 215 primary endpoint events exceeded this requirement.

### 2.6 Model development, validation, and interpretation

#### 2.6.1 Missing data and preprocessing

Missingness was reported overall and by center before imputation (Supplementary Table S3). Predictors with more than 20% missingness in an outer-cycle development dataset were excluded from that cycle. Under a missing-at-random assumption, 20 imputations were generated using predictive mean matching with five donors for continuous variables and logistic regression for binary variables. Estimated glomerular filtration rate was passively recalculated from serum creatinine, age, and sex.

In each leave-one-center-out cycle, imputation models were estimated in the two development centers using the candidate predictors, event type, and cause-specific Nelson–Aalen cumulative hazard terms for CAD and non-coronary death (25). The held-out center contributed neither to estimation of the imputation models nor observed outcome or follow-up information. Out-of-center cumulative incidence predictions were averaged across the 20 imputed datasets. A separate full-cohort imputation was performed after validation for final-model fitting, SHAP analyses, and association analyses.

Continuous-variable processing, PRS adjustment, feature selection, and hyperparameter optimization were confined to the development data, and the resulting parameters were applied unchanged to the held-out center.

#### 2.6.2 Feature selection

The 31 prespecified clinical candidate variables and their coding are provided in Supplementary Table S1. Clinical feature selection was performed using LASSO-penalized cause-specific Cox regression with CAD as the event of interest and non-coronary death (26). The PRS was not entered into the LASSO procedure and was included without selection in the PRS-only and integrated models.

Within each outer-cycle development dataset, the 20 imputed datasets were stacked with an analysis weight of 1/20 assigned to each record. Copies of the same participant were assigned to the same cross-validation fold. Ten-fold cross-validation was performed using cv.glmnet with family = “cox” and alpha = 1. The penalty corresponding to the largest *λ* whose cross-validated partial-likelihood deviance was within one standard error of the minimum was selected according to the *λ*.1se rule. Clinical variables with nonzero coefficients at *λ*.1se were retained. Selection was repeated independently in each outer validation cycle, and center-specific selection results are reported in Supplementary Table S4.

After internal–external validation, the same procedure was repeated using the full study cohort to define the clinical predictors for the final model. Figures 1A,B were generated directly from this final glmnet and cv.glmnet object, respectively. The *λ*.min and *λ*.1se values, their logarithms, cross-validated partial-likelihood deviances, standard errors, and numbers of nonzero coefficients were extracted directly from the fitted object.

**Figure 1:**
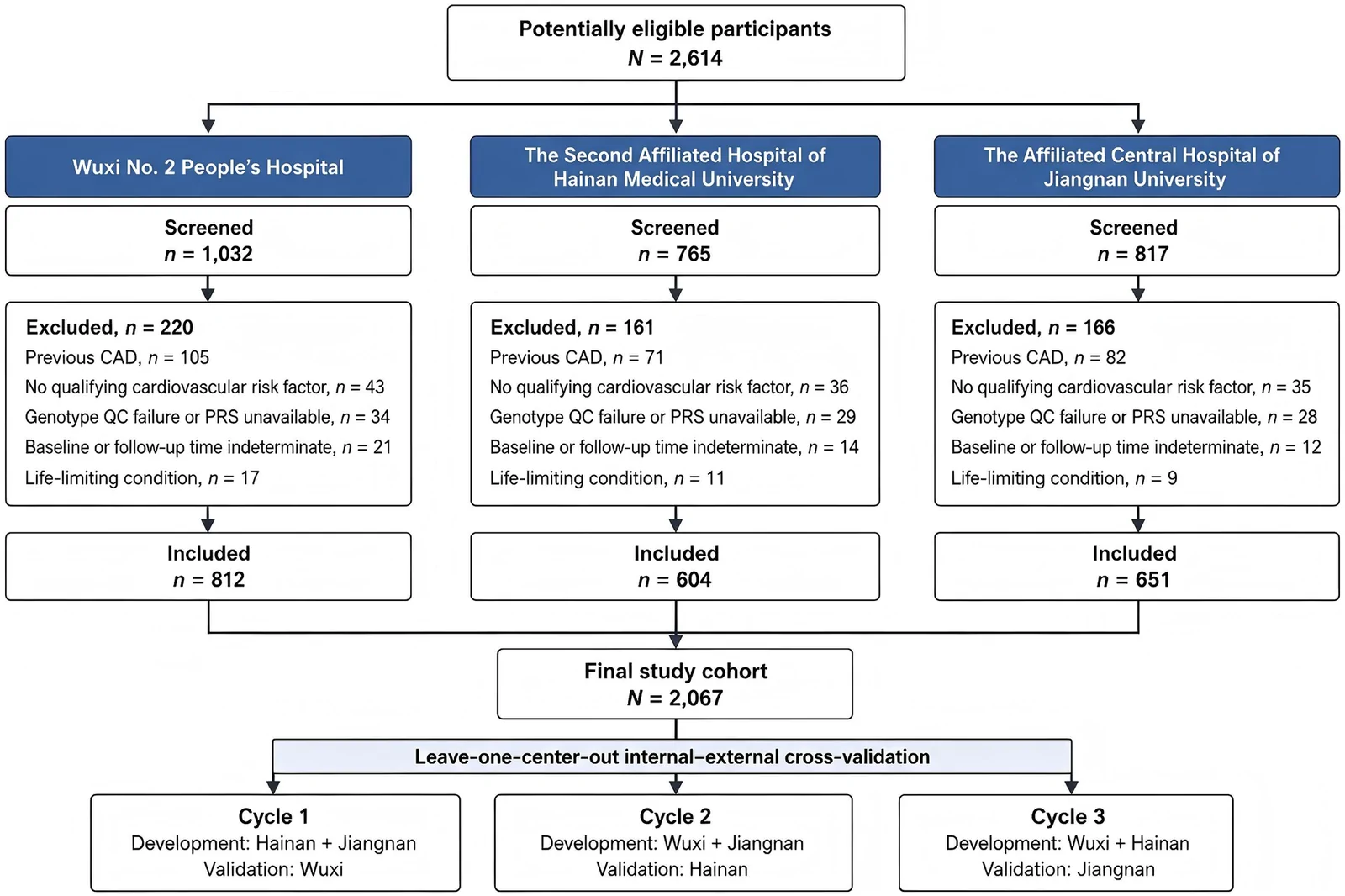
LASSO coefficient paths and cross-validated partial-likelihood deviance in the final full-cohort analysis. **(A)** Coefficient paths for the 31 prespecified clinical candidate variables plotted against log(*λ*). The PRS was not entered into LASSO. **(B)** Mean 10-fold cross-validated partial-likelihood deviance with corresponding SEs. Vertical dashed lines indicate *λ*.min = 0.0118 [log(*λ*) = −4.4397] and *λ*.1se = 0.0236 [log(*λ*) = −3.7465]. At *λ*.min, the deviance was 10.612 (SE, 0.096) and 20 coefficients were nonzero. The upper boundary defined by one SE above the minimum deviance was 10.708. At *λ*.1se, the deviance was 10.701 (SE, 0.099), which was within this boundary, and 15 coefficients were nonzero. LASSO, least absolute shrinkage and selection operator; PRS, polygenic risk score; SE, standard error.

#### 2.6.3 Model development and optimization

Three XGBoost models were developed within the same leave-one-center-out internal–external validation framework: a clinical model containing the LASSO-selected clinical variables, a PRS-only model containing the development-standardized PRS, and an integrated model containing both the selected clinical variables and the PRS (27). Each model was fitted separately in each of the 20 imputed datasets.

XGBoost was fitted using the survival:cox objective. For each predictor set, separate cause-specific models were fitted for the primary CAD endpoint and non-coronary death, with the alternative event type censored at its occurrence. Development-set cause-specific baseline cumulative hazards were estimated using the Breslow method, and the two predicted cause-specific hazards were combined to obtain each participant's cumulative incidence function. The primary model output was the predicted 5-year cumulative incidence of CAD.

Within each outer-cycle development dataset, identical five-fold splits, stratified by center and event type, were used across the 20 imputations, with all copies of a participant assigned to the same fold. For each model, 100 randomly sampled hyperparameter combinations were applied to both cause-specific components. Inner-fold 5-year cumulative incidence predictions were averaged across imputations, and the combination minimizing the mean inverse-probability-of-censoring-weighted Brier score was selected (28). Search ranges were: max_depth, 2–4; eta, 0.01–0.10; min_child_weight, 1–10; subsample and colsample_bytree, 0.70–1.00; gamma, 0–0.50; reg_alpha, 0–1; and reg_lambda, 1–10. Training was limited to 2,000 rounds, with early stopping after 50 rounds without improvement in the same metric.

A cause-specific Cox clinical model containing the same LASSO-selected clinical variables was evaluated as the conventional statistical benchmark. Cause-specific Cox models for CAD and non-coronary death were combined to generate 5-year cumulative incidence predictions using the same procedure applied to XGBoost. The cause-specific Cox models were fitted separately in each of the 20 imputed datasets, and participant-level cumulative incidence predictions were averaged across the corresponding models.

After completion of internal–external validation, the final integrated model was refitted in the 20 full-cohort imputed datasets using the same feature-selection and hyperparameter-optimization procedures. Predictions and SHAP values from these models were averaged for final-model interpretation and were not used to estimate validation performance.

The China-PAR 10-year ASCVD risk equation was evaluated as a secondary established-risk-score comparator among participants aged 35–74 years with all required China-PAR variables available (29). Because China-PAR predicts 10-year ASCVD rather than 5-year CAD, its original risk estimate was used only as a prognostic ranking score for comparison of 5-year time-dependent discrimination. Calibration, Brier score, and decision-curve analysis were not evaluated for China-PAR.

#### 2.6.4 Model performance

Performance was evaluated separately in each held-out center and in the pooled out-of-center predictions. Discrimination was quantified using the 5-year cumulative/dynamic AUC, with non-coronary deaths included among participants without CAD and inverse-probability-of-censoring weighting used for censoring (30). Time-dependent AUCs from 1 to 5 years were additionally plotted to describe changes in discrimination over the prediction interval. Prediction error was assessed using the 5-year Brier score and the integrated Brier score from baseline to 5 years (28).

Calibration was evaluated using the observed-to-expected ratio, defined as the Aalen–Johansen 5-year cumulative incidence divided by the mean predicted risk, and Fine–Gray recalibration intercept and slope, with ideal values of 0 and 1, respectively. Smoothed calibration curves used restricted cubic splines of complementary log-log-transformed predicted risk with knots at the 5th, 35th, 65th, and 95th percentiles (31). Performance estimates and pairwise differences in AUC and Brier score were accompanied by percentile 95% CIs from 1,000 bootstrap samples obtained by resampling participants within centers.

Competing-risk decision curve analysis evaluated net benefit at 5 years over thresholds of 5%–20% (32). Exceeding a threshold represented referral for further cardiovascular risk assessment and intensified risk-factor management. The three XGBoost models and cause-specific Cox benchmark were compared with intervening in all or no participants. Predictions remained continuous, and no classification threshold was used. AUC, Brier score, observed-to-expected ratio, and calibration were repeated at 3 years. The complete integrated-model validation was also repeated for the hard coronary endpoint comprising non-fatal MI or coronary death.

#### 2.6.5 Model interpretation and risk stratification

SHAP values were calculated for the CAD cause-specific component of the final integrated XGBoost model and expressed on the log-relative-hazard scale (33). Values were averaged across the 20 imputed models, and predictors were ranked by mean absolute SHAP value. PRS–LDL-C model behavior was examined using a dependence plot and pairwise SHAP interaction values.

Statistical interaction was evaluated using a Fine–Gray model containing the full-cohort PC-adjusted standardized PRS, LDL-C, and their product term, adjusted for age, sex, body mass index, systolic blood pressure, current smoking, type 2 diabetes, HDL-C, estimated glomerular filtration rate, statin use, center, and PC1–PC10 (34). The PRS was modeled per 1-SD increase, and LDL-C was modeled continuously in mmol/L. Estimates were combined across imputations using Rubin's rules. An interaction pattern was considered supported only when the SHAP pattern and regression coefficient were directionally concordant and the product-term 95% CI excluded zero.

Two individual waterfall plots were generated from the final integrated model. The high-risk representative was the participant whose predicted 5-year risk was closest to the median risk within the highest predicted-risk decile; the low-risk representative was selected analogously from the lowest decile. Each waterfall plot displayed the expected model output and the signed contribution of every included predictor on the log-relative-hazard scale, together with the participant's predicted 5-year cumulative incidence.

Risk stratification was based exclusively on pooled out-of-center 5-year predictions. Participants were categorized as low risk (<5%), intermediate risk (5% to <10%), or high risk (≥10%). Cumulative incidence functions for the primary CAD endpoint were estimated through 5 years using the Aalen–Johansen estimator, with non-coronary death treated as a competing event, and were compared using Gray's test (35). These categories were used to describe risk gradients and were not interpreted as treatment thresholds.

### 2.7 Statistical analysis

All statistical analyses were performed using R version 4.5.1 (R Foundation for Statistical Computing), and machine learning analyses were conducted using Python version 3.11.9. Continuous variables were summarized as means with SDs or medians with interquartile ranges, according to their distributions, and categorical variables were summarized as frequencies and percentages. Baseline characteristics were reported for the complete cohort and separately by study center. Between-center heterogeneity was quantified using the maximum absolute pairwise standardized mean difference for each characteristic; triglycerides, high-sensitivity C-reactive protein, alanine aminotransferase, and aspartate aminotransferase were natural-log transformed for this calculation. Baseline comparisons were not used to determine predictor inclusion. The crude primary-endpoint incidence rate was calculated per 1,000 person-years, with its 95% CI obtained using the exact Poisson method. Median follow-up was estimated using the reverse Kaplan–Meier method. Agreement between the two primary endpoint adjudicators regarding primary-endpoint confirmation before third-cardiologist arbitration was evaluated among all records submitted for adjudication. Overall percentage agreement and Cohen's *κ* were calculated, with the 95% CI for *κ* obtained from 2,000 center-stratified nonparametric bootstrap samples.

Analyses used mice 3.19.0, glmnet 4.1-10, cmprsk 2.2-12, riskRegression 2026.02.13, and pmsampsize 1.1.3 in R; xgboost 2.1.4, scikit-learn 1.6.1, and shap 0.47.2 in Python; and PLINK 1.90b6.21, SHAPEIT4 4.2.2, and Minimac4 4.1.6 for genetic processing. The random seed was 20,260,726.

Fine–Gray subdistribution hazard regression was used to estimate the association between PRS and the primary CAD endpoint, with non-coronary death treated as a competing event. For the continuous and categorical association analyses, the raw PRS was residualized on PC1–PC10 and standardized using the residual mean and SD of the complete cohort. The full-cohort PC-adjusted standardized PRS was entered together with PC1–PC10 and was additionally adjusted for age, sex, body mass index, systolic blood pressure, current smoking, type 2 diabetes mellitus, LDL-C, HDL-C, estimated glomerular filtration rate, baseline statin use, and study center. The same model specification was applied to the hard coronary endpoint comprising non-fatal MI or coronary death. Models were fitted separately in each of the 20 imputed datasets, and coefficients, standard errors, and 95% CIs were combined using Rubin's rules. The proportional subdistribution hazards assumption was assessed using covariate-by-log(time) interaction terms and modified weighted Schoenfeld residuals. Covariates showing a statistically significant time interaction or a systematic residual trend were modeled using time-varying coefficients. All hypothesis tests were two-sided, and *p* < 0.05 was considered statistically significant.

An independent statistician uninvolved in the initial analyses repeated the complete workflow from the frozen dataset and final code, including cohort derivation, endpoint counts, PRS calculation, imputation, model development, internal–external validation, performance estimation, and reproduction of all tables and figures. Reporting followed the TRIPOD + AI statement (36), and the completed checklist is provided in Supplementary File S3. The complete analysis code and reproducibility documentation are provided in Supplementary File S4.

## 3 Results

### 3.1 Study cohort and baseline characteristics

Of 2,614 potentially eligible participants, 547 were excluded and 2,067 were included, comprising 812 participants from Wuxi, 604 from Hainan, and 651 from Jiangnan. Center-specific screening and exclusion counts are shown in Figure 2.

**Figure 2:**
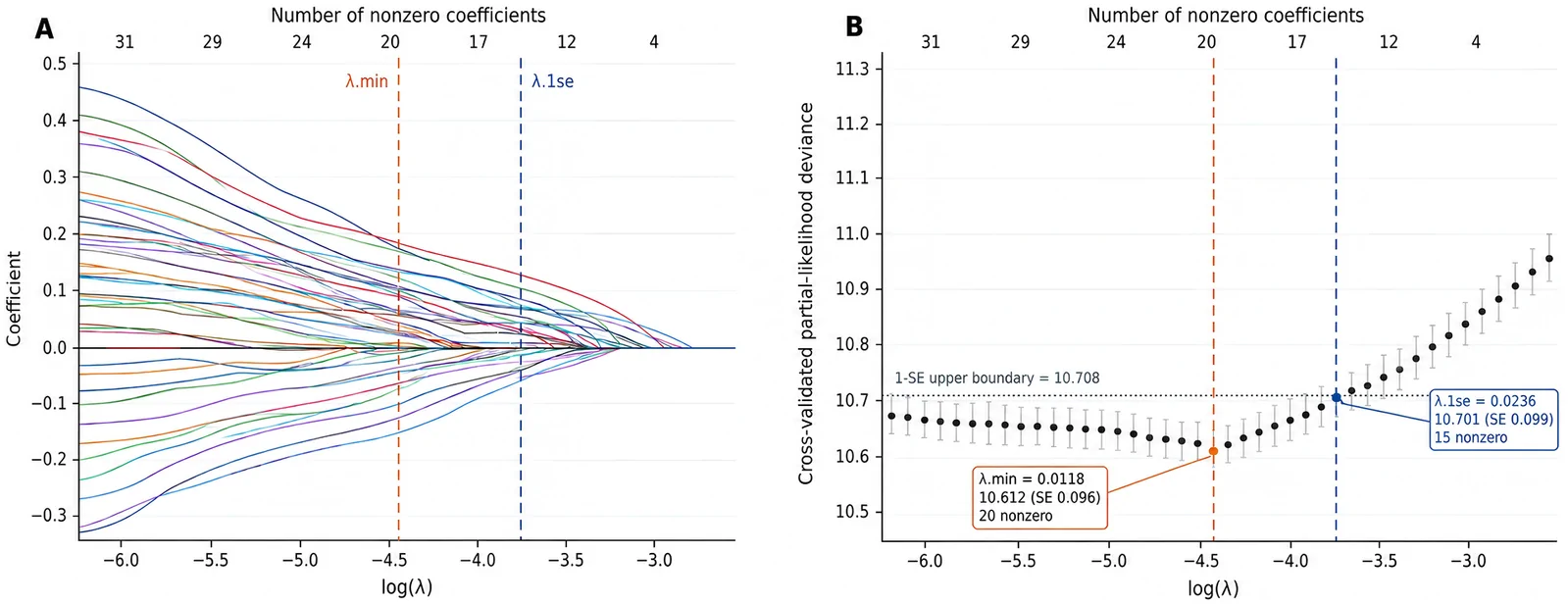
Participant flow through screening, eligibility assessment, and analysis. Among 2,614 potentially eligible participants, 1,032 were screened at Wuxi No. 2 People's Hospital, 765 at The Second Affiliated Hospital of Hainan Medical University, and 817 at The Affiliated Central Hospital of Jiangnan University. At Wuxi, 220 participants were excluded because of previous CAD (*n* = 105), absence of a qualifying cardiovascular risk factor (*n* = 43), genotype data that failed quality control or precluded PRS calculation (*n* = 34), inability to determine baseline or follow-up time (*n* = 21), or a life-limiting condition (*n* = 17). The corresponding exclusion counts were 71, 36, 29, 14, and 11 at Hainan and 82, 35, 28, 12, and 9 at Jiangnan. The final cohort included 812 participants from Wuxi, 604 from Hainan, and 651 from Jiangnan. In leave-one-center-out internal–external cross-validation, each center served once as the validation dataset while the other two centers formed the development dataset. CAD, coronary artery disease; PRS, polygenic risk score.

During 12,216 person-years of observation, 215 participants experienced the primary endpoint and 78 experienced non-coronary death. Within 5 years, 163 primary endpoints and 57 non-coronary deaths occurred; 487 participants were censored before 5 years, and 1,360 remained event-free and under observation at 5 years. The crude incidence rate was 17.6 per 1,000 person-years (95% CI, 15.3–20.1), the median follow-up was 6.8 years (95% CI, 6.6–7.0), and the 5-year cumulative incidence was 8.4% (95% CI, 7.2%–9.6%).

Baseline characteristics are presented in Table 1. The maximum absolute pairwise SMDs ranged from 0.06 to 0.24, with the largest difference observed for resting heart rate. The descriptive raw PRS Z-score was 0.00 ± 1.00 in the complete cohort. Predictor definitions and missingness are reported in Supplementary Tables S1, S3, respectively.

**Table 1:** Baseline characteristics of the study cohort overall and by participating center.

Characteristic	Overall (*N* = 2,067)	Wuxi (*n* = 812)	Hainan (*n* = 604)	Jiangnan (*n* = 651)	Maximum absolute SMD
Demographic and physiological variables					
Age, years	58.8 ± 8.7	59.4 ± 8.6	57.6 ± 9.1	59.1 ± 8.4	0.20
Male sex	1,021 (49.4)	420 (51.7)	277 (45.9)	324 (49.8)	0.12
Body mass index, kg/m²	26.2 ± 3.6	26.5 ± 3.6	25.8 ± 3.7	26.2 ± 3.5	0.19
Systolic blood pressure, mmHg	135.1 ± 14.5	136.4 ± 14.6	133.1 ± 14.2	135.2 ± 14.5	0.23
Diastolic blood pressure, mmHg	82.2 ± 9.6	82.8 ± 9.7	81.5 ± 9.5	82.1 ± 9.6	0.14
Resting heart rate, beats/min	73.6 ± 10.4	73.1 ± 10.2	75.3 ± 10.7	72.8 ± 10.1	0.24
Lifestyle and medical-history variables					
Current smoking	410 (21.0)	171 (22.2)	109 (19.2)	130 (21.1)	0.07
Current alcohol use	353 (18.3)	151 (19.9)	92 (16.3)	110 (18.0)	0.09
Family history of premature ASCVD	338 (18.7)	143 (20.1)	86 (16.4)	109 (19.2)	0.10
Hypertension	1,164 (56.3)	473 (58.3)	322 (53.3)	369 (56.7)	0.10
Type 2 diabetes mellitus	498 (24.1)	206 (25.4)	136 (22.5)	156 (24.0)	0.07
Dyslipidemia	1,126 (54.5)	455 (56.0)	315 (52.2)	356 (54.7)	0.08
Chronic kidney disease	208 (10.1)	88 (10.8)	54 (8.9)	66 (10.1)	0.06
Laboratory variables					
Total cholesterol, mmol/L	4.86 ± 1.10	4.92 ± 1.08	4.78 ± 1.12	4.87 ± 1.09	0.13
LDL-C, mmol/L	3.01 ± 0.89	3.06 ± 0.89	2.94 ± 0.90	3.01 ± 0.87	0.13
HDL-C, mmol/L	1.34 ± 0.35	1.32 ± 0.35	1.36 ± 0.36	1.34 ± 0.34	0.11
Triglycerides, mmol/L	1.57 [1.11–2.15]	1.60 [1.14–2.18]	1.52 [1.08–2.10]	1.57 [1.11–2.16]	0.11
Fasting plasma glucose, mmol/L	5.88 ± 1.29	5.94 ± 1.31	5.80 ± 1.25	5.88 ± 1.29	0.11
Hemoglobin A1c, %	5.99 ± 0.71	6.02 ± 0.72	5.94 ± 0.69	5.99 ± 0.71	0.11
Serum creatinine, μmol/L	78.4 ± 16.4	79.6 ± 16.8	76.8 ± 15.9	78.4 ± 16.3	0.17
eGFR, mL/min/1.73 m²	84.6 ± 15.7	83.6 ± 15.9	86.2 ± 15.4	84.5 ± 15.7	0.17
Uric acid, μmol/L	342.4 ± 84.2	346.0 ± 86.0	338.0 ± 82.0	342.0 ± 84.0	0.10
High-sensitivity C-reactive protein, mg/L	1.6 [0.8–3.3]	1.7 [0.8–3.4]	1.5 [0.7–3.1]	1.6 [0.8–3.3]	0.10
White blood cell count, ×10⁹/L	6.41 ± 1.57	6.50 ± 1.60	6.30 ± 1.50	6.40 ± 1.60	0.13
Hemoglobin, g/L	138.2 ± 15.3	139.1 ± 15.4	136.8 ± 15.1	138.4 ± 15.2	0.15
Platelet count, ×10⁹/L	227.7 ± 56.3	225.0 ± 56.0	231.0 ± 58.0	228.0 ± 55.0	0.11
Alanine aminotransferase, U/L	23 [16–33]	23 [16–34]	22 [15–32]	23 [16–33]	0.08
Aspartate aminotransferase, U/L	22 [18–29]	22 [18–29]	21 [17–28]	22 [18–29]	0.08
Baseline medication use					
Statin use	694 (33.6)	294 (36.2)	182 (30.1)	218 (33.5)	0.13
Antiplatelet use	431 (20.9)	180 (22.2)	115 (19.0)	136 (20.9)	0.08
Antihypertensive drug use	1,006 (48.7)	410 (50.5)	278 (46.0)	318 (48.8)	0.09
Genetic predictor					
Descriptive raw PRS Z-score	0.00 ± 1.00	0.08 ± 0.98	−0.12 ± 1.03	0.01 ± 0.99	0.20

Values are presented as mean ± SD, median [IQR], or n (%). Baseline summaries were calculated from observed values before multiple imputation. Continuous-variable summaries were based on available observations, and percentages for categorical variables were calculated among participants with nonmissing data. Variable-specific missingness overall and by center is reported in Supplementary Table S3. The maximum absolute SMD is the largest absolute standardized mean difference among the three pairwise center comparisons. Triglycerides, high-sensitivity C-reactive protein, alanine aminotransferase, and aspartate aminotransferase were log-transformed for SMD calculation. The descriptive raw PRS Z-score was standardized using the mean and SD of the complete cohort and was not used for model development or internal–external validation. Validation-cycle PRS values were adjusted for PC1–PC10 and standardized exclusively using parameters estimated from the corresponding development datasets. ASCVD, atherosclerotic cardiovascular disease; eGFR, estimated glomerular filtration rate; HDL-C, high-density lipoprotein cholesterol; IQR, interquartile range; LDL-C, low-density lipoprotein cholesterol; PRS, polygenic risk score; SD, standard deviation; SMD, standardized mean difference.

### 3.2 Polygenic risk score construction and distribution

Of the 6,630,150 variants in the published GPS_CAD scoring file, 6,124,685 were available after imputation across all three centers and 4,982,614 remained after quality filtering and allele harmonization. The final common scoring set provided unweighted and effect-weighted coverage of 75.2% and 82.8%, respectively, and was applied consistently across centers (Supplementary Table S2).

The out-of-center PC-adjusted PRS distributions were 0.09 ± 1.02 in Wuxi, −0.13 ± 0.97 in Hainan, and 0.04 ± 1.01 in Jiangnan, with a pooled distribution of 0.01 ± 1.01 (Supplementary Figure S1). For final-model fitting, the full-cohort PC-adjusted PRS was standardized to 0.00 ± 1.00; the corresponding center-specific distributions were 0.06 ± 1.01, −0.09 ± 0.98, and 0.01 ± 1.00. Genetic PC distributions largely overlapped the East Asian reference distribution, with modest center-specific variation (Supplementary Figure S2).

### 3.3 Clinical feature selection and model specification

Clinical feature selection was performed separately in each outer-cycle development dataset using the 31 clinical candidate variables, with the PRS excluded from LASSO. When Wuxi, Hainan, and Jiangnan were held out, the *λ*.1se values were 0.0247, 0.0219, and 0.0263, respectively; the corresponding cross-validated partial-likelihood deviances were 10.842 (SE, 0.118), 10.714 (SE, 0.126), and 10.903 (SE, 0.121), retaining 16, 16, and 15 clinical variables. Twelve variables were selected in all three cycles, three in two cycles, five in one cycle, and 11 in none (Supplementary Table S4).

In the final full-cohort analysis, *λ*.min was 0.0118 [log(*λ*) = −4.4397], with a deviance of 10.612 (SE, 0.096) and 20 nonzero coefficients. At *λ*.1se = 0.0236 [log(*λ*) = −3.7465], the deviance was 10.701 (SE, 0.099), and 15 clinical variables were retained: age, sex, body mass index, systolic blood pressure, current smoking, family history of premature ASCVD, hypertension, type 2 diabetes mellitus, LDL-C, HDL-C, triglycerides, hemoglobin A1c, high-sensitivity C-reactive protein, eGFR, and statin use (Figure 1). The PRS was included without selection in the PRS-only and integrated models.

Across predictor sets and validation cycles, selected tree depths ranged from 2 to 3, learning rates from 0.033 to 0.081, and boosting rounds from 228 to 754. Complete hyperparameter specifications are provided in Supplementary Table S5.

### 3.4 Endpoint incidence and association of PRS with CAD

The 215 primary endpoints comprised 103 non-fatal MIs, 85 ischemia-driven coronary revascularizations, and 27 coronary deaths according to the prespecified hierarchy. Center-specific endpoint counts and cumulative incidences are reported in Supplementary Table S6.

Among 300 records submitted for adjudication, the two primary cardiologists agreed on 276 classifications (92.0%; Cohen *κ*, 0.80; 95% CI, 0.71–0.87) before third-cardiologist arbitration of the remaining 24 records.

Each 1-SD increase in the full-cohort PC-adjusted standardized PRS was associated with the primary endpoint (adjusted sHR, 1.37; 95% CI, 1.21–1.55; *p* < 0.001; Table 2). The low-, intermediate-, and high-PRS groups included 330, 1,403, and 334 participants, with 5-year cumulative incidences of 2.9%, 7.7%, and 17.0%, respectively. Relative to the low-PRS group, the adjusted sHRs were 1.72 (95% CI, 1.04–2.85; *p* = 0.035) for the intermediate group and 3.19 (95% CI, 1.86–5.48; *p* < 0.001) for the high group. The cumulative incidence functions differed across categories (Gray's test *p* < 0.001; Figure 3).

**Table 2:** Association of the polygenic risk score with the primary and hard coronary endpoints.

Endpoint and PRS specification	Participants, n	Events over complete follow-up, n	5-year cumulative incidence, % (95% CI)	Adjusted sHR (95% CI)	*p* value
Primary CAD endpoint					
PRS per 1-SD increase	2,067	215	8.4 (7.2–9.6)	1.37 (1.21–1.55)	<0.001
Low PRS, <−1 SD	330	16	2.9 (1.6–4.9)	1.00 (Reference)	—
Intermediate PRS, −1 to <1 SD	1,403	132	7.7 (6.4–9.2)	1.72 (1.04–2.85)	0.035
High PRS, ≥1 SD	334	67	17.0 (13.2–21.2)	3.19 (1.86–5.48)	<0.001
Hard coronary endpoint					
PRS per 1-SD increase	2,067	141	5.4 (4.4–6.5)	1.42 (1.21–1.67)	<0.001
Low PRS, <−1 SD	330	10	1.8 (0.8–3.5)	1.00 (Reference)	—
Intermediate PRS, −1 to <1 SD	1,403	83	4.7 (3.7–5.9)	1.67 (0.85–3.28)	0.139
High PRS, ≥1 SD	334	48	11.8 (8.5–15.7)	3.37 (1.65–6.89)	0.001

Event counts include all observed events over complete follow-up, whereas cumulative incidences were estimated at the prespecified 5-year horizon using the Aalen–Johansen estimator with non-coronary death treated as a competing event. The primary endpoint comprised the first occurrence of non-fatal MI, ischemia-driven coronary revascularization, or coronary death. The hard coronary endpoint comprised non-fatal MI or coronary death; isolated coronary revascularization was not counted as an event, and follow-up continued after such procedures until a hard coronary endpoint, non-coronary death, or censoring. All continuous and categorical PRS specifications were based on the full-cohort PC-adjusted standardized PRS. Continuous and categorical associations were estimated in separate Fine–Gray models. Models were adjusted for age, sex, body mass index, systolic blood pressure, current smoking, type 2 diabetes mellitus, LDL-C, HDL-C, eGFR, baseline statin use, study center, and PC1–PC10. Estimates were combined across the 20 imputed datasets using Rubin's rules. CAD, coronary artery disease; CI, confidence interval; eGFR, estimated glomerular filtration rate; MI, myocardial infarction; PC, principal component; PRS, polygenic risk score; SD, standard deviation; sHR, subdistribution hazard ratio.

**Figure 3:**
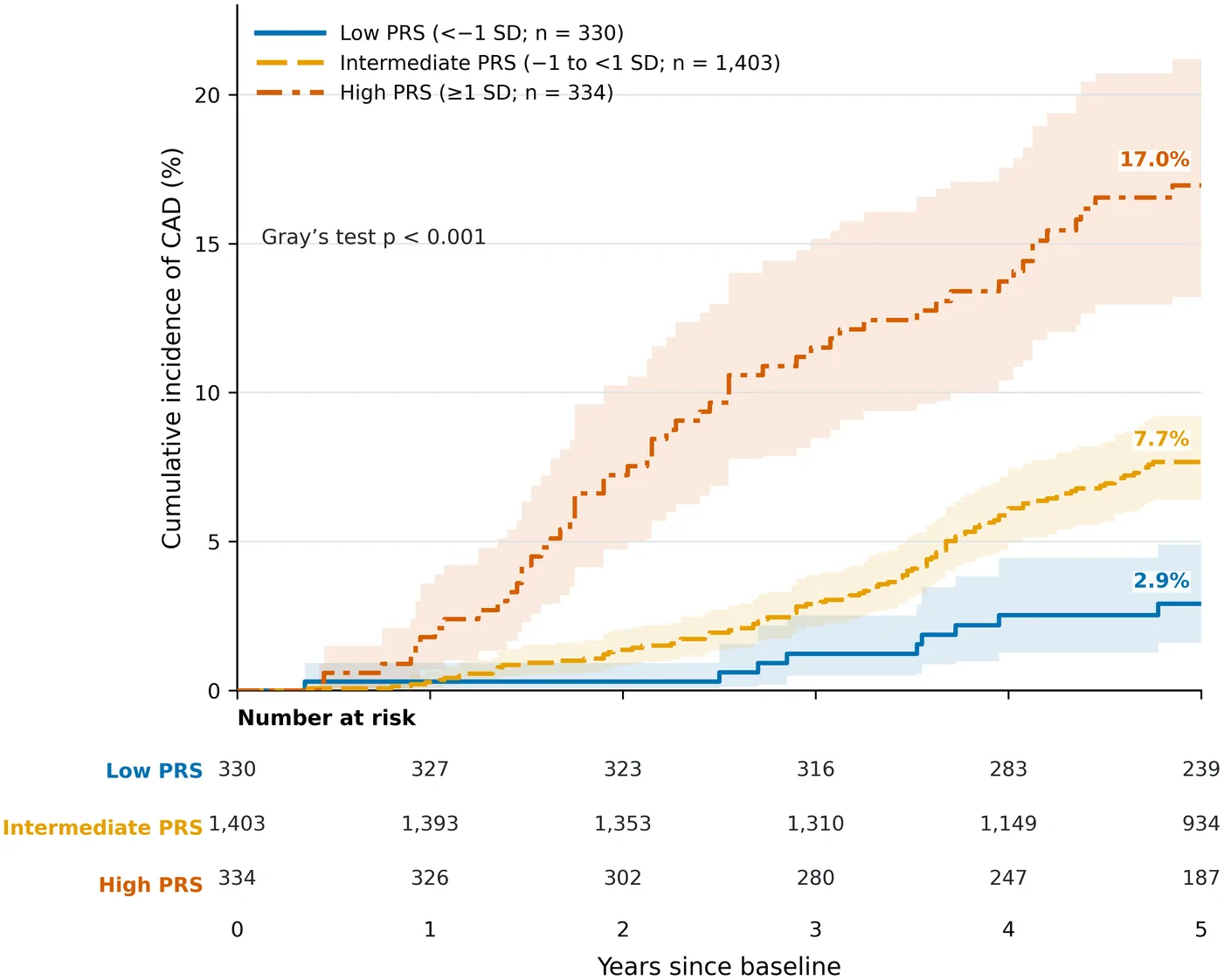
Five-year cumulative incidence of CAD according to fixed standardized polygenic risk score categories. Aalen–Johansen cumulative incidence functions are shown for participants with low (<−1 SD; *n* = 330), intermediate (−1 to <1 SD; *n* = 1,403), and high (≥1 SD; *n* = 334) full-cohort PC-adjusted PRS values, with non-coronary death treated as a competing event. Shaded areas indicate 95% CIs. The cumulative incidence functions differed across the three categories (Gray's test *p* < 0.001). Numbers at risk are displayed at yearly intervals from baseline through 5 years. CI, confidence interval; PRS, polygenic risk score; SD, standard deviation.

Among 141 hard coronary endpoints, each 1-SD increase in the PRS was associated with an adjusted sHR of 1.42 (95% CI, 1.21–1.67; *p* < 0.001). The corresponding sHRs for the intermediate- and high-PRS groups were 1.67 (95% CI, 0.85–3.28; *p* = 0.139) and 3.37 (95% CI, 1.65–6.89; *p* = 0.001), respectively (Table 2). No time-varying PRS association was detected in the primary- or hard-endpoint models (*p* = 0.46 and *p* = 0.52, respectively), and the tests for the PRS-category indicators were also non-significant (all *p* ≥ 0.21). Residual assessment showed no systematic temporal trends, and time-varying coefficients were not required.

### 3.5 Model discrimination, calibration, and transportability

Performance was evaluated using pooled out-of-center predictions for all 2,067 participants, including 163 primary endpoints within 5 years. The integrated model achieved a 5-year AUC of 0.779 (95% CI, 0.746–0.812), compared with 0.762 for the clinical model, 0.681 for the PRS-only model, and 0.748 for the cause-specific Cox model (Table 3). The corresponding *Δ*AUCs were 0.017 (95% CI, −0.003–0.038), 0.098 (95% CI, 0.061–0.135), and 0.031 (95% CI, 0.008–0.055), respectively. Time-dependent AUCs are shown in Figure 4A.

**Table 3:** Five-year performance of the prediction models based on leave-one-center-out internal–external validation.

Model	5-year AUC (95% CI)	Integrated model−comparator *Δ*AUC (95% CI)	5-year IPCW Brier score (95% CI)	Integrated model−comparator *Δ*Brier score (95% CI)	Integrated Brier score, 0–5 years (95% CI)	O/E ratio (95% CI)	Calibration intercept (95% CI)	Calibration slope (95% CI)
Clinical XGBoost	0.762 (0.728–0.796)	0.017 (−0.003 to 0.038)	0.073 (0.065–0.081)	−0.002 (−0.005 to 0.001)	0.055 (0.049–0.061)	1.03 (0.90–1.17)	0.03 (−0.12 to 0.18)	0.94 (0.78–1.10)
PRS-only XGBoost	0.681 (0.640–0.722)	0.098 (0.061–0.135)	0.080 (0.072–0.088)	−0.009 (−0.013 to −0.005)	0.062 (0.056–0.068)	1.07 (0.93–1.23)	0.07 (−0.10 to 0.24)	0.81 (0.65–0.98)
Integrated XGBoost	0.779 (0.746–0.812)	Reference	0.071 (0.063–0.079)	Reference	0.053 (0.047–0.059)	1.01 (0.88–1.15)	0.01 (−0.14 to 0.16)	0.97 (0.81–1.13)
Cause-specific Cox clinical	0.748 (0.712–0.784)	0.031 (0.008–0.055)	0.074 (0.066–0.082)	−0.003 (−0.006 to 0.001)	0.057 (0.051–0.063)	1.05 (0.91–1.20)	0.05 (−0.11 to 0.21)	0.91 (0.74–1.08)

Estimates were calculated from pooled out-of-center predictions for all 2,067 participants. The pooled validation dataset included 163 primary CAD endpoints occurring within 5 years. AUC denotes the 5-year cumulative/dynamic AUC. The 5-year Brier score and integrated Brier score incorporated IPCW. *Δ*AUC and *Δ*Brier were calculated as the integrated-model estimate minus the corresponding comparator estimate; higher *Δ*AUC and lower *Δ*Brier favor the integrated model. Percentile 95% CIs for performance measures and paired differences were obtained from 1,000 bootstrap samples generated by resampling participants within centers. Ideal values are 1 for the O/E ratio, 0 for the calibration intercept, and 1 for the calibration slope. AUC, area under the curve; CI, confidence interval; IPCW, inverse probability of censoring weighting; O/E, observed-to-expected; PRS, polygenic risk score.

**Figure 4:**
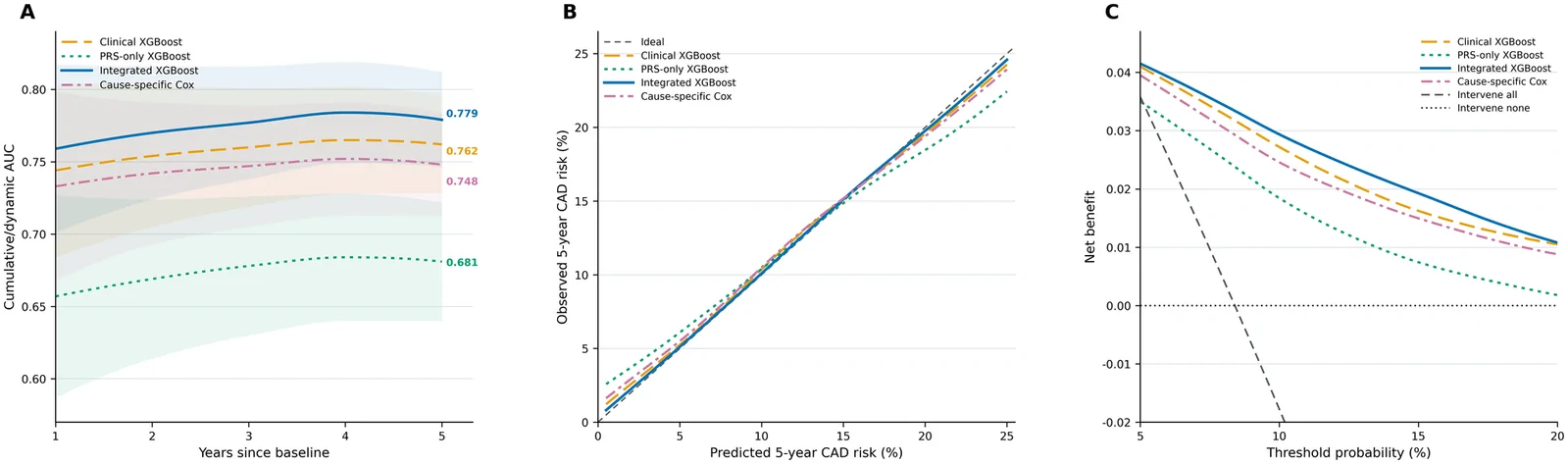
Discrimination, calibration, and clinical utility of the prediction models based on leave-one-center-out internal–external validation. **(A)** Time-dependent cumulative/dynamic AUCs from 1 to 5 years for the clinical XGBoost, PRS-only XGBoost, integrated XGBoost, and cause-specific Cox clinical models. Lines represent estimates from pooled out-of-center predictions, and shaded areas represent 95% CIs obtained from 1,000 center-stratified bootstrap samples. **(B)** Smoothed calibration curves comparing predicted and observed 5-year CAD cumulative incidence. Observed risks were estimated using the Aalen–Johansen estimator with non-coronary death treated as a competing event. The diagonal line indicates ideal calibration. **(C)** Competing-risk decision curves at the 5-year prediction horizon over threshold probabilities from 5% to 20%. Exceeding a threshold represents referral for further cardiovascular risk assessment and intensified management of modifiable risk factors. The models are compared with strategies of intervening in all participants or in none. AUC, area under the curve; CAD, coronary artery disease; CI, confidence interval; PRS, polygenic risk score.

The integrated model had a 5-year Brier score of 0.071 (95% CI, 0.063–0.079) and an integrated Brier score of 0.053 (95% CI, 0.047–0.059). Its 5-year Brier score differed from the clinical, PRS-only, and cause-specific Cox models by −0.002, −0.009, and −0.003, respectively, with the corresponding 95% CIs reported in Table 3. The O/E ratio was 1.01 (95% CI, 0.88–1.15), the calibration intercept was 0.01 (95% CI, −0.14–0.16), and the calibration slope was 0.97 (95% CI, 0.81–1.13; Figure 4B). Across held-out centers, the integrated-model AUC ranged from 0.751 to 0.798 and the Brier score from 0.066 to 0.075 (Supplementary Table S7 and Supplementary Figure S3).

In the competing-risk DCA, the integrated, clinical, and cause-specific Cox models had positive net benefit relative to intervening in no participants across most thresholds from 5% to 20%. The integrated model showed a small numerical advantage over the clinical model at thresholds of approximately 8%–15%, while their curves were similar near the limits of this range (Figure 4C).

The China-PAR comparison included 1,521 participants, with 118 primary endpoints within 5 years. The 5-year AUC was 0.724 (95% CI, 0.681–0.767) for China-PAR and 0.771 (95% CI, 0.731–0.811) for the integrated model, yielding a paired difference of 0.047 (95% CI, 0.013–0.082; Supplementary Table S8).

### 3.6 Model interpretation and fixed-threshold risk stratification

PRS, age, and LDL-C were the three highest-ranked predictors by mean absolute SHAP value, with values of 0.287, 0.264, and 0.231, respectively (Figure 5A). Higher PRS, age, LDL-C, systolic blood pressure, and hemoglobin A1c generally produced positive SHAP contributions, whereas higher HDL-C and eGFR produced negative contributions.

**Figure 5:**
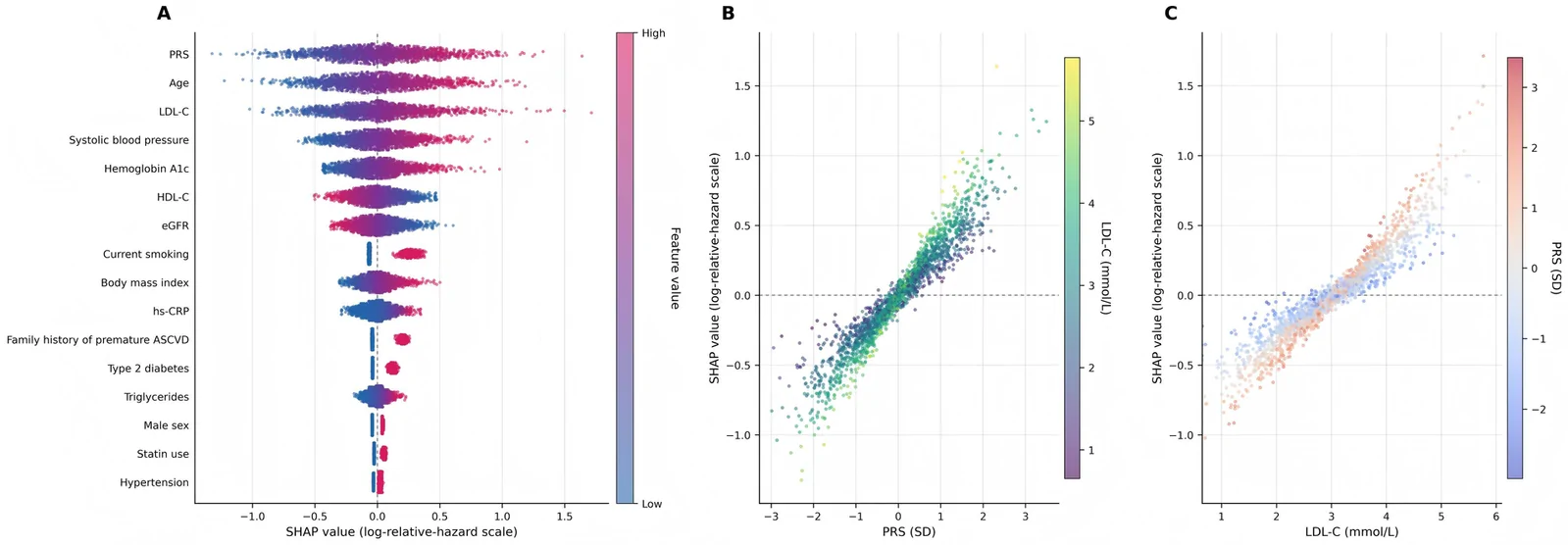
SHAP summary and PRS–LDL-C dependence plots for the integrated model. **(A)** SHAP summary plot for the final full-cohort integrated model, with predictors ordered by mean absolute SHAP value. Each point represents one participant, the horizontal position indicates the predictor's SHAP contribution on the log-relative-hazard scale, and color represents the observed predictor value. PRS, age, and LDL-C were the three highest-ranked predictors in the final model. **(B)** PRS dependence plot showing PRS values on the horizontal axis and corresponding SHAP contributions on the vertical axis, with points colored by LDL-C. **(C)** LDL-C dependence plot showing LDL-C values and corresponding SHAP contributions, with points colored by PRS. The mean absolute PRS–LDL-C SHAP interaction value was 0.041. SHAP values and interaction values were averaged across the 20 full-cohort imputed models. ASCVD, atherosclerotic cardiovascular disease; eGFR, estimated glomerular filtration rate; hs-CRP, high-sensitivity C-reactive protein; LDL-C, low-density lipoprotein cholesterol; PRS, polygenic risk score; SHAP, SHapley additive exPlanations.

PRS-related SHAP contributions were greater at higher LDL-C values (Figures 5B,C), and the mean absolute PRS–LDL-C SHAP interaction value was 0.041. In the Fine–Gray interaction model, the product-term coefficient was *β* = 0.113, with exp(*β*) = 1.12 (95% CI, 1.03–1.22; *p* = 0.009). Thus, each 1 mmol/L higher LDL-C multiplied the sHR associated with a 1-SD higher PRS by 1.12. This direction was concordant with the SHAP pattern, and no time-varying effect was detected for the product term (*p* = 0.41).

The representative participants selected from the lowest and highest predicted-risk deciles had predicted 5-year risks of 1.9% and 25.6%, respectively. The low-risk representative was a 52-year-old woman with a PRS of −1.38 SD and LDL-C of 2.18 mmol/L, whereas the high-risk representative was a 68-year-old man with a PRS of 1.72 SD and LDL-C of 4.12 mmol/L. Their complete predictor contributions are shown in Supplementary Figure S4.

Based on pooled out-of-center predictions, 812 participants were classified as low risk, 771 as intermediate risk, and 484 as high risk. Over complete follow-up, these groups experienced 29, 74, and 112 primary endpoints and 25, 30, and 23 competing deaths, respectively. Their 5-year cumulative incidences were 2.7% (95% CI, 1.7%–4.0%), 7.2% (95% CI, 5.7%–8.9%), and 19.8% (95% CI, 16.2%–23.7%), respectively (Gray's test *p* < 0.001; Figure 6). These categories describe risk gradients and were not evaluated as treatment thresholds.

**Figure 6:**
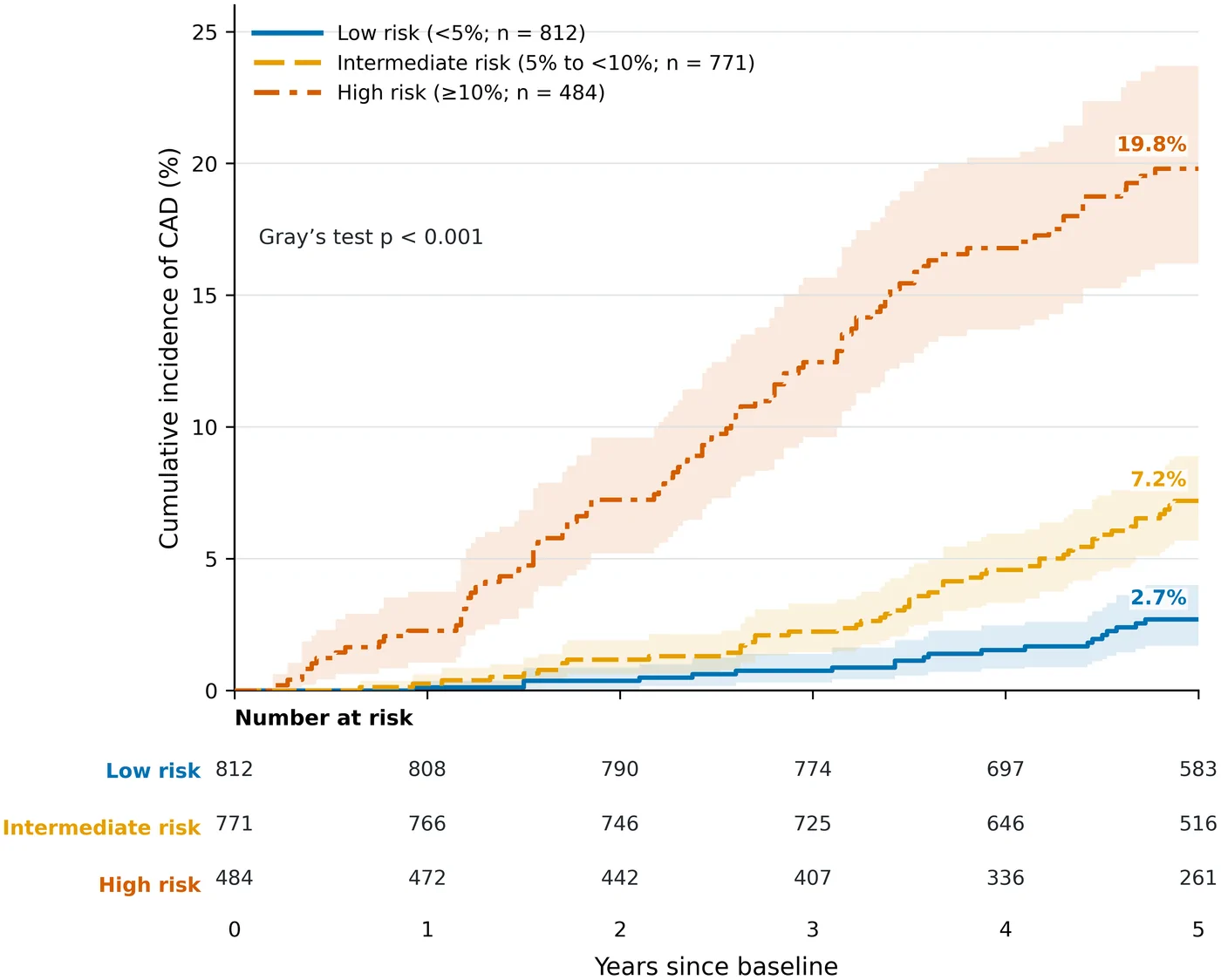
Five-year cumulative incidence according to fixed predicted-risk categories. Aalen–Johansen cumulative incidence functions are shown for participants categorized using pooled out-of-center 5-year predictions as low risk (<5%; *n* = 812), intermediate risk (5% to <10%; *n* = 771), or high risk (≥10%; *n* = 484). Non-coronary death was treated as a competing event. Shaded areas indicate 95% CIs, and numbers at risk are displayed at yearly intervals from baseline through 5 years. The cumulative incidence functions differed across the three categories (Gray's test *p* < 0.001). The categories were used to describe risk gradients and do not represent validated treatment thresholds. CAD, coronary artery disease; CI, confidence interval.

### 3.7 Sensitivity analyses

At the 3-year horizon, the integrated model had a cumulative/dynamic AUC of 0.777 (95% CI, 0.738–0.816) and an IPCW Brier score of 0.047 (95% CI, 0.040–0.054). Its O/E ratio was 1.02 (95% CI, 0.86–1.20), with a calibration intercept of 0.02 (95% CI, −0.17 to 0.21) and a calibration slope of 0.96 (95% CI, 0.76–1.16). The corresponding discrimination, prediction-error, and calibration estimates for the clinical XGBoost, PRS-only XGBoost, and cause-specific Cox clinical models are reported in Supplementary Table S9. No risk categories were defined at 3 years, and the 5-year risk thresholds were not applied to the 3-year predictions.

For the hard coronary endpoint, 141 events were observed over complete follow-up, including 103 events within 5 years. The integrated model was redeveloped and validated independently within each leave-one-center-out cycle using non-fatal MI or coronary death as the event of interest. Based on pooled out-of-center predictions, the 5-year AUC was 0.770 (95% CI, 0.728–0.812), and the IPCW Brier score was 0.049 (95% CI, 0.042–0.056). The O/E ratio was 1.03 (95% CI, 0.86–1.22), the calibration intercept was 0.03 (95% CI, −0.16–0.22), and the calibration slope was 0.95 (95% CI, 0.75–1.15). Across held-out centers, the AUC ranged from 0.736 to 0.790 and the Brier score from 0.045 to 0.052 (Supplementary Table S10).

## 4 Discussion

This multicenter retrospective cohort study evaluated clinical and genetic prediction of incident CAD using a prespecified 5-year horizon, censoring-aware methods, competing-risk estimation, and leave-one-center-out internal–external validation. The PC-adjusted PRS was independently associated with the primary composite endpoint and the hard coronary endpoint. The integrated XGBoost model achieved moderate discrimination, calibration close to ideal, and lower prediction error than the PRS-only and cause-specific Cox clinical models. Its improvement over the clinical XGBoost model was modest, with confidence intervals for differences in AUC and Brier score including the null. Performance also varied across held-out centers. SHAP analysis identified PRS, age, and LDL-C as the three highest-ranked predictors and showed a PRS–LDL-C interaction pattern that was directionally consistent with the prespecified Fine–Gray interaction analysis. Fixed predicted-risk categories separated participants into groups with distinct 5-year cumulative incidences, but these categories were evaluated as descriptive risk strata rather than treatment thresholds.

Each 1-SD increase in the full-cohort PC-adjusted standardized PRS was associated with a 37% higher subdistribution hazard of the primary endpoint and a 42% higher subdistribution hazard of the hard coronary endpoint. These results are consistent with the established association between genome-wide polygenic susceptibility and incident CAD reported in large predominantly European-ancestry cohorts (19). Previous evaluations have nevertheless produced differing estimates of incremental predictive value. Elliott et al. observed a modest improvement when a CAD PRS was added to the pooled cohort equations, whereas Mosley et al. found no statistically significant improvement in the C statistic or risk reclassification in two US cohorts (37, 38). Inouye et al. similarly demonstrated strong association between a genome-wide score and incident CAD, while emphasizing that genetic and conventional risk factors provide complementary information (39). The present effect estimates are lower than those reported for some European-derived scores, which may reflect differences in ancestry, endpoint definition, baseline risk, follow-up, and score coverage.

Our findings should also be interpreted alongside evidence from Chinese populations. In the China Kadoorie Biobank, existing and newly developed PRSs were associated with hard CAD but produced only minimal improvements in discrimination when added to conventional risk factors (40). This observation is compatible with the modest AUC difference between the integrated and clinical XGBoost models in the present study. GPS_CAD was originally derived primarily from European-ancestry genetic data, and differences in allele frequency and linkage disequilibrium can reduce predictive performance after transfer to East Asian populations (41, 42). Residualizing the score against genetic PCs controlled population structure within this cohort but could not restore ancestry-specific genetic information absent from the original score. Recent multi-ancestry approaches have improved CAD risk prediction across several ancestral groups and provide a relevant direction for future score development (43). Accordingly, the observed association supports the prognostic relevance of GPS_CAD in this cohort but does not establish equivalent portability across broader East Asian populations.

The concordant PRS–LDL-C pattern identified by SHAP and Fine–Gray regression warrants cautious interpretation. In the regression model, each 1 mmol/L higher LDL-C multiplied the sHR associated with a 1-SD higher PRS by 1.12. This estimate indicates statistical interaction on the multiplicative subdistribution-hazard scale. It does not demonstrate biological synergy, causality, or differential treatment response. Previous studies have shown that lifestyle factors remain associated with coronary risk across genetic-risk strata and that lifelong exposure to lower LDL-C is associated with lower cardiovascular risk (44, 45). These findings provide general clinical context for considering inherited susceptibility together with modifiable risk factors, but they do not validate the observed model interaction or establish that participants with higher PRS would obtain greater benefit from lipid-lowering therapy. Confirmation requires independent cohorts and analyses designed specifically to assess treatment-effect heterogeneity.

The integrated model had a 5-year AUC of 0.779, compared with 0.762 for the clinical XGBoost model, 0.681 for the PRS-only model, and 0.748 for the cause-specific Cox clinical model. Prior studies have reported that machine-learning methods can capture nonlinear predictor relationships and sometimes improve cardiovascular prediction compared with conventional approaches (6, 7, 46). In the present study, however, the integrated model's AUC difference from the clinical XGBoost model was 0.017, with a 95% CI from −0.003 to 0.038, and the corresponding Brier-score difference was also small. The results therefore support a limited incremental contribution of the PRS rather than clear superiority of the integrated model. The relatively weak performance of the PRS-only model further indicates that genetic susceptibility should not be considered a substitute for established clinical risk factors. Comparable performance at the 3-year horizon suggests that the principal findings were not restricted to the primary 5-year time point.

Calibration was assessed using the O/E ratio, calibration intercept, calibration slope, smoothed calibration curves, and prediction-error measures. This multidimensional assessment is important because adequate discrimination does not ensure accurate absolute-risk estimates (47). The pooled integrated model showed calibration close to ideal, but center-specific estimates were less precise. Leave-one-center-out validation reduced the risk of obtaining optimistic performance estimates by testing each participant using a model developed without data from that participant's institution. It also allowed heterogeneity across centers to be examined directly, which is a central advantage of internal–external validation in clustered multicenter data (48). Nevertheless, the three hospitals were all tertiary centers in China, and the center-specific 5-year event counts ranged from 40 to 72. These results provide evidence of within-study geographic transportability but should not be interpreted as external validation in a different healthcare system or target population.

China-PAR was included as a secondary comparison because it is an established risk algorithm developed for Chinese adults (29). Its original target is 10-year ASCVD rather than 5-year CAD, so the comparison was restricted to ranking performance in eligible participants and did not assess 5-year calibration, Brier score, or DCA. Within the common subset, the integrated model had a higher 5-year AUC than the China-PAR score, but the difference in prediction targets precludes concluding that it replaces China-PAR for its intended use. DCA showed only a small numerical net-benefit advantage for the integrated model over the clinical model at thresholds of approximately 8% to 15%. Decision-curve findings depend on a clearly specified prediction horizon, clinical action, and relative consequences of false-positive and false-negative decisions (49, 50). The present curves therefore suggest potential value for referral to further cardiovascular risk assessment and intensified management of modifiable risk factors, but they do not demonstrate improved clinical outcomes or justify automatic initiation of medication.

SHAP analysis provided complementary global and participant-level descriptions of model behavior. The summary plot established the feature ranking used in the Results, while the waterfall plots illustrated how predictor contributions combined for representative low-risk and high-risk participants. SHAP values offer a consistent method for decomposing predictions from tree-based models (33). They do not establish that a feature causes an outcome, that the model has learned a biological mechanism, or that an explanation will remain stable in another population. Concerns about equating *post hoc* explanations with clinical reliability are particularly relevant when explanations are derived from correlated clinical and genetic predictors (51). The SHAP findings should therefore be regarded as descriptions of the fitted model that support inspection and hypothesis generation, not as evidence sufficient for patient-level clinical decisions.

The competing-risk framework was important because non-coronary death precludes subsequent observation of the primary CAD endpoint. Aalen–Johansen estimation, cause-specific prediction models, and Fine–Gray association analyses address different aspects of this setting and avoid interpreting competing deaths as ordinary independent censoring (31, 52). The association between PRS and CAD was also observed for the hard coronary endpoint comprising non-fatal MI and coronary death. This sensitivity analysis reduces dependence on revascularization, which may be influenced by local diagnostic and procedural practices. The primary composite nevertheless remains clinically relevant because it captures major coronary manifestations encountered in practice. Use of coronary rather than general cardiovascular death, prespecified component hierarchy, standardized definitions, blinded adjudication, and the observed *κ* of 0.80 strengthened endpoint consistency (13, 14).

Additional strengths include retention of all eligible participants regardless of observed follow-up duration, specification of a fixed prediction horizon, separation of development and validation centers during all data-dependent preprocessing, and averaging predictions across 20 imputed datasets. PRS construction, allele harmonization, score coverage, random seeds, model tuning, and output verification were documented to facilitate reproducibility in accordance with PRS reporting standards and TRIPOD + AI guidance (23, 36). The independent repetition of cohort construction, endpoint counting, PRS processing, modeling, and numerical output checking also reduced the likelihood that isolated coding or transcription errors remained in the reported analyses.

Several limitations remain. First, retrospective extraction from EHRs is susceptible to incomplete documentation, measurement heterogeneity, and residual confounding. Multiple imputation reduces loss of information but depends on the missing-at-random assumption. Second, eligibility required linked genotyping data and at least one cardiovascular risk factor, and all centers were tertiary hospitals. The cohort therefore does not represent an unselected community population. Third, internal–external validation across three centers is stronger than a random split but does not replace prospective external validation. The limited numbers of 5-year events within individual centers constrain the precision of center-specific calibration and DCA. Fourth, GPS_CAD was derived mainly from European-ancestry data, only 75.2% of its variants were included in the common scoring set, and PC adjustment does not resolve cross-ancestry portability. Fifth, the composite endpoint includes revascularization and may remain partly sensitive to practice patterns despite standardized adjudication. Sixth, the China-PAR comparison was limited by different prediction horizons and outcomes and by availability of its required variables. Seventh, the risk categories were not optimized or prospectively evaluated as clinical thresholds. Finally, SHAP explanations and the PRS–LDL-C interaction analysis do not establish causal mechanisms or treatment benefit.

Future studies should evaluate the model prospectively in independent Chinese and other East Asian cohorts, assess recalibration where baseline incidence differs, and compare European-derived, East Asian-specific, and multi-ancestry PRSs. Larger validation samples are needed to estimate center-level calibration and net benefit more precisely. Clinical impact studies should then determine whether providing integrated risk estimates changes preventive management, improves patient outcomes, or causes unintended consequences before routine implementation is considered.

## 5 Conclusion

In this three-center retrospective cohort, integration of a PC-adjusted PRS with clinical predictors produced moderate 5-year CAD discrimination, calibration close to ideal during internal–external validation, and a modest improvement over the clinical model. The PRS was independently associated with both the composite and hard coronary endpoints, while the PRS–LDL-C findings represented a model-based interaction pattern rather than evidence of causality or treatment response. Prospective external validation and clinical-impact evaluation are required before the model can support routine clinical decision-making.

## Data Availability

The raw data supporting the conclusions of this article will be made available by the authors, without undue reservation.
