# Supplementary material for: Development and internal–external validation of an interpretable machine learning model integrating clinical and polygenic predictors for coronary artery disease: a multicenter retrospective cohort study
Source: Front Cardiovasc Med. 2026 Sep 24;13:1936899. doi: 10.3389/fcvm.2026.1936899 (PMC13645527; doi:10.3389/fcvm.2026.1936899)
Supplement: Supplementary file 1 [file Datasheet1.pdf]

TRIPOD+AI

Section/Topic	Item	Development / evaluation[1]	Checklist item	Reported on page
**TITLE**				
Title	1	D;E	Identify the study as developing or evaluating the performance of a multivariable prediction model, the target population, and the outcome to be predicted	Lines 1-3
**ABSTRACT**				
Abstract	2	D;E	See TRIPOD+AI for Abstracts checklist	Lines 19-47
**INTRODUCTION**				
Background	3a	D;E	Explain the healthcare context (including whether diagnostic or prognostic) and rationale for developing or evaluating the prediction model, including references to existing models	Lines 53-91
	3b	D;E	Describe the target population and the intended purpose of the prediction model in the context of the care pathway, including its intended users (e.g., healthcare professionals, patients, public)	Lines 92-101 376-379
	3c	D;E	Describe any known health inequalities between sociodemographic groups	Lines 620-633 714-724
Objectives	4	D;E	Specify the study objectives, including whether the study describes the development or validation of a prediction model (or both)	Lines 92-101
**METHODS**				
Data	5a	D;E	Describe the sources of data separately for the development and evaluation datasets (e.g., randomised trial, cohort, routine care or registry data), the rationale for using these data, and representativeness of the data	Lines 103-118
	5b	D;E	Specify the dates of the collected participant data, including start and end of participant accrual; and, if applicable, end of follow-up	Lines 108-115
Participants	6a	D;E	Specify key elements of the study setting (e.g., primary care, secondary care, general population) including the number and location of centres	Lines 105-110
	6b	D;E	Describe the eligibility criteria for study participants	Lines 124-150
	6c	D;E	Give details of any treatments received, and how they were handled during model development or evaluation, if relevant	Lines 189-203
Data preparation	7	D;E	Describe any data pre-processing and quality checking, including whether this was similar across relevant sociodemographic groups	Lines 125-130 203-263; 286-302
Outcome	8a	D;E	Clearly define the outcome that is being predicted and the time horizon, including how and when assessed, the rationale for choosing this outcome, and whether the method of outcome assessment is consistent across sociodemographic groups	Lines 152-182
	8b	D;E	If outcome assessment requires subjective interpretation, describe the qualifications and demographic characteristics of the outcome assessors	Lines 183-188 424-427
	8c	D;E	Report any actions to blind assessment of the outcome to be predicted	Lines 183-188
Predictors	9a	D	Describe the choice of initial predictors (e.g., literature, previous models, all available predictors) and any pre-selection of predictors before model building	Lines 189-199 303-308
	9b	D;E	Clearly define all predictors, including how and when they were measured (and any actions to blind assessment of predictors for the outcome and other predictors)	Lines 189-203 Table S1
	9c	D;E	If predictor measurement requires subjective interpretation, describe the qualifications and demographic characteristics of the predictor assessors	N/A
Sample size	10	D;E	Explain how the study size was arrived at (separately for development and evaluation), and justify that the study size was sufficient to answer the research question. Include details of any sample size calculation	Lines 266-283
Missing data	11	D;E	Describe how missing data were handled. Provide reasons for omitting any data	Lines 143-150 286-302; Table S3
Analytical methods	12a	D	Describe how the data were used (e.g., for development and evaluation of model performance) in the analysis, including whether the data were partitioned, considering any sample size requirements	Lines 105-118 286-302
	12b	D	Depending on the type of model, describe how predictors were handled in the analyses (functional form, rescaling, transformation, or any standardisation).	Lines 247-261 300-321
	12c	D	Specify the type of model, rationale[2], all model-building steps, including any hyperparameter tuning, and method for internal validation	Lines 303-359
	12d	D;E	Describe if and how any heterogeneity in estimates of model parameter values and model performance was handled and quantified across clusters (e.g., hospitals, countries). See TRIPOD-Cluster for additional considerations[3]	Lines 361-375 416-423
	12e	D;E	Specify all measures and plots used (and their rationale) to evaluate model performance (e.g., discrimination, calibration, clinical utility) and, if relevant, to compare multiple models	Lines 360-383
	12f	E	Describe any model updating (e.g., recalibration) arising from the model evaluation, either overall or for particular sociodemographic groups or settings	N/A; no updating
	12g	E	For model evaluation, describe how the model predictions were calculated (e.g., formula, code, object, application programming interface)	Lines 328-353 361-383
Class imbalance	13	D;E	If class imbalance methods were used, state why and how this was done, and any subsequent methods to recalibrate the model or the model predictions	N/A; not used
Fairness	14	D;E	Describe any approaches that were used to address model fairness and their rationale	Lines 213-217 247-255; 714-724
Model output	15	D	Specify the output of the prediction model (e.g., probabilities, classification). Provide details and rationale for any classification and how the thresholds were identified	Lines 328-333 376-380; 404-409

[1] D=items relevant only to the development of a prediction model; E=items relating solely to the evaluation of a prediction model; D;E=items applicable to both the development and evaluation of a prediction model

[2] Separately for all model building approaches.

[3] TRIPOD-Cluster is a checklist of reporting recommendations for studies developing or validating models that explicitly account for clustering or explore heterogeneity in model performance (eg, at different hospitals or centres). Debray et al, BMJ 2023; 380: e071018 [DOI: 10.1136/bmj-2022-071018]

Training versus evaluation	16	D;E	Identify any differences between the development and evaluation data in healthcare setting, eligibility criteria, outcome, and predictors	Lines 456-482<br>Table 1
Ethical approval	17	D;E	Name the institutional research board or ethics committee that approved the study and describe the participant-informed consent or the ethics committee waiver of informed consent	Lines 118-122<br>748-752
**OPEN SCIENCE**				
Funding	18a	D;E	Give the source of funding and the role of the funders for the present study	Lines 764-766
Conflicts of interest	18b	D;E	Declare any conflicts of interest and financial disclosures for all authors	Lines 762-763
Protocol	18c	D;E	Indicate where the study protocol can be accessed or state that a protocol was not prepared	No public protocol
Registration	18d	D;E	Provide registration information for the study, including register name and registration number, or state that the study was not registered	Not registered
Data sharing	18e	D;E	Provide details of the availability of the study data	Lines 755-761
Code sharing	18f	D;E	Provide details of the availability of the analytical code[4]	Lines 447-453<br>755-757
**PATIENT & PUBLIC INVOLVEMENT**				
Patient & Public Involvement	19	D;E	Provide details of any patient and public involvement during the design, conduct, reporting, interpretation, or dissemination of the study or state no involvement.	No PPI
**RESULTS**				
Participants	20a	D;E	Describe the flow of participants through the study, including the number of participants with and without the outcome and, if applicable, a summary of the follow-up time. A diagram may be helpful.	Lines 456-465<br>Figure 1
	20b	D;E	Report the characteristics overall and, where applicable, for each data source or setting, including the key dates, key predictors (including demographics), treatments received, sample size, number of outcome events, follow-up time, and amount of missing data. A table may be helpful. Report any differences across key demographic groups.	Lines 456-469<br>Table 1; Table S3
	20c	E	For model evaluation, show a comparison with the development data of the distribution of important predictors (demographics, predictors, and outcome).	Lines 466-482<br>Table 1; Figs S1-S2
Model development	21	D;E	Specify the number of participants and outcome events in each analysis (e.g., for model development, hyperparameter tuning, model evaluation)	Lines 282-283<br>485-501; 528-529
Model specification	22	D	Provide details of the full prediction model (e.g., formula, code, object, application programming interface) to allow predictions in new individuals and to enable third-party evaluation and implementation, including any restrictions to access or re-use (e.g., freely available, proprietary)[5]	Lines 322-353<br>447-453; File S4
Model performance	23a	D;E	Report model performance estimates with confidence intervals, including for any key subgroups (e.g., sociodemographic). Consider plots to aid presentation.	Lines 527-550<br>Table 3; Figure 4
	23b	D;E	If examined, report results of any heterogeneity in model performance across clusters. See TRIPOD Cluster for additional details[3].	Lines 539-541<br>Table S7; Figure S3
Model updating	24	E	Report the results from any model updating, including the updated model and subsequent performance	N/A; no updating
**DISCUSSION**				
Interpretation	25	D;E	Give an overall interpretation of the main results, including issues of fairness in the context of the objectives and previous studies	Lines 593-713
Limitations	26	D;E	Discuss any limitations of the study (such as a non-representative sample, sample size, overfitting, missing data) and their effects on any biases, statistical uncertainty, and generalizability	Lines 714-729
Usability of the model in the context of current care	27a	D	Describe how poor quality or unavailable input data (e.g., predictor values) should be assessed and handled when implementing the prediction model	Not implemented<br>Lines 730-736
	27b	D	Specify whether users will be required to interact in the handling of the input data or use of the model, and what level of expertise is required of users	Not implemented<br>Lines 730-736
	27c	D;E	Discuss any next steps for future research, with a specific view to applicability and generalizability of the model	Lines 730-745

From: Collins GS, Moons KGM, Dhiman P, et al. *BMJ* 2024;385:e078378. doi:10.1136/bmj-2023-078378

[4] This relates to the analysis code, for example, any data cleaning, feature engineering, model building, evaluation.
[5] This relates to the code to implement the model to get estimates of risk for a new individual.
