# Supplementary material for: Development and internal–external validation of an interpretable machine learning model integrating clinical and polygenic predictors for coronary artery disease: a multicenter retrospective cohort study
Source: Front Cardiovasc Med. 2026 Sep 24;13:1936899. doi: 10.3389/fcvm.2026.1936899 (PMC13645527; doi:10.3389/fcvm.2026.1936899)
Supplement: Supplementary file 2 [file Supplementaryfile1.docx]

**Supplementary File S1**

**Endpoint identification, coding algorithms, component definitions, and adjudication procedures**

# 1. Purpose and scope

This supplement specifies the common endpoint-identification and adjudication protocol used at Wuxi No. 2 People’s Hospital, The Second Affiliated Hospital of Hainan Medical University, and The Affiliated Central Hospital of Jiangnan University. Coding records were used to identify potential events for clinical review; no diagnosis or procedure code alone established an endpoint. All candidate events were verified from the available clinical record using the definitions below.

The primary endpoint was the first occurrence after baseline of non-fatal myocardial infarction (MI), ischemia-driven coronary revascularization, or coronary death. Baseline was the date of the first eligible clinical evaluation. The primary prediction horizon was 5 years, and the 3-year horizon was used in a sensitivity analysis.

# 2. Common electronic retrieval algorithm

At each center, the same prespecified query searched structured and unstructured electronic records from baseline through the first potential endpoint, non-coronary death, last documented clinical contact, or December 31, 2024, whichever occurred first. The query retrieved the following evidence domains:

- inpatient and outpatient diagnosis codes, principal and secondary diagnoses, and discharge diagnoses;
- procedure codes and names, catheterization laboratory records, coronary angiography reports, PCI reports, and cardiac surgical operative reports;
- serial cardiac troponin results with assay-specific 99th-percentile upper reference limits;
- electrocardiograms, echocardiography and other cardiac imaging reports, emergency notes, admission notes, progress notes, and discharge summaries; and
- death certificates, hospital death summaries, resuscitation records, and linked institutional death records when available.

Records occurring within the same hospitalization or within 24 hours of one another were consolidated into one candidate-event packet. Duplicate records were removed using the de-identified study identifier, encounter date, event date, and source type. The packet retained the original source code and text, the normalized code, relevant dates, and the clinical evidence required for adjudication.

# 3. Myocardial infarction

## 3.1 ICD-10 screening codes

| **ICD-10 code** | **Code description** | **Use in the algorithm** |
| --- | --- | --- |
| I21 | Acute myocardial infarction | Parent-category trigger; a more specific local descendant was retained when available. |
| I21.0 | Acute transmural myocardial infarction of anterior wall | Potential MI |
| I21.1 | Acute transmural myocardial infarction of inferior wall | Potential MI |
| I21.2 | Acute transmural myocardial infarction of other sites | Potential MI |
| I21.3 | Acute transmural myocardial infarction of unspecified site | Potential MI |
| I21.4 | Acute subendocardial myocardial infarction | Potential MI |
| I21.9 | Acute myocardial infarction, unspecified | Potential MI |
| I22 | Subsequent myocardial infarction | Parent-category trigger; a more specific local descendant was retained when available. |
| I22.0 | Subsequent myocardial infarction of anterior wall | Potential MI |
| I22.1 | Subsequent myocardial infarction of inferior wall | Potential MI |
| I22.8 | Subsequent myocardial infarction of other sites | Potential MI |
| I22.9 | Subsequent myocardial infarction of unspecified site | Potential MI |

**Note:** I21.x and I22.x were screening triggers. Decimal-omitted forms and center-specific descendants were normalized to the corresponding parent code, but every potential MI required clinical adjudication.

## 3.2 Clinical confirmation

A non-fatal MI required acute myocardial injury together with evidence of acute myocardial ischemia in accordance with the Fourth Universal Definition of Myocardial Infarction [1]. Acute myocardial injury was defined by a rise and/or fall in cardiac troponin, with at least one value above the assay-specific 99th-percentile upper reference limit. At least one of the following ischemic criteria was also required:

- symptoms consistent with acute myocardial ischemia;
- new ischemic electrocardiographic changes;
- development of pathological Q waves;
- imaging evidence of new loss of viable myocardium or a new regional wall-motion abnormality in a pattern consistent with ischemia; or
- identification of coronary thrombus by angiography or autopsy, when applicable.

Type 3 MI was considered only when cardiac death occurred with symptoms or electrocardiographic evidence suggesting acute ischemia before biomarkers could be obtained or before biomarker elevation was detectable. Because the primary component was non-fatal MI, a qualifying fatal presentation was classified as coronary death under the component hierarchy. PCI-related and CABG-related MI were accepted only when the corresponding procedure-related criteria in the Fourth Universal Definition were met; isolated postprocedural troponin elevation was insufficient.

Isolated myocardial injury without evidence of acute ischemia was not classified as MI. This included chronic or non-ischemic troponin elevation attributable to renal dysfunction, heart failure, sepsis, pulmonary embolism, tachyarrhythmia, myocarditis, or another documented non-coronary condition unless the full MI criteria were independently satisfied.

# 4. Coronary revascularization

## 4.1 ICD-9-CM procedure codes

| **Procedure** | **ICD-9-CM code** | **Full code description** |
| --- | --- | --- |
| PCI | 00.66 | Percutaneous transluminal coronary angioplasty or coronary atherectomy |
| PCI | 36.01 | Single-vessel percutaneous transluminal coronary angioplasty without thrombolytic agent |
| PCI | 36.02 | Single-vessel percutaneous transluminal coronary angioplasty with thrombolytic agent |
| PCI | 36.05 | Multiple-vessel percutaneous transluminal coronary angioplasty performed during the same operation, with or without thrombolytic agent |
| PCI | 36.06 | Insertion of non-drug-eluting coronary artery stent(s) |
| PCI | 36.07 | Insertion of drug-eluting coronary artery stent(s) |
| PCI | 36.09 | Other removal of coronary artery obstruction |
| CABG | 36.10 | Aortocoronary bypass for heart revascularization, not otherwise specified |
| CABG | 36.11 | Aortocoronary bypass of one coronary artery |
| CABG | 36.12 | Aortocoronary bypass of two coronary arteries |
| CABG | 36.13 | Aortocoronary bypass of three coronary arteries |
| CABG | 36.14 | Aortocoronary bypass of four or more coronary arteries |
| CABG | 36.15 | Single internal mammary-coronary artery bypass |
| CABG | 36.16 | Double internal mammary-coronary artery bypass |
| CABG | 36.17 | Abdominal-coronary artery bypass |
| CABG | 36.19 | Other bypass anastomosis for heart revascularization |

**Note:** Codes 36.01, 36.02, and 36.05 were retained as legacy screening codes because historical and locally mapped records could contain them. Procedure-code titles were checked against the CMS ICD-9-CM code files [3].

## 4.2 Procedure verification

A coded PCI or CABG record was accepted as a performed procedure only when the catheterization report, PCI report, operative report, or discharge summary documented the procedure and its date. Diagnostic coronary angiography without an intervention, planned procedures that were cancelled, non-coronary vascular interventions, and procedure codes unsupported by the clinical record were excluded.

## 4.3 Ischemia-driven classification

A verified PCI or CABG qualified as ischemia-driven when either of the following conditions was documented [2]:

- the procedure was performed for an acute coronary syndrome; or
- coronary angiography demonstrated at least 50% diameter stenosis in a major epicardial coronary artery or branch, together with ischemic symptoms or objective evidence of myocardial ischemia.

Objective ischemia could be documented by ischemic electrocardiographic changes, stress electrocardiography, stress echocardiography, myocardial perfusion imaging, stress cardiac magnetic resonance imaging, or an invasive physiological assessment supporting lesion-specific ischemia. Revascularization performed solely for surveillance findings or an incidentally identified lesion without an acute coronary syndrome, ischemic symptoms, or objective ischemia did not qualify. For a staged procedure, the initial ischemic indication remained valid when the procedure was explicitly documented as completion of the same treatment plan.

# 5. Coronary death

Cause of death was assigned from the underlying disease or injury that initiated the sequence leading to death, rather than the terminal physiological mode of death [2]. A death was classified as coronary death when the available evidence supported one of the following:

- death attributable to an adjudicated acute MI, including a fatal complication or a procedure undertaken to treat that MI;
- death attributable to ischemic heart disease documented as the underlying cause, supported by the terminal clinical course, prior coronary findings, or relevant investigations; or
- sudden cardiac death with clinical or investigative evidence supporting a coronary cause, including a witnessed unexpected death or documented malignant ventricular arrhythmia without a more plausible non-coronary cause, or an unwitnessed death in a person known to be clinically stable within 24 hours when the available record supported a coronary mechanism and did not identify another cause.

A death certificate code or the nonspecific mode of death alone was insufficient. Death due to stroke, heart failure without evidence of an underlying coronary cause, pulmonary embolism, aortic disease, infection, malignancy, trauma, renal or hepatic failure, or another identified non-coronary cause was not classified as coronary death. When available evidence was insufficient to support a coronary cause, the death was classified as non-coronary death for the competing-risk analysis, consistent with the prespecified study endpoint.

# 6. Center-specific code normalization and extension mapping

The same normalization rules were applied at all three centers before clinical review. The original code and description were retained in the audit dataset. Normalization removed leading and trailing spaces, converted letters to uppercase, converted full-width punctuation to half-width punctuation, and restored the canonical decimal position when absent. A center-specific suffix was collapsed to its canonical parent only when its local dictionary description and the associated clinical record referred to the same diagnosis or procedure. Free-text terms could trigger retrieval but never confirmed an endpoint without source-document verification.

| **Domain** | **Canonical codes** | **Forms accepted at Wuxi, Hainan, and Jiangnan** | **Stored normalized value** |
| --- | --- | --- | --- |
| MI | I21, I21.0, I21.1, I21.2, I21.3, I21.4, I21.9; I22, I22.0, I22.1, I22.8, I22.9 | Exact code; decimal-omitted form (e.g., I214); locally suffixed descendant of I21.x or I22.x when dictionary text agreed | Canonical I21.x or I22.x code |
| PCI | 00.66; 36.01; 36.02; 36.05; 36.06; 36.07; 36.09 | Exact code; decimal-omitted form (e.g., 0066, 3607); locally suffixed descendant when procedure text agreed | Canonical PCI code |
| CABG | 36.10–36.17; 36.19 | Exact code; decimal-omitted form (e.g., 3611); locally suffixed descendant when procedure text agreed | Canonical CABG code |

**Note:** No center used an additional diagnosis or procedure family outside the prespecified canonical sets. An unmapped code or a text-only match was retained as a review trigger and required confirmation from the clinical source record.

# 7. Event date and component hierarchy

| **Situation** | **Prespecified rule** |
| --- | --- |
| Single qualifying component | Use the date on which that component first met its definition. |
| Components on different dates | Use the earliest qualifying date and classify the first qualifying component. |
| Multiple components on the same calendar day | Classify in the following order: coronary death, non-fatal MI, ischemia-driven coronary revascularization. |
| MI and PCI/CABG on the same day | Classify as non-fatal MI unless a qualifying coronary death occurred that day. |
| PCI and CABG on the same day without MI or coronary death | Classify once as ischemia-driven coronary revascularization; record both procedures in the supporting fields. |
| Hard coronary endpoint | Use the first non-fatal MI or coronary death. Isolated revascularization is not an event, and follow-up continues. |

# 8. Competing events, censoring, and risk horizons

Death from any cause other than an adjudicated coronary death before the primary endpoint was the competing event. The competing-event date was the date of death. A participant without a primary or competing event was censored at the last documented clinical contact or December 31, 2024, whichever occurred first. Administrative database activity without evidence of patient contact was not used to extend follow-up.

The primary 5-year analysis used events and competing deaths occurring within 5 years after baseline and treated earlier loss to follow-up as censoring. The 3-year sensitivity analysis applied the same definitions through 3 years. For the hard coronary endpoint, follow-up continued after isolated coronary revascularization until non-fatal MI, coronary death, non-coronary death, censoring, or the administrative end of follow-up.

# 9. Standardized three-center adjudication workflow

1. The local data team ran the common electronic query and retained the original and normalized diagnosis/procedure codes, event dates, source-document types, and relevant clinical evidence.
2. Potential records from the same clinical episode were deduplicated and assembled into a de-identified candidate-event packet.
3. A completeness check confirmed that the available biomarker, electrocardiographic, imaging, angiographic, operative, discharge, and death documentation had been retrieved. Absence of a source was recorded explicitly.
4. Two cardiologists independently reviewed the same packet using the standardized form. They were blinded to baseline predictors, PRS, model predictions, and each other’s assessments.
5. Each cardiologist independently classified the record as non-fatal MI, ischemia-driven coronary revascularization, coronary death, non-coronary death, no qualifying event, or insufficient evidence, and recorded the event date and supporting criteria.
6. Concordant classifications were accepted. Discordant classifications, event dates, or component assignments were submitted to a third senior cardiologist, who was blinded to baseline predictors and model outputs and reviewed the source evidence before assigning the final classification.
7. The final event type and date were locked before preparation of the analysis dataset. The hierarchy in Section 7 was applied algorithmically and checked against the adjudication record.
8. Cross-center quality control verified code normalization, duplicate handling, chronological ordering, component totals, competing deaths, censoring dates, and consistency between the locked adjudication file and analysis dataset.

# 10. Standardized endpoint adjudication form

The form below contains no direct identifiers. Each reviewer received only the de-identified candidate-event packet and study event ID.

| **Field** | **Entry or permitted response** |
| --- | --- |
| Study event ID | De-identified alphanumeric study event identifier |
| Center | Wuxi / Hainan / Jiangnan |
| Baseline date | YYYY-MM-DD |
| Candidate event date(s) | YYYY-MM-DD; list all relevant dates |
| Retrieval trigger | ICD-10 diagnosis / ICD-9-CM procedure / biomarker / ECG / imaging / angiography / operative record / discharge summary / death record / other |
| Source documents available | Mark each available source; record unavailable sources as not available |

## 10.1 MI evidence

| **Field** | **Entry or permitted response** |
| --- | --- |
| Serial cardiac troponin | Rise and/or fall present: Yes / No / Indeterminate; peak value and assay-specific 99th-percentile URL recorded |
| Ischemic symptoms | Present / Absent / Indeterminate |
| New ischemic ECG changes | Present / Absent / Indeterminate |
| Pathological Q waves | Present / Absent / Indeterminate |
| New ischemic imaging abnormality | Present / Absent / Indeterminate |
| Coronary thrombus | Present / Absent / Not assessed |
| Procedure-related MI | No / PCI-related / stent thrombosis / restenosis-related / CABG-related; universal-definition criteria met: Yes / No / Not applicable |
| Alternative cause of myocardial injury | None identified / Specify non-ischemic or non-coronary cause |
| MI conclusion | Qualifying non-fatal MI / Does not qualify / Insufficient evidence |

## 10.2 Revascularization evidence

| **Field** | **Entry or permitted response** |
| --- | --- |
| Procedure | PCI / CABG / Both / No verified coronary intervention |
| Procedure date | YYYY-MM-DD |
| Canonical procedure code(s) | Record normalized ICD-9-CM code(s) and retain original local code(s) in audit dataset |
| Source verification | Catheterization/PCI report / Operative report / Discharge summary |
| Acute coronary syndrome indication | Yes / No / Indeterminate |
| Coronary stenosis | Maximum documented diameter stenosis and vessel; threshold ≥50%: Yes / No / Indeterminate |
| Ischemic symptoms | Present / Absent / Indeterminate |
| Objective ischemia | Present / Absent / Indeterminate; specify test |
| Staged procedure | No / Yes, completion of same documented ischemia-driven treatment plan / Yes, new indication |
| Revascularization conclusion | Qualifying ischemia-driven revascularization / Verified but not ischemia-driven / Does not qualify / Insufficient evidence |

## 10.3 Death evidence

| **Field** | **Entry or permitted response** |
| --- | --- |
| Date of death | YYYY-MM-DD |
| Evidence sources | Death certificate / Hospital death summary / Terminal admission / Resuscitation record / Linked death record / Autopsy / Other |
| Underlying cause | Acute MI / Ischemic heart disease / Sudden cardiac death with coronary evidence / Identified non-coronary cause / Insufficient evidence |
| Coronary supporting evidence | Summarize symptoms, ECG, biomarkers, angiography, imaging, rhythm documentation, recent MI, or other relevant evidence |
| Competing non-coronary cause | Absent / Present; specify |
| Death conclusion | Coronary death / Non-coronary death |

## 10.4 Independent classification and arbitration

| **Field** | **Entry or permitted response** |
| --- | --- |
| Adjudicator A | Primary component; event date; qualifying criteria; non-coronary competing death if applicable |
| Adjudicator B | Primary component; event date; qualifying criteria; non-coronary competing death if applicable |
| Agreement before arbitration | Yes / No |
| Reason for discordance | Endpoint confirmation / Component / Event date / Ischemia-driven status / Cause of death / Other |
| Third-cardiologist arbitration | Required / Not required; final component; final event date; supporting rationale |
| Final locked classification | Non-fatal MI / Ischemia-driven coronary revascularization / Coronary death / Non-coronary death / No qualifying event |
| Hard coronary endpoint | Yes / No; event date if Yes |

# 11. Privacy and audit controls

Endpoint packets and adjudication files used study identifiers and did not contain names, government identifiers, addresses, telephone numbers, or other direct identifiers. Dates were retained only within the approved analytic environment because they were required to establish event order and follow-up time. Access was restricted to authorized study personnel. The audit dataset preserved the original source code, normalized code, evidence flags, independent adjudications, arbitration status, final classification, and event date; it did not expose patient-level data in this supplementary file.

# 12. Abbreviations

CABG, coronary artery bypass grafting; CAD, coronary artery disease; ECG, electrocardiogram; ICD-9-CM, International Classification of Diseases, Ninth Revision, Clinical Modification; ICD-10, International Classification of Diseases, Tenth Revision; MI, myocardial infarction; PCI, percutaneous coronary intervention; URL, upper reference limit.

# 13. References

1. Thygesen K, Alpert JS, Jaffe AS, et al. Fourth Universal Definition of Myocardial Infarction (2018). Circulation. 2018;138(20):e618-e651. doi:10.1161/CIR.0000000000000617

2. Hicks KA, Mahaffey KW, Mehran R, et al. 2017 Cardiovascular and Stroke Endpoint Definitions for Clinical Trials. J Am Coll Cardiol. 2018;71(9):1021-1034. doi:10.1016/j.jacc.2017.12.048

3. Centers for Medicare & Medicaid Services. ICD-9-CM diagnosis and procedure codes: abbreviated and full code titles, Version 32. Effective October 1, 2014. Accessed August 26, 2026. https://www.cms.gov/medicare/coding-billing/icd-10-codes/icd-9-cm-diagnosis-procedure-codes-abbreviated-and-full-code-titles
