# Supplementary material for: Development and internal–external validation of an interpretable machine learning model integrating clinical and polygenic predictors for coronary artery disease: a multicenter retrospective cohort study
Source: Front Cardiovasc Med. 2026 Sep 24;13:1936899. doi: 10.3389/fcvm.2026.1936899 (PMC13645527; doi:10.3389/fcvm.2026.1936899)
Supplement: Supplementary file 3 [file Supplementaryfile2.docx]

**Supplementary File S2**

**Genotype imputation, allele harmonization, and calculation of GPS_CAD**

# 1. Purpose and analytical scope

This supplement describes the reproducible genetic-processing workflow used to calculate the coronary artery disease polygenic risk score from pre-existing Illumina Infinium Global Screening Array data at the three participating centers. The workflow comprised array-level quality control, GRCh37/hg19 coordinate verification, pre-phasing, genotype imputation, post-imputation filtering, harmonization to the published GPS_CAD weights, construction of a fixed cross-center scoring set, raw score calculation, and validation-cycle-specific population-structure adjustment and standardization.

The published score was GPS_CAD (PGS Catalog accession PGS000013), developed by Khera et al. using LDpred with a causal-variant fraction of ρ = 0.001 [1, 2]. The original scoring file was on hg19 and contained 6,630,150 weighted variants. Published effect weights were applied directly and were not re-estimated in the study cohort.

# 2. Software, versions, and fixed resources

| **Software** | **Version** | | **Primary use** |
| --- | --- | --- | --- |
| PLINK | 1.90b6.21 | | Sample and variant quality control, allele frequencies, LD pruning, PCA, and PRS calculation |
| SHAPEIT4 | 4.2.2 | | Autosomal pre-phasing by chromosome |
| Minimac4 | 4.1.6 | | Genotype imputation from phased target haplotypes |
| BCFtools/HTSlib | 1.21 | | VCF/BCF normalization, indexing, concatenation, and field extraction |
| R | 4.5.1 | | PC projection checks, residualization, standardization, and audit summaries |
| Python | 3.11.9 | | Score-file parsing, allele harmonization, and cross-center intersection |
| **Resource** | | **Specification** | |
| Target coordinate system | | GRCh37/hg19 autosomes 1–22 | |
| Score weights | | PGS000013 scoring file; 6,630,150 variants; original build hg19 | |
| Phasing maps | | SHAPEIT4 GRCh37 chromosome-specific genetic maps | |
| Imputation reference | | 1000 Genomes Project Phase 3 East Asian samples, chromosome-specific phased haplotypes | |
| Reference FASTA | | GRCh37/hg19, used for chromosome, position, and reference-allele verification | |
| Random seed | | 20260726 for deterministic steps that accepted a seed | |

# 3. Array-level quality control

Quality control was performed separately at each center before phasing. Directly genotyped variants were filtered before imputation; the 98% call-rate threshold was not reapplied to imputed dosages. The following order was used:

1. Remove samples with genotype call rate <98%.
2. Remove samples with discordance between reported and genetically inferred sex.
3. Remove samples with autosomal heterozygosity outside ±3 SD from the center-specific mean after exclusion of high-LD regions.
4. Remove variants with call rate <98%.
5. Remove directly genotyped variants with minor allele frequency <1%.
6. Remove variants with Hardy–Weinberg equilibrium p < 1.0 × 10⁻⁶.
7. Retain biallelic autosomal variants with unique identifiers and coordinates that could be verified on GRCh37/hg19.

| **Operation** | **Command** |
| --- | --- |
| Sample call rate | plink --bfile ${CENTER}_array --mind 0.02 --make-bed --out ${CENTER}_s1 |
| Sex check | plink --bfile ${CENTER}_s1 --check-sex --out ${CENTER}_sexcheck |
| Heterozygosity | plink --bfile ${CENTER}_s1 --het --out ${CENTER}_het |
| Remove flagged samples and filter variants | plink --bfile ${CENTER}_s1 --remove ${CENTER}_sample_exclusions.txt --geno 0.02 --maf 0.01 --hwe 1e-6 --make-bed --out ${CENTER}_qc |
| Export indexed VCF | plink --bfile ${CENTER}_qc --recode vcf-iid bgz --out ${CENTER}_grch37; bcftools index -t ${CENTER}_grch37.vcf.gz |

**Note:** ${CENTER} was set in turn to wuxi, hainan, or jiangnan. The sex-check and heterozygosity outputs were reviewed to create the two-column sample-exclusion file. Sample identifiers remained de-identified.

# 4. GRCh37/hg19 coordinate and allele verification

Array-marker coordinates were assigned from the platform manifest corresponding to GRCh37/hg19. Chromosome labels were normalized to 1–22, positions were required to be positive integers within the corresponding GRCh37 chromosome, and duplicated marker identifiers were resolved before VCF export. Multiallelic records were split into biallelic records. REF alleles were checked against the GRCh37 reference FASTA; unresolved reference mismatches were flagged and were not carried into imputation. No coordinate liftover was performed after the data had been aligned to GRCh37/hg19.

| **Operation** | **Command** |
| --- | --- |
| Split and normalize | bcftools norm -m -any -f GRCh37.fa -c wx ${CENTER}_grch37.vcf.gz -Oz -o ${CENTER}_grch37.norm.vcf.gz |
| Index | bcftools index -t ${CENTER}_grch37.norm.vcf.gz |
| Coordinate audit | bcftools query -f '%CHROM\t%POS\t%ID\t%REF\t%ALT\n' ${CENTER}_grch37.norm.vcf.gz > ${CENTER}_grch37.variant_audit.tsv |

**Note:** The option -c wx issued a warning and excluded an unresolved reference mismatch. The audit table was retained to trace every imputed and scored variant to its GRCh37/hg19 coordinate.

# 5. Reference panel

The imputation reference comprised the 504 East Asian samples in the 1000 Genomes Project Phase 3 release [3]. These consisted of Han Chinese in Beijing (CHB; n = 103), Japanese in Tokyo (JPT; n = 104), Southern Han Chinese (CHS; n = 105), Chinese Dai in Xishuangbanna (CDX; n = 93), and Kinh in Ho Chi Minh City (KHV; n = 99). Reference variants and haplotypes were maintained on GRCh37/hg19 and separated by chromosome. The EAS panel used for target imputation was distinct from the European LD reference used during the original development of GPS_CAD.

For Minimac4, the chromosome-specific phased EAS reference VCFs were converted to MVCF-compressed .msav files once and indexed. The reference-panel sample set was fixed before target imputation and did not include any study participant.

| **Operation** | **Command** |
| --- | --- |
| Compress a reference chromosome | minimac4 --compress-reference 1KG_Phase3_EAS.chr${CHR}.vcf.gz > 1KG_Phase3_EAS.chr${CHR}.msav |

# 6. Pre-phasing with SHAPEIT4

Each center was phased separately by autosomal chromosome using SHAPEIT4 version 4.2.2 [4]. Input VCF/BCF files and genetic maps used the same GRCh37 chromosome labels. Default SNP-array phasing settings were retained. A single thread and seed 20260726 were used to make the stochastic phasing step reproducible; a log file was written for every center and chromosome.

| **Parameter** | **Specification** |
| --- | --- |
| Input | ${CENTER}_chr${CHR}.unphased.bcf; indexed; GRCh37/hg19 |
| Genetic map | chr${CHR}.b37.gmap.gz |
| Region | ${CHR} |
| MCMC and PBWT settings | SHAPEIT4 4.2.2 defaults for SNP-array data |
| Threads and seed | --thread 1 --seed 20260726 |
| Output | ${CENTER}_chr${CHR}.phased.bcf and chromosome-specific log |
| **Operation** | **Command** |
| Phase one chromosome | shapeit4 --input ${CENTER}_chr${CHR}.unphased.bcf --map chr${CHR}.b37.gmap.gz --region ${CHR} --seed 20260726 --thread 1 --output ${CENTER}_chr${CHR}.phased.bcf --log ${CENTER}_chr${CHR}.shapeit4.log |

Phased outputs were checked for successful completion, nonzero variant and sample counts, unchanged sample identifiers, and absence of unexpected chromosome labels. The phased BCF was indexed before imputation.

# 7. Imputation with Minimac4

Phased target genotypes were imputed separately by center and chromosome using Minimac4 version 4.1.6 [5]. The matching 1000 Genomes Phase 3 EAS .msav file served as the reference. Genotype dosages (DS) and typed sites were retained. No study outcome, clinical predictor, or cross-center phenotype information entered the imputation step.

| **Parameter** | **Specification** |
| --- | --- |
| Reference | 1KG_Phase3_EAS.chr${CHR}.msav |
| Target | ${CENTER}_chr${CHR}.phased.bcf |
| Output fields | GT and DS; sites file containing the Minimac4 imputation-quality estimate |
| Typed variants | Retained with --all-typed-sites |
| Threads | 8; imputation is deterministic for fixed phased inputs and reference files |
| Output | Chromosome-specific compressed VCF plus sites file and execution log |
| **Operation** | **Command** |
| Impute one chromosome | minimac4 --threads 8 --format GT,DS --all-typed-sites 1KG_Phase3_EAS.chr${CHR}.msav ${CENTER}_chr${CHR}.phased.bcf -o ${CENTER}_chr${CHR}.imputed.vcf.gz -s ${CENTER}_chr${CHR}.imputed.sites.vcf.gz |

**Note:** The Minimac4 imputation-quality estimate exported in the sites file was carried into the harmonization table under the analysis field INFO.

# 8. Post-imputation variant quality control

Filtering was applied sequentially within each center and to the cross-center common set. Variants had to be present after imputation, have INFO ≥0.80, and have dosage-derived MAF ≥1%. The MAF threshold was evaluated after the INFO filter. Imputed dosages were not converted to hard calls for PRS calculation.

| **Operation** | **Command** |
| --- | --- |
| Dosage allele frequencies | plink --vcf ${CENTER}_chr${CHR}.imputed.vcf.gz dosage=DS --freq --out ${CENTER}_chr${CHR}.dosage_freq |
| Extract retained variants | bcftools view -R ${CENTER}_chr${CHR}.info08_maf01.positions.tsv ${CENTER}_chr${CHR}.imputed.vcf.gz -Oz -o ${CENTER}_chr${CHR}.postqc.vcf.gz |
| Index retained VCF | bcftools index -t ${CENTER}_chr${CHR}.postqc.vcf.gz |

**Note:** The retained-position file was generated by joining the sites-file INFO values to dosage-derived MAF values using chromosome, position, REF, and ALT.

# 9. GPS_CAD scoring weights

The PGS000013 scoring file was obtained from the PGS Catalog and used under its stated research-use terms [1, 2]. The file was parsed into chromosome, GRCh37 position, effect allele, other allele, and published log-odds weight. Rows without a finite weight or valid autosomal coordinate were not eligible. The score file checksum and retrieval date were stored in the reproducibility manifest supplied with the complete code.

GPS_CAD was originally generated with LDpred using ρ = 0.001 and an LD panel of 503 European samples [1, 2]. The present analysis did not reconstruct the score by clumping and thresholding and did not fit new genetic weights in the target cohort.

# 10. Allele harmonization

Harmonization was performed independently at each center on GRCh37/hg19 using chromosome, position, REF allele, ALT allele, effect allele, and other allele. Each eligible score row was assigned exactly one of the following mutually exclusive statuses:

| **Status** | **Operational rule** |
| --- | --- |
| Direct | Target alleles matched the scoring alleles in the same strand orientation; the dosage of the published effect allele was used. |
| Allele swap | Target REF/ALT order was reversed relative to the scoring row; dosage was recoded to the published effect allele. |
| Strand complement | The complemented target alleles matched the scoring alleles unambiguously; the strand was complemented and effect-allele dosage retained. |
| Palindromic resolved | For A/T or C/G pairs, orientation was retained only when \|EAF_target − EAF_EAS\| ≤0.10 and \|EAF_target − (1 − EAF_EAS)\| >0.10. |
| Palindromic unresolved | The frequency rule did not distinguish the two orientations; the variant was excluded. |
| Incompatible | Alleles could not be reconciled by direct match, swap, or unambiguous complement; the variant was excluded. |
| Unavailable/unmatched | The score variant was absent after imputation or could not be matched by GRCh37 coordinate and allele pair; the variant was excluded. |

Effect-allele frequency was calculated from imputed dosages in the target center. EAS reference frequencies were calculated from the fixed 1000 Genomes Phase 3 EAS panel. The original and harmonized alleles, orientation decision, frequency difference, and exclusion reason were retained in the center-specific harmonization audit file.

# 11. Duplicated positions and the fixed cross-center scoring set

After allele compatibility had been evaluated, multiple eligible scoring rows at the same GRCh37 chromosome-position key were classified as duplicated genomic positions. All rows at an unresolved duplicated position were excluded; no variant was selected on the basis of effect size. This rule prevented one dosage record from receiving more than one weight.

A fixed scoring set was then defined as the intersection of variants that were available, passed INFO and MAF thresholds, and passed harmonization in all three centers. The intersection was based on a normalized chromosome:position:reference-allele:alternate-allele identifier. The identical 4,982,614-variant set was used for every participant at every center. Center-specific extra variants were not scored.

| **Processing stage** | **Variants, n** | **Percentage of published score** |
| --- | --- | --- |
| Published PGS000013 variants | 6,630,150 | 100.0% |
| Available after imputation in all three centers | 6,124,685 | 92.4% |
| INFO ≥0.80 | 5,571,826 | 84.0% |
| Additionally MAF ≥1% | 5,022,947 | 75.8% |
| Excluded: incompatible alleles | 18,764 | 0.28% |
| Excluded: duplicated genomic positions | 2,417 | 0.04% |
| Excluded: unresolved palindromic variants | 19,152 | 0.29% |
| Final common scoring set | 4,982,614 | 75.2% |
| Effect-weighted coverage | — | 82.8% |

**Note:** Exclusions were applied sequentially and were mutually exclusive within the indicated processing stage. Full center-specific counts are reported in Supplementary Table S2 of the manuscript.

# 12. Raw PRS calculation

For participant i, the raw score was calculated as PRS_i = Σ_j(β_j × G_ij), where β_j was the published log-odds weight and G_ij was the imputed dosage of the published effect allele from 0 to 2. No weighting, calibration, or ancestry-specific coefficient fitting was performed in the target cohort. Before scoring, each retained VCF record was assigned a unique chromosome:position:REF:ALT identifier, and the score file contained that identifier, effect allele, and published weight.

| **Operation** | **Command** |
| --- | --- |
| Prepare common score file | python 05_harmonize_weights.py --pgs PGS000013_hmPOS_GRCh37.txt.gz --center-a wuxi.harmonized.tsv.gz --center-b hainan.harmonized.tsv.gz --center-c jiangnan.harmonized.tsv.gz --out gps_cad_common.score --seed 20260726 |
| Score one center | plink --vcf ${CENTER}_common.imputed.vcf.gz dosage=DS --double-id --score gps_cad_common.score 1 2 3 header sum --out ${CENTER}_gps_cad |
| Verify variant count | python 06_verify_score.py --profile ${CENTER}_gps_cad.profile --expected-variants 4982614 --out ${CENTER}_gps_cad.audit.tsv |

**Note:** The harmonized score file columns were variant identifier, effect allele, and published weight. PLINK's sum modifier returned the weighted sum rather than the mean contribution per scored allele.

Unweighted coverage was 4,982,614 / 6,630,150 = 75.2%. Effect-weighted coverage was Σ|β_j| over retained variants divided by Σ|β_j| over all published variants and was 82.8%.

# 13. Genetic principal components

## 13.1 Internal–external validation cycles

PC adjustment used quality-controlled, directly genotyped, autosomal variants common to the relevant datasets. Within each leave-one-center-out cycle, LD pruning and PCA were performed only in the two-center development dataset. The held-out center contributed neither allele frequencies nor eigenvectors. LD pruning used a 200-variant window, 50-variant step, and r² threshold of 0.10. Ten PCs were retained.

| **Operation** | **Command** |
| --- | --- |
| LD pruning in development data | plink --bfile ${CYCLE}_development --indep-pairwise 200 50 0.10 --out ${CYCLE}_prune |
| Development PCA and loadings | plink --bfile ${CYCLE}_development --extract ${CYCLE}_prune.prune.in --pca 10 header tabs var-wts --out ${CYCLE}_pca |
| Project held-out center | Rscript 07_project_pcs.R --loadings ${CYCLE}_pca.eigenvec.var --development-frequencies ${CYCLE}_development.frq --held-out ${CYCLE}_heldout_dosages.tsv.gz --n-pc 10 --out ${CYCLE}_heldout_pcs.tsv |

Held-out genotypes were aligned to the development-data effect allele and standardized using the development-data allele frequency. For variant l and PC k, the projected score used the development loading multiplied by (G_il − 2p_l)/√[2p_l(1 − p_l)], summed over the retained LD-pruned variants. Variants absent or ambiguously aligned in the held-out center were omitted from both the held-out projection and the corresponding development loading set before projection.

## 13.2 Reference-population visualization

For Supplementary Figure S2, the 1000 Genomes Phase 3 EAS samples were restricted to quality-controlled, LD-pruned autosomal variants shared with the study data. PCA loadings and allele-frequency scaling parameters were estimated in the 504 EAS reference samples, and participants from all three centers were projected onto those fixed loadings. This reference projection was used only for ancestry visualization and was separate from the development-only PCs used in model validation.

# 14. PC residualization and standardization of the PRS

Within each outer validation cycle, the raw PRS was regressed on PC1–PC10 in the two-center development dataset:

Raw PRS_i = α_dev + γ_1,dev PC1_i + … + γ_10,dev PC10_i + ε_i.

For development participants, the PC-adjusted PRS was the residual ε_i. For a held-out participant h, the adjusted value was raw PRS_h − [α_dev + Σ_k γ_k,dev PCk_h]. The held-out value was then standardized as Z_h = (adjusted PRS_h − μ_dev)/SD_dev, where μ_dev and SD_dev were the mean and SD of the development residuals. The regression coefficients, μ_dev, and SD_dev were applied unchanged to the held-out center. PC1–PC10 were therefore not entered separately as predictors in the XGBoost models.

| **Operation** | **Command** |
| --- | --- |
| Cycle-specific adjustment | Rscript 08_adjust_prs.R --development ${CYCLE}_development_prs_pcs.tsv --held-out ${CYCLE}_heldout_prs_pcs.tsv --pcs PC1-PC10 --out-development ${CYCLE}_development_prs_z.tsv --out-held-out ${CYCLE}_heldout_prs_z.tsv |
| Full-cohort final-model adjustment | Rscript 08_adjust_prs.R --development full_cohort_prs_pcs.tsv --pcs PC1-PC10 --out-development full_cohort_prs_z.tsv |

After completion of internal–external validation, the same regression and standardization procedure was repeated in the complete cohort for final-model fitting, SHAP analyses, and PRS association analyses. This full-cohort parameter set was not used to estimate validation performance. Separately, a descriptive raw PRS Z-score was calculated from the complete-cohort raw-score mean and SD without PC residualization; it was used only for descriptive summaries.

# 15. Quality-assurance outputs

The following non-identifying audit outputs were retained for independent verification:

- software-version logs, command lines, reference-panel checksums, scoring-file checksum, and the fixed random seed;
- sample and directly genotyped variant counts after each array-level quality-control step;
- per-center and per-chromosome phased and imputed sample/variant counts;
- INFO and MAF distributions and sequential exclusion counts;
- allele-harmonization status, original and final alleles, frequency checks, and exclusion reason for each score row;
- the final 4,982,614-variant common-set identifier list and unweighted/effect-weighted coverage;
- participant-level raw PRS, cycle-specific PC-adjusted standardized PRS, and full-cohort PC-adjusted standardized PRS in the protected analysis environment; and
- development coefficients, development residual mean and SD, held-out PC projections, and final distribution checks for each validation cycle.

Patient names, medical-record numbers, addresses, contact details, and other direct identifiers were not included in genetic-processing files or supplementary materials. Study identifiers were used within the approved analytic environment. Publicly distributed materials contain only code, data dictionaries, aggregate variant counts, and non-identifying workflow documentation.

# 16. Abbreviations

BCF, binary variant call format; EAF, effect-allele frequency; EAS, East Asian; INFO, imputation quality score; LD, linkage disequilibrium; MAF, minor allele frequency; PC, principal component; PCA, principal component analysis; PGS, polygenic score; PRS, polygenic risk score; VCF, variant call format.

# 17. References

1. Khera AV, Chaffin M, Aragam KG, et al. Genome-wide polygenic scores for common diseases identify individuals with risk equivalent to monogenic mutations. Nat Genet. 2018;50(9):1219-1224. doi:10.1038/s41588-018-0183-z

2. Lambert SA, Gil L, Jupp S, et al. The Polygenic Score Catalog as an open database for reproducibility and systematic evaluation. Nat Genet. 2021;53(4):420-425. doi:10.1038/s41588-021-00783-5

3. 1000 Genomes Project Consortium. A global reference for human genetic variation. Nature. 2015;526(7571):68-74. doi:10.1038/nature15393

4. Delaneau O, Zagury JF, Robinson MR, Marchini JL, Dermitzakis ET. Accurate, scalable and integrative haplotype estimation. Nat Commun. 2019;10(1):5436. doi:10.1038/s41467-019-13225-y

5. Das S, Forer L, Schönherr S, et al. Next-generation genotype imputation service and methods. Nat Genet. 2016;48(10):1284-1287. doi:10.1038/ng.3656

6. Choi SW, Mak TSH, O’Reilly PF. Tutorial: a guide to performing polygenic risk score analyses. Nat Protoc. 2020;15(9):2759-2772. doi:10.1038/s41596-020-0353-1

7. Wand H, Lambert SA, Tamburro C, et al. Improving reporting standards for polygenic scores in risk prediction studies. Nature. 2021;591(7849):211-219. doi:10.1038/s41586-021-03243-6
