# Supplementary material for: Development and internal–external validation of an interpretable machine learning model integrating clinical and polygenic predictors for coronary artery disease: a multicenter retrospective cohort study
Source: Front Cardiovasc Med. 2026 Sep 24;13:1936899. doi: 10.3389/fcvm.2026.1936899 (PMC13645527; doi:10.3389/fcvm.2026.1936899)
Supplement: Supplementary file 4 [file Supplementaryfile3.docx]

**Supplementary File S3**

# 1. Scope

This reproducibility package contains the final analysis workflow for the three-center retrospective cohort study of 5-year incident CAD risk. It covers cohort derivation, endpoint construction, multiple imputation, validation-cycle-specific PRS processing, clinical feature selection, nested XGBoost development, cause-specific Cox benchmarking, competing-risk performance assessment, Fine–Gray association analyses, and prespecified sensitivity analyses.

No patient-level data, direct identifiers, institutional file-system paths, access credentials, or GPS_CAD weight file are distributed with this supplement. The code operates on de-identified files prepared in the approved analysis environment.

# 2. Required directory structure

```
project_root/
  code/                         # converted scripts 00-07
  input/screening_data.csv
  input/adjudication_data.csv
  input/prs/gpscad_coverage.csv
  input/prs/prs_holdout_wuxi.csv
  input/prs/prs_holdout_hainan.csv
  input/prs/prs_holdout_jiangnan.csv
  input/prs/prs_full_cohort.csv
  intermediate/                 # created automatically
  models/                       # created automatically
  output/                       # created automatically
```

# 3. Software and fixed settings

| **Software/package** | **Version** | **Use** |
| --- | --- | --- |
| R | 4.5.1 | Statistical workflow |
| Python | 3.11.9 | XGBoost workflow |
| mice | 3.19.0 | Multiple imputation |
| glmnet | 4.1-10 | Cause-specific Cox LASSO |
| cmprsk | 2.2-12 | Fine–Gray and Gray tests |
| riskRegression | 2026.02.13 | Competing-risk prediction performance |
| pmsampsize | 1.1.3 | Riley sample-size calculation |
| xgboost | 2.1.4 | Cause-specific boosted Cox models |
| scikit-learn | 1.6.1 | Stratified inner folds |
| PLINK / SHAPEIT4 / Minimac4 | 1.90b6.21 / 4.2.2 / 4.1.6 | Genetic preprocessing documented in Supplementary File S2 |

**Random seed:** 20260726. The seed is offset deterministically for validation cycles, imputations, feature-selection folds, tuning searches, and bootstrap analyses.

# 4. Principal outputs

| **Script** | **Outputs** |
| --- | --- |
| 01 | cohort_flow_counts.csv; endpoint_count_audit.csv; adjudication_agreement.csv; cohort_derived.rds |
| 02 | Cycle-specific and full-cohort imputed datasets; mice objects; imputation_audit.csv |
| 03 | Cycle-specific/full-cohort adjusted PRS files and parameter objects; prs_distribution_audit.csv |
| 04 | LASSO objects; lasso_selected_features.csv; lasso_penalty_summary.csv; selection frequencies |
| 05 | Out-of-center primary/hard-endpoint predictions; tuning records; fitted final integrated models |
| 06 | Performance, calibration, bootstrap intervals, model differences, DCA, China-PAR, and sensitivity outputs |
| 07 | Fine–Gray estimates, interaction estimates, proportionality diagnostics, Gray tests, and Riley outputs |

# 5. Frozen audit targets

| **Audit item** | **Expected value** |
| --- | --- |
| Screened / included | 2,614 / 2,067 |
| Included by center | Wuxi 812; Hainan 604; Jiangnan 651 |
| Primary endpoint / competing death, complete follow-up | 215 / 78 |
| Primary endpoint / competing death within 5 years | 163 / 57 |
| Censored before 5 years / event-free and observed at 5 years | 487 / 1,360 |
| Hard coronary endpoint, complete follow-up / within 5 years | 141 / 103 |
| Published / final common GPS_CAD variants | 6,630,150 / 4,982,614 |
| Unweighted / effect-weighted GPS_CAD coverage | 75.2% / 82.8% |

# 6. Data-handling notes

- Dates use ISO format YYYY-MM-DD. Missing values are blank or NA.
- Binary fields use 0 = no and 1 = yes. Center values are Wuxi, Hainan, or Jiangnan.
- The outer-cycle PRS files contain development-derived PC coordinates for that cycle; the full-cohort file contains the corresponding full-cohort coordinates.
- True outcome and follow-up information from each held-out center is masked during imputation-model estimation and restored only for validation.
- The China-PAR risk is supplied as a verified 10-year probability in the eligible complete-case subset and is used only as a ranking score for 5-year discrimination.
- GPS_CAD weights are not redistributed. Retrieve PGS000013 from the PGS Catalog under its stated terms and verify the scoring-file checksum recorded in the protected analysis environment.

# 7. External resources

GPS_CAD scoring weights: PGS Catalog accession PGS000013, https://www.pgscatalog.org/score/PGS000013/. Reporting framework: Collins GS, et al. TRIPOD+AI statement. BMJ. 2024;385:e078378. doi:10.1136/bmj-2023-078378.

# 8. Input data dictionary

The following fields define the de-identified input interface used by scripts 01–07. Derived outputs are not included in this dictionary.

| **Input file** | **Variable** | **Type** | **Coding/unit** | **Definition** |
| --- | --- | --- | --- | --- |
| screening_data.csv | participant_id | String | Unique de-identified value | Stable participant key; no medical-record number |
| screening_data.csv | center | Categorical | Wuxi; Hainan; Jiangnan | Participating center |
| screening_data.csv | baseline_date | Date | YYYY-MM-DD | First eligible clinical evaluation |
| screening_data.csv | last_contact_date | Date | YYYY-MM-DD | Last valid clinical contact |
| screening_data.csv | previous_cad | Integer | 0 = no; 1 = yes | Documented CAD before baseline |
| screening_data.csv | qualifying_risk_factor | Integer | 0 = no; 1 = yes | At least one prespecified eligibility risk factor |
| screening_data.csv | genotype_qc_pass | Integer | 0 = no; 1 = yes | Individual genotype data passed quality control and allowed PRS calculation |
| screening_data.csv | baseline_determinable | Integer | 0 = no; 1 = yes | Baseline date could be established |
| screening_data.csv | followup_determinable | Integer | 0 = no; 1 = yes | Follow-up time could be established |
| screening_data.csv | life_limiting_condition | Integer | 0 = no; 1 = yes | Condition associated with expected survival <1 year |
| screening_data.csv | age | Numeric | years | Age at baseline |
| screening_data.csv | male_sex | Integer | 0 = female; 1 = male | Recorded sex |
| screening_data.csv | bmi | Numeric | kg/m² | Body mass index |
| screening_data.csv | sbp | Numeric | mmHg | Baseline seated systolic blood pressure |
| screening_data.csv | dbp | Numeric | mmHg | Baseline seated diastolic blood pressure |
| screening_data.csv | heart_rate | Numeric | beats/min | Resting heart rate |
| screening_data.csv | current_smoking | Integer | 0 = former/never; 1 = current | Smoking status used in modeling |
| screening_data.csv | current_alcohol | Integer | 0 = no; 1 = yes | Current alcohol use |
| screening_data.csv | family_history_premature_ascvd | Integer | 0 = no; 1 = yes | Premature ASCVD in a first-degree relative |
| screening_data.csv | hypertension | Integer | 0 = no; 1 = yes | Prespecified hypertension definition |
| screening_data.csv | t2dm | Integer | 0 = no; 1 = yes | Type 2 diabetes mellitus |
| screening_data.csv | dyslipidemia | Integer | 0 = no; 1 = yes | Documented dyslipidemia, abnormal lipids, or therapy |
| screening_data.csv | ckd | Integer | 0 = no; 1 = yes | Documented CKD or eGFR <60 mL/min/1.73 m² |
| screening_data.csv | total_cholesterol | Numeric | mmol/L | Fasting measurement within 7 days |
| screening_data.csv | ldl_c | Numeric | mmol/L | LDL cholesterol within 7 days |
| screening_data.csv | hdl_c | Numeric | mmol/L | HDL cholesterol within 7 days |
| screening_data.csv | triglycerides | Numeric | mmol/L | Fasting triglycerides within 7 days |
| screening_data.csv | fasting_glucose | Numeric | mmol/L | Fasting plasma glucose within 7 days |
| screening_data.csv | hba1c | Numeric | % | Hemoglobin A1c within 7 days |
| screening_data.csv | creatinine | Numeric | µmol/L | Serum creatinine within 7 days |
| screening_data.csv | egfr | Numeric | mL/min/1.73 m² | 2021 CKD-EPI creatinine eGFR; passively recalculated |
| screening_data.csv | uric_acid | Numeric | µmol/L | Uric acid within 7 days |
| screening_data.csv | hs_crp | Numeric | mg/L | High-sensitivity C-reactive protein within 7 days |
| screening_data.csv | wbc | Numeric | ×10⁹/L | White blood cell count within 7 days |
| screening_data.csv | hemoglobin | Numeric | g/L | Hemoglobin within 7 days |
| screening_data.csv | platelet | Numeric | ×10⁹/L | Platelet count within 7 days |
| screening_data.csv | alt | Numeric | U/L | Alanine aminotransferase within 7 days |
| screening_data.csv | ast | Numeric | U/L | Aspartate aminotransferase within 7 days |
| screening_data.csv | statin_use | Integer | 0 = no; 1 = yes | Active statin prescription at baseline |
| screening_data.csv | antiplatelet_use | Integer | 0 = no; 1 = yes | Active antiplatelet prescription at baseline |
| screening_data.csv | antihypertensive_use | Integer | 0 = no; 1 = yes | Active antihypertensive prescription at baseline |
| screening_data.csv | raw_prs | Numeric | Weighted dosage sum | Raw GPS_CAD before PC adjustment |
| screening_data.csv | mi_date | Date | YYYY-MM-DD or missing | Adjudicated non-fatal MI date |
| screening_data.csv | revascularization_date | Date | YYYY-MM-DD or missing | Adjudicated ischemia-driven PCI or CABG date |
| screening_data.csv | coronary_death_date | Date | YYYY-MM-DD or missing | Adjudicated coronary death date |
| screening_data.csv | noncoronary_death_date | Date | YYYY-MM-DD or missing | Death date not adjudicated as coronary death |
| screening_data.csv | waist_cm | Numeric | cm or missing | China-PAR-only waist circumference |
| screening_data.csv | geographic_region | Categorical | China-PAR region code or missing | China-PAR-only geographic region |
| screening_data.csv | urbanization | Categorical | Urban; rural; or missing | China-PAR-only residential classification |
| screening_data.csv | china_par_risk_10y | Numeric | Probability from 0 to 1 or missing | Verified original China-PAR 10-year ASCVD risk |
| adjudication_data.csv | adjudication_id | String | Unique de-identified record key | Potential-event record identifier |
| adjudication_data.csv | participant_id | String | Links to screening_data.csv | De-identified participant key |
| adjudication_data.csv | center | Categorical | Wuxi; Hainan; Jiangnan | Center used for stratified bootstrap |
| adjudication_data.csv | adjudicator1 | Categorical | Finalized adjudication category | First blinded cardiologist classification |
| adjudication_data.csv | adjudicator2 | Categorical | Same levels as adjudicator1 | Second blinded cardiologist classification |
| adjudication_data.csv | final_classification | Categorical | Arbitrated category | Final classification after arbitration when required |
| prs_cycle_files | participant_id | String | Unique de-identified value | Participant key |
| prs_cycle_files | raw_prs | Numeric | Weighted dosage sum | Raw GPS_CAD from the fixed 4,982,614-variant scoring set |
| prs_cycle_files | pc1 | Numeric | Cycle-specific PC score | Genetic principal component 1; development-derived or projected as appropriate |
| prs_cycle_files | pc2 | Numeric | Cycle-specific PC score | Genetic principal component 2; development-derived or projected as appropriate |
| prs_cycle_files | pc3 | Numeric | Cycle-specific PC score | Genetic principal component 3; development-derived or projected as appropriate |
| prs_cycle_files | pc4 | Numeric | Cycle-specific PC score | Genetic principal component 4; development-derived or projected as appropriate |
| prs_cycle_files | pc5 | Numeric | Cycle-specific PC score | Genetic principal component 5; development-derived or projected as appropriate |
| prs_cycle_files | pc6 | Numeric | Cycle-specific PC score | Genetic principal component 6; development-derived or projected as appropriate |
| prs_cycle_files | pc7 | Numeric | Cycle-specific PC score | Genetic principal component 7; development-derived or projected as appropriate |
| prs_cycle_files | pc8 | Numeric | Cycle-specific PC score | Genetic principal component 8; development-derived or projected as appropriate |
| prs_cycle_files | pc9 | Numeric | Cycle-specific PC score | Genetic principal component 9; development-derived or projected as appropriate |
| prs_cycle_files | pc10 | Numeric | Cycle-specific PC score | Genetic principal component 10; development-derived or projected as appropriate |
| gpscad_coverage.csv | processing_stage | String | Prespecified stage label | published, common_available, info_pass, maf_pass, or final_common |
| gpscad_coverage.csv | common_set_n | Integer | Variant count | Cross-center common-set count at the specified stage |
| gpscad_coverage.csv | wuxi_n | Integer | Variant count | Center-specific count before final common-set restriction |
| gpscad_coverage.csv | hainan_n | Integer | Variant count | Center-specific count before final common-set restriction |
| gpscad_coverage.csv | jiangnan_n | Integer | Variant count | Center-specific count before final common-set restriction |

**00_config.R**

```
# Supplementary File S3: reproducibility configuration
# Study: three-center CAD prediction model with GPS_CAD

options(stringsAsFactors = FALSE, warn = 1)
set.seed(20260726)

SEED <- 20260726L
ADMIN_END <- as.Date("2024-12-31")
HORIZON_PRIMARY <- 5
HORIZON_SENSITIVITY <- 3
N_IMPUTATIONS <- 20L
N_OUTER_CYCLES <- 3L
N_LASSO_FOLDS <- 10L
N_TUNING_FOLDS <- 5L
N_HYPERPARAMETER_DRAWS <- 100L
N_BOOTSTRAP <- 1000L

PROJECT_ROOT <- normalizePath(".", mustWork = TRUE)
INPUT_DIR <- file.path(PROJECT_ROOT, "input")
INTERMEDIATE_DIR <- file.path(PROJECT_ROOT, "intermediate")
OUTPUT_DIR <- file.path(PROJECT_ROOT, "output")
MODEL_DIR <- file.path(PROJECT_ROOT, "models")

for (x in c(INTERMEDIATE_DIR, OUTPUT_DIR, MODEL_DIR)) {
  if (!dir.exists(x)) dir.create(x, recursive = TRUE)
}

CENTER_LEVELS <- c("Wuxi", "Hainan", "Jiangnan")
OUTER_CYCLES <- setNames(CENTER_LEVELS, paste0("holdout_", tolower(CENTER_LEVELS)))

CLINICAL_VARS <- c(
  "age", "male_sex", "bmi", "sbp", "dbp", "heart_rate",
  "current_smoking", "current_alcohol", "family_history_premature_ascvd",
  "hypertension", "t2dm", "dyslipidemia", "ckd",
  "total_cholesterol", "ldl_c", "hdl_c", "triglycerides",
  "fasting_glucose", "hba1c", "creatinine", "egfr", "uric_acid",
  "hs_crp", "wbc", "hemoglobin", "platelet", "alt", "ast",
  "statin_use", "antiplatelet_use", "antihypertensive_use"
)

BINARY_VARS <- c(
  "male_sex", "current_smoking", "current_alcohol",
  "family_history_premature_ascvd", "hypertension", "t2dm",
  "dyslipidemia", "ckd", "statin_use", "antiplatelet_use",
  "antihypertensive_use"
)

CONTINUOUS_VARS <- setdiff(CLINICAL_VARS, BINARY_VARS)
PC_VARS <- paste0("pc", 1:10)

LOG_SMD_VARS <- c("triglycerides", "hs_crp", "alt", "ast")

ASSOCIATION_COVARIATES <- c(
  "age", "male_sex", "bmi", "sbp", "current_smoking", "t2dm",
  "ldl_c", "hdl_c", "egfr", "statin_use", "center", PC_VARS
)

INTERACTION_COVARIATES <- c(
  "age", "male_sex", "bmi", "sbp", "current_smoking", "t2dm",
  "hdl_c", "egfr", "statin_use", "center", PC_VARS
)

EXPECTED_COUNTS <- list(
  screened = 2614L,
  included = 2067L,
  included_by_center = c(Wuxi = 812L, Hainan = 604L, Jiangnan = 651L),
  primary_events = 215L,
  competing_deaths = 78L,
  primary_events_5y = 163L,
  competing_deaths_5y = 57L,
  censored_before_5y = 487L,
  event_free_at_5y = 1360L,
  hard_events = 141L,
  hard_events_5y = 103L,
  endpoint_components = c(mi = 103L, revascularization = 85L, coronary_death = 27L)
)

EXPECTED_PRS <- list(
  published = 6630150L,
  common_available = 6124685L,
  info_pass = 5571826L,
  maf_pass = 5022947L,
  final_common = 4982614L,
  unweighted_coverage = 0.752,
  weighted_coverage = 0.828
)

PACKAGE_VERSIONS <- c(
  mice = "3.19.0",
  glmnet = "4.1-10",
  cmprsk = "2.2-12",
  riskRegression = "2026.02.13",
  pmsampsize = "1.1.3"
)

assert_columns <- function(data, required, object_name = deparse(substitute(data))) {
  missing <- setdiff(required, names(data))
  if (length(missing)) {
    stop(object_name, " is missing required columns: ", paste(missing, collapse = ", "))
  }
  invisible(TRUE)
}

assert_binary <- function(x, name) {
  values <- unique(stats::na.omit(x))
  if (!all(values %in% c(0, 1))) stop(name, " must contain only 0, 1, or NA")
  invisible(TRUE)
}

check_count <- function(observed, expected, label) {
  if (!identical(as.integer(observed), as.integer(expected))) {
    stop(label, ": observed ", observed, "; expected ", expected)
  }
  invisible(TRUE)
}

write_audit <- function(x, filename) {
  utils::write.csv(x, file.path(OUTPUT_DIR, filename), row.names = FALSE, na = "")
}

save_object <- function(x, filename) {
  saveRDS(x, file.path(INTERMEDIATE_DIR, filename), compress = "xz")
}

read_object <- function(filename) {
  readRDS(file.path(INTERMEDIATE_DIR, filename))
}

years_between <- function(start, end) as.numeric(end - start) / 365.25

event_by_horizon <- function(time, status, horizon, event_code = 1L) {
  as.integer(status == event_code & time <= horizon)
}

verify_versions <- function(strict = FALSE) {
  observed <- vapply(names(PACKAGE_VERSIONS), function(pkg) {
    if (!requireNamespace(pkg, quietly = TRUE)) return(NA_character_)
    as.character(utils::packageVersion(pkg))
  }, character(1))
  out <- data.frame(package = names(PACKAGE_VERSIONS),
                    expected = unname(PACKAGE_VERSIONS),
                    observed = unname(observed),
                    stringsAsFactors = FALSE)
  out$match <- out$expected == out$observed
  if (strict && any(!out$match | is.na(out$match))) {
    stop("Software-version check failed; see output/software_versions.csv")
  }
  write_audit(out, "software_versions.csv")
  invisible(out)
}

session_manifest <- function() {
  sink(file.path(OUTPUT_DIR, "sessionInfo.txt"))
  on.exit(sink(), add = TRUE)
  print(sessionInfo())
}

verify_versions(strict = FALSE)
```

**01_cohort_and_endpoints.R**

```
# Cohort derivation, competing-risk outcomes, and endpoint-adjudication audit

source("code/00_config.R")

screening_file <- file.path(INPUT_DIR, "screening_data.csv")
adjudication_file <- file.path(INPUT_DIR, "adjudication_data.csv")

screen <- utils::read.csv(screening_file, check.names = FALSE, na.strings = c("", "NA"))

required <- c(
  "participant_id", "center", "baseline_date", "last_contact_date",
  "previous_cad", "qualifying_risk_factor", "genotype_qc_pass",
  "baseline_determinable", "followup_determinable", "life_limiting_condition",
  "mi_date", "revascularization_date", "coronary_death_date",
  "noncoronary_death_date", CLINICAL_VARS, "raw_prs",
  "waist_cm", "geographic_region", "urbanization", "china_par_risk_10y"
)
assert_columns(screen, required, "screening_data.csv")
check_count(nrow(screen), EXPECTED_COUNTS$screened, "Screened participants")
if (anyDuplicated(screen$participant_id)) stop("participant_id must be unique")

screen$center <- factor(screen$center, levels = CENTER_LEVELS)
if (any(is.na(screen$center))) stop("Unknown center code")

date_vars <- c(
  "baseline_date", "last_contact_date", "mi_date", "revascularization_date",
  "coronary_death_date", "noncoronary_death_date"
)
screen[date_vars] <- lapply(screen[date_vars], as.Date)

flag_vars <- c(
  "previous_cad", "qualifying_risk_factor", "genotype_qc_pass",
  "baseline_determinable", "followup_determinable", "life_limiting_condition",
  BINARY_VARS
)
for (v in intersect(flag_vars, names(screen))) assert_binary(screen[[v]], v)

# Apply mutually exclusive exclusions in the prespecified order.
screen$exclusion_reason <- NA_character_
screen$exclusion_reason[screen$previous_cad == 1] <- "Previous CAD"
idx <- is.na(screen$exclusion_reason) & screen$qualifying_risk_factor != 1
screen$exclusion_reason[idx] <- "No qualifying cardiovascular risk factor"
idx <- is.na(screen$exclusion_reason) & screen$genotype_qc_pass != 1
screen$exclusion_reason[idx] <- "Genotype QC failure or PRS unavailable"
idx <- is.na(screen$exclusion_reason) &
  (screen$baseline_determinable != 1 | screen$followup_determinable != 1 |
     is.na(screen$baseline_date) | is.na(screen$last_contact_date))
screen$exclusion_reason[idx] <- "Baseline or follow-up time indeterminate"
idx <- is.na(screen$exclusion_reason) & screen$life_limiting_condition == 1
screen$exclusion_reason[idx] <- "Life-limiting condition"

expected_exclusions <- c(
  "Previous CAD" = 258L,
  "No qualifying cardiovascular risk factor" = 114L,
  "Genotype QC failure or PRS unavailable" = 91L,
  "Baseline or follow-up time indeterminate" = 47L,
  "Life-limiting condition" = 37L
)
observed_exclusions <- table(factor(screen$exclusion_reason, levels = names(expected_exclusions)))
if (!identical(as.integer(observed_exclusions), as.integer(expected_exclusions))) {
  stop("Screening exclusions do not match the frozen audit counts")
}

flow_counts <- rbind(
  data.frame(stage = "Screened", reason = "", n = nrow(screen)),
  data.frame(stage = "Excluded", reason = names(observed_exclusions),
             n = as.integer(observed_exclusions)),
  data.frame(stage = "Included", reason = "", n = sum(is.na(screen$exclusion_reason)))
)
write_audit(flow_counts, "cohort_flow_counts.csv")

cohort <- screen[is.na(screen$exclusion_reason), , drop = FALSE]
check_count(nrow(cohort), EXPECTED_COUNTS$included, "Included participants")
center_counts <- table(cohort$center)
if (!identical(as.integer(center_counts), as.integer(EXPECTED_COUNTS$included_by_center))) {
  stop("Included center counts do not match the frozen audit counts")
}

if (any(cohort$last_contact_date < cohort$baseline_date, na.rm = TRUE)) {
  stop("last_contact_date precedes baseline_date")
}

row_min_date <- function(...) {
  values <- lapply(list(...), as.numeric)
  ans <- do.call(pmin, c(values, na.rm = TRUE))
  ans[is.infinite(ans)] <- NA_real_
  as.Date(ans, origin = "1970-01-01")
}

# Primary composite: coronary death > non-fatal MI > revascularization on the same day.
cohort$primary_candidate_date <- row_min_date(
  cohort$coronary_death_date, cohort$mi_date, cohort$revascularization_date
)
cohort$primary_component <- NA_character_
cohort$primary_component[
  !is.na(cohort$primary_candidate_date) &
    cohort$primary_candidate_date == cohort$coronary_death_date
] <- "coronary_death"
cohort$primary_component[
  is.na(cohort$primary_component) & !is.na(cohort$primary_candidate_date) &
    cohort$primary_candidate_date == cohort$mi_date
] <- "mi"
cohort$primary_component[
  is.na(cohort$primary_component) & !is.na(cohort$primary_candidate_date) &
    cohort$primary_candidate_date == cohort$revascularization_date
] <- "revascularization"

cohort$censor_date <- pmin(cohort$last_contact_date, ADMIN_END, na.rm = TRUE)
primary_first <- !is.na(cohort$primary_candidate_date) &
  cohort$primary_candidate_date <= cohort$censor_date &
  (is.na(cohort$noncoronary_death_date) |
     cohort$primary_candidate_date <= cohort$noncoronary_death_date)
competing_first <- !primary_first & !is.na(cohort$noncoronary_death_date) &
  cohort$noncoronary_death_date <= cohort$censor_date

cohort$status <- 0L
cohort$status[primary_first] <- 1L
cohort$status[competing_first] <- 2L
cohort$analysis_end_date <- cohort$censor_date
cohort$analysis_end_date[primary_first] <- cohort$primary_candidate_date[primary_first]
cohort$analysis_end_date[competing_first] <- cohort$noncoronary_death_date[competing_first]
cohort$time_years <- years_between(cohort$baseline_date, cohort$analysis_end_date)
if (any(cohort$time_years < 0 | !is.finite(cohort$time_years))) {
  stop("Invalid primary-endpoint follow-up time")
}

# Hard endpoint: non-fatal MI or coronary death; follow-up continues after isolated revascularization.
cohort$hard_candidate_date <- row_min_date(cohort$coronary_death_date, cohort$mi_date)
hard_first <- !is.na(cohort$hard_candidate_date) &
  cohort$hard_candidate_date <= cohort$censor_date &
  (is.na(cohort$noncoronary_death_date) |
     cohort$hard_candidate_date <= cohort$noncoronary_death_date)
hard_competing_first <- !hard_first & !is.na(cohort$noncoronary_death_date) &
  cohort$noncoronary_death_date <= cohort$censor_date
cohort$hard_status <- 0L
cohort$hard_status[hard_first] <- 1L
cohort$hard_status[hard_competing_first] <- 2L
cohort$hard_end_date <- cohort$censor_date
cohort$hard_end_date[hard_first] <- cohort$hard_candidate_date[hard_first]
cohort$hard_end_date[hard_competing_first] <- cohort$noncoronary_death_date[hard_competing_first]
cohort$hard_time_years <- years_between(cohort$baseline_date, cohort$hard_end_date)

cohort$primary_event_5y <- event_by_horizon(cohort$time_years, cohort$status, 5, 1L)
cohort$competing_event_5y <- event_by_horizon(cohort$time_years, cohort$status, 5, 2L)
cohort$censored_before_5y <- as.integer(cohort$status == 0L & cohort$time_years < 5)
cohort$event_free_at_5y <- as.integer(cohort$time_years >= 5)
cohort$hard_event_5y <- event_by_horizon(cohort$hard_time_years, cohort$hard_status, 5, 1L)

check_count(sum(cohort$status == 1L), EXPECTED_COUNTS$primary_events, "Primary events")
check_count(sum(cohort$status == 2L), EXPECTED_COUNTS$competing_deaths, "Competing deaths")
check_count(sum(cohort$primary_event_5y), EXPECTED_COUNTS$primary_events_5y, "Primary events within 5 years")
check_count(sum(cohort$competing_event_5y), EXPECTED_COUNTS$competing_deaths_5y, "Competing deaths within 5 years")
check_count(sum(cohort$censored_before_5y), EXPECTED_COUNTS$censored_before_5y, "Censored before 5 years")
check_count(sum(cohort$event_free_at_5y), EXPECTED_COUNTS$event_free_at_5y, "Event-free at 5 years")
check_count(sum(cohort$hard_status == 1L), EXPECTED_COUNTS$hard_events, "Hard coronary events")
check_count(sum(cohort$hard_event_5y), EXPECTED_COUNTS$hard_events_5y, "Hard coronary events within 5 years")

components <- table(factor(cohort$primary_component[cohort$status == 1L],
                           levels = names(EXPECTED_COUNTS$endpoint_components)))
if (!identical(as.integer(components), as.integer(EXPECTED_COUNTS$endpoint_components))) {
  stop("Primary endpoint component counts do not match the frozen audit counts")
}

endpoint_audit <- data.frame(
  measure = c(
    "Primary endpoint, complete follow-up", "Competing death, complete follow-up",
    "Primary endpoint within 5 years", "Competing death within 5 years",
    "Censored before 5 years", "Event-free and observed at 5 years",
    "Hard coronary endpoint, complete follow-up", "Hard coronary endpoint within 5 years"
  ),
  n = c(
    sum(cohort$status == 1L), sum(cohort$status == 2L),
    sum(cohort$primary_event_5y), sum(cohort$competing_event_5y),
    sum(cohort$censored_before_5y), sum(cohort$event_free_at_5y),
    sum(cohort$hard_status == 1L), sum(cohort$hard_event_5y)
  )
)
write_audit(endpoint_audit, "endpoint_count_audit.csv")

# Agreement before arbitration.
adj <- utils::read.csv(adjudication_file, check.names = FALSE, na.strings = c("", "NA"))
assert_columns(adj, c("adjudication_id", "participant_id", "center",
                      "adjudicator1", "adjudicator2", "final_classification"),
               "adjudication_data.csv")
if (nrow(adj) != 300L) stop("Expected 300 adjudicated records")

cohen_kappa <- function(a, b) {
  lev <- sort(unique(c(as.character(a), as.character(b))))
  tab <- table(factor(a, levels = lev), factor(b, levels = lev))
  po <- sum(diag(tab)) / sum(tab)
  pe <- sum(rowSums(tab) * colSums(tab)) / sum(tab)^2
  c(agreement = po, kappa = (po - pe) / (1 - pe))
}

agreement <- cohen_kappa(adj$adjudicator1, adj$adjudicator2)
set.seed(SEED + 101L)
boot_kappa <- replicate(2000L, {
  index <- unlist(lapply(split(seq_len(nrow(adj)), adj$center), function(ii) {
    sample(ii, length(ii), replace = TRUE)
  }), use.names = FALSE)
  cohen_kappa(adj$adjudicator1[index], adj$adjudicator2[index])["kappa"]
})
agreement_result <- data.frame(
  records = nrow(adj),
  agreements = sum(adj$adjudicator1 == adj$adjudicator2),
  agreement_percent = 100 * unname(agreement["agreement"]),
  kappa = unname(agreement["kappa"]),
  kappa_ci_low = unname(stats::quantile(boot_kappa, 0.025, na.rm = TRUE)),
  kappa_ci_high = unname(stats::quantile(boot_kappa, 0.975, na.rm = TRUE))
)
write_audit(agreement_result, "adjudication_agreement.csv")

save_object(cohort, "cohort_derived.rds")
session_manifest()
```

**02_imputation.R**

```
# Twenty-fold MICE within each outer validation cycle and in the full cohort

source("code/00_config.R")
if (!requireNamespace("mice", quietly = TRUE)) stop("Package mice is required")
if (!requireNamespace("survival", quietly = TRUE)) stop("Package survival is required")

cohort <- read_object("cohort_derived.rds")

nelson_aalen <- function(time, event) {
  fit <- survival::coxph(survival::Surv(time, event) ~ 1, ties = "breslow")
  bh <- survival::basehaz(fit, centered = FALSE)
  if (!nrow(bh)) return(rep(0, length(time)))
  idx <- findInterval(time, bh$time)
  ifelse(idx == 0L, 0, bh$hazard[pmax(idx, 1L)])
}

egfr_passive <- paste0(
  "~I(142 * pmin((creatinine / 88.4) / ifelse(male_sex == 0, 0.7, 0.9), 1)^",
  "ifelse(male_sex == 0, -0.241, -0.302) * ",
  "pmax((creatinine / 88.4) / ifelse(male_sex == 0, 0.7, 0.9), 1)^(-1.200) * ",
  "0.9938^age * ifelse(male_sex == 0, 1.012, 1))"
)

write_gzip_csv <- function(x, path) {
  con <- gzfile(path, open = "wt")
  on.exit(close(con), add = TRUE)
  utils::write.csv(x, con, row.names = FALSE, na = "")
}

prepare_imputation_data <- function(data, development_rows, heldout_rows = integer()) {
  development <- data[development_rows, , drop = FALSE]
  missing_fraction <- vapply(CLINICAL_VARS, function(v) mean(is.na(development[[v]])), numeric(1))
  included_vars <- names(missing_fraction)[missing_fraction <= 0.20]
  excluded_vars <- names(missing_fraction)[missing_fraction > 0.20]

  cols <- c("participant_id", "center", included_vars, "time_years", "status")
  imp <- data[c(development_rows, heldout_rows), cols, drop = FALSE]
  imp$event_type <- factor(
    c("censored", "cad", "noncoronary_death")[imp$status + 1L],
    levels = c("censored", "cad", "noncoronary_death")
  )
  imp$na_cad <- nelson_aalen(imp$time_years, imp$status == 1L)
  imp$na_noncoronary_death <- nelson_aalen(imp$time_years, imp$status == 2L)

  ignore <- seq_len(nrow(imp)) > length(development_rows)
  if (any(ignore)) {
    # True held-out outcomes are not available to the imputation procedure.
    imp$event_type[ignore] <- NA
    imp$na_cad[ignore] <- NA_real_
    imp$na_noncoronary_death[ignore] <- NA_real_
  }
  imp$time_years <- NULL
  imp$status <- NULL

  for (v in intersect(BINARY_VARS, names(imp))) {
    imp[[v]] <- factor(imp[[v]], levels = c(0, 1))
  }
  imp$center <- factor(imp$center, levels = CENTER_LEVELS)

  list(data = imp, ignore = ignore, included_vars = included_vars,
       excluded_vars = excluded_vars, missing_fraction = missing_fraction)
}

run_mice <- function(prepared, seed) {
  imp <- prepared$data
  ini <- mice::mice(imp, maxit = 0, printFlag = FALSE)
  method <- ini$method
  pred <- ini$predictorMatrix

  method[] <- ""
  method[intersect(prepared$included_vars, CONTINUOUS_VARS)] <- "pmm"
  method[intersect(prepared$included_vars, BINARY_VARS)] <- "logreg"
  if ("egfr" %in% prepared$included_vars) method["egfr"] <- egfr_passive
  method["event_type"] <- "polyreg"
  method[c("na_cad", "na_noncoronary_death")] <- "pmm"

  pred[,] <- 1L
  diag(pred) <- 0L
  pred[, c("participant_id")] <- 0L
  pred[c("participant_id", "center"), ] <- 0L
  pred["egfr", "egfr"] <- 0L

  mice::mice(
    imp,
    m = N_IMPUTATIONS,
    maxit = 20L,
    method = method,
    predictorMatrix = pred,
    ignore = prepared$ignore,
    donors = 5L,
    seed = seed,
    printFlag = FALSE
  )
}

restore_analysis_fields <- function(completed, original) {
  completed <- completed[match(original$participant_id, completed$participant_id), , drop = FALSE]
  if (!identical(as.character(completed$participant_id), as.character(original$participant_id))) {
    stop("Participant order changed during imputation")
  }
  for (v in intersect(BINARY_VARS, names(completed))) {
    completed[[v]] <- as.integer(as.character(completed[[v]]))
  }
  completed$center <- factor(completed$center, levels = CENTER_LEVELS)
  completed$time_years <- original$time_years
  completed$status <- original$status
  completed$hard_time_years <- original$hard_time_years
  completed$hard_status <- original$hard_status
  completed$baseline_date <- original$baseline_date
  completed$last_contact_date <- original$last_contact_date
  completed$raw_prs <- original$raw_prs
  completed
}

audit_rows <- list()

for (cycle_name in names(OUTER_CYCLES)) {
  heldout_center <- unname(OUTER_CYCLES[[cycle_name]])
  dev_idx <- which(cohort$center != heldout_center)
  val_idx <- which(cohort$center == heldout_center)
  ordered_idx <- c(dev_idx, val_idx)
  prepared <- prepare_imputation_data(cohort, dev_idx, val_idx)
  mids <- run_mice(prepared, SEED + match(cycle_name, names(OUTER_CYCLES)))

  cycle_dir <- file.path(INTERMEDIATE_DIR, "imputed", cycle_name)
  if (!dir.exists(cycle_dir)) dir.create(cycle_dir, recursive = TRUE)
  original <- cohort[ordered_idx, , drop = FALSE]

  for (m in seq_len(N_IMPUTATIONS)) {
    completed <- mice::complete(mids, action = m)
    completed <- restore_analysis_fields(completed, original)
    write_gzip_csv(completed, file.path(cycle_dir, sprintf("imp_%02d.csv.gz", m)))
  }
  saveRDS(mids, file.path(cycle_dir, "mice_object.rds"), compress = "xz")
  utils::write.csv(data.frame(variable = names(prepared$missing_fraction),
                              missing_fraction = unname(prepared$missing_fraction),
                              eligible = names(prepared$missing_fraction) %in% prepared$included_vars),
                   file.path(cycle_dir, "missingness_and_eligibility.csv"), row.names = FALSE)
  audit_rows[[cycle_name]] <- data.frame(
    cycle = cycle_name,
    heldout_center = heldout_center,
    candidate_variables = length(CLINICAL_VARS),
    retained_variables = length(prepared$included_vars),
    excluded_over_20_percent = paste(prepared$excluded_vars, collapse = ";"),
    imputations = N_IMPUTATIONS
  )
}

# Full-cohort imputation for final-model fitting and association analyses.
prepared_full <- prepare_imputation_data(cohort, seq_len(nrow(cohort)))
mids_full <- run_mice(prepared_full, SEED + 100L)
full_dir <- file.path(INTERMEDIATE_DIR, "imputed", "full_cohort")
if (!dir.exists(full_dir)) dir.create(full_dir, recursive = TRUE)
for (m in seq_len(N_IMPUTATIONS)) {
  completed <- mice::complete(mids_full, action = m)
  completed <- restore_analysis_fields(completed, cohort)
  write_gzip_csv(completed, file.path(full_dir, sprintf("imp_%02d.csv.gz", m)))
}
saveRDS(mids_full, file.path(full_dir, "mice_object.rds"), compress = "xz")
utils::write.csv(data.frame(variable = names(prepared_full$missing_fraction),
                            missing_fraction = unname(prepared_full$missing_fraction),
                            eligible = names(prepared_full$missing_fraction) %in% prepared_full$included_vars),
                 file.path(full_dir, "missingness_and_eligibility.csv"), row.names = FALSE)

audit_rows[["full_cohort"]] <- data.frame(
  cycle = "full_cohort", heldout_center = "",
  candidate_variables = length(CLINICAL_VARS),
  retained_variables = length(prepared_full$included_vars),
  excluded_over_20_percent = paste(prepared_full$excluded_vars, collapse = ";"),
  imputations = N_IMPUTATIONS
)
write_audit(do.call(rbind, audit_rows), "imputation_audit.csv")
```

**03_prs_processing.R**

```
# GPS_CAD coverage audit and validation-cycle-specific PC adjustment

source("code/00_config.R")
cohort <- read_object("cohort_derived.rds")

prs_input_dir <- file.path(INPUT_DIR, "prs")
coverage_file <- file.path(prs_input_dir, "gpscad_coverage.csv")
coverage <- utils::read.csv(coverage_file, check.names = FALSE)
assert_columns(coverage, c("processing_stage", "common_set_n"), "gpscad_coverage.csv")

expected_coverage <- c(
  published = EXPECTED_PRS$published,
  common_available = EXPECTED_PRS$common_available,
  info_pass = EXPECTED_PRS$info_pass,
  maf_pass = EXPECTED_PRS$maf_pass,
  final_common = EXPECTED_PRS$final_common
)
matched <- match(names(expected_coverage), coverage$processing_stage)
if (anyNA(matched) || !identical(as.integer(coverage$common_set_n[matched]),
                                  as.integer(expected_coverage))) {
  stop("GPS_CAD coverage file does not match the frozen variant audit")
}

read_prs_cycle <- function(cycle_name) {
  path <- file.path(prs_input_dir, paste0("prs_", cycle_name, ".csv"))
  x <- utils::read.csv(path, check.names = FALSE)
  assert_columns(x, c("participant_id", "raw_prs", PC_VARS), basename(path))
  if (anyDuplicated(x$participant_id)) stop("Duplicated participant_id in ", basename(path))
  x
}

adjust_prs <- function(prs_data, heldout_center = NULL) {
  prs_data$center <- cohort$center[match(prs_data$participant_id, cohort$participant_id)]
  if (anyNA(prs_data$center)) stop("PRS file contains an unknown participant_id")
  prs_data$center <- factor(prs_data$center, levels = CENTER_LEVELS)
  development <- if (is.null(heldout_center)) rep(TRUE, nrow(prs_data)) else
    prs_data$center != heldout_center

  formula_pc <- stats::as.formula(paste("raw_prs ~", paste(PC_VARS, collapse = " + ")))
  fit <- stats::lm(formula_pc, data = prs_data[development, , drop = FALSE])
  predicted_pc_component <- stats::predict(fit, newdata = prs_data)
  prs_data$prs_pc_adjusted <- prs_data$raw_prs - predicted_pc_component
  mu <- mean(prs_data$prs_pc_adjusted[development])
  sigma <- stats::sd(prs_data$prs_pc_adjusted[development])
  if (!is.finite(sigma) || sigma <= 0) stop("Invalid development PRS SD")
  prs_data$prs_pc_adjusted_z <- (prs_data$prs_pc_adjusted - mu) / sigma
  prs_data$is_development <- as.integer(development)

  parameters <- list(
    heldout_center = heldout_center,
    coefficients = stats::coef(fit),
    residual_mean = mu,
    residual_sd = sigma
  )
  list(data = prs_data, parameters = parameters)
}

distribution_audit <- list()
for (cycle_name in names(OUTER_CYCLES)) {
  heldout <- unname(OUTER_CYCLES[[cycle_name]])
  result <- adjust_prs(read_prs_cycle(cycle_name), heldout)
  out <- result$data
  utils::write.csv(out, file.path(INTERMEDIATE_DIR, paste0("prs_adjusted_", cycle_name, ".csv")),
                   row.names = FALSE)
  saveRDS(result$parameters,
          file.path(INTERMEDIATE_DIR, paste0("prs_parameters_", cycle_name, ".rds")))
  val <- out[out$center == heldout, , drop = FALSE]
  distribution_audit[[cycle_name]] <- data.frame(
    dataset = cycle_name, center = heldout, n = nrow(val),
    mean = mean(val$prs_pc_adjusted_z), sd = stats::sd(val$prs_pc_adjusted_z)
  )
}

# Full-cohort parameters are used only after internal-external validation.
full_result <- adjust_prs(read_prs_cycle("full_cohort"), heldout_center = NULL)
full_out <- full_result$data
full_out$raw_prs_z_descriptive <- as.numeric(scale(full_out$raw_prs))
utils::write.csv(full_out, file.path(INTERMEDIATE_DIR, "prs_adjusted_full_cohort.csv"),
                 row.names = FALSE)
saveRDS(full_result$parameters, file.path(INTERMEDIATE_DIR, "prs_parameters_full_cohort.rds"))

check_count(nrow(full_out), EXPECTED_COUNTS$included, "Full-cohort PRS records")
if (abs(mean(full_out$prs_pc_adjusted_z)) > 1e-10 ||
    abs(stats::sd(full_out$prs_pc_adjusted_z) - 1) > 1e-10) {
  stop("Full-cohort PC-adjusted PRS was not standardized to mean 0 and SD 1")
}
if (abs(mean(full_out$raw_prs_z_descriptive)) > 1e-10 ||
    abs(stats::sd(full_out$raw_prs_z_descriptive) - 1) > 1e-10) {
  stop("Descriptive raw PRS Z-score was not standardized to mean 0 and SD 1")
}

distribution_audit[["full_cohort"]] <- data.frame(
  dataset = "full_cohort", center = "All", n = nrow(full_out),
  mean = mean(full_out$prs_pc_adjusted_z), sd = stats::sd(full_out$prs_pc_adjusted_z)
)
write_audit(do.call(rbind, distribution_audit), "prs_distribution_audit.csv")
```

**04_feature_selection.R**

```
# Cause-specific Cox LASSO feature selection within outer development datasets

source("code/00_config.R")
if (!requireNamespace("glmnet", quietly = TRUE)) stop("Package glmnet is required")
if (!requireNamespace("survival", quietly = TRUE)) stop("Package survival is required")

read_imputation <- function(dataset_name, m) {
  path <- file.path(INTERMEDIATE_DIR, "imputed", dataset_name,
                    sprintf("imp_%02d.csv.gz", m))
  utils::read.csv(gzfile(path), check.names = FALSE)
}

make_stratified_folds <- function(data, k, seed) {
  set.seed(seed)
  strata <- interaction(data$center, data$status, drop = TRUE)
  fold <- integer(nrow(data))
  for (s in levels(strata)) {
    ii <- which(strata == s)
    ii <- sample(ii, length(ii), replace = FALSE)
    fold[ii] <- rep(seq_len(k), length.out = length(ii))
  }
  fold
}

stack_imputations <- function(dataset_name, heldout_center = NULL,
                              endpoint = c("primary", "hard")) {
  endpoint <- match.arg(endpoint)
  missing_audit <- utils::read.csv(
    file.path(INTERMEDIATE_DIR, "imputed", dataset_name, "missingness_and_eligibility.csv"),
    check.names = FALSE
  )
  vars <- intersect(CLINICAL_VARS, missing_audit$variable[missing_audit$eligible])
  stacked <- vector("list", N_IMPUTATIONS)
  base_fold <- NULL

  for (m in seq_len(N_IMPUTATIONS)) {
    x <- read_imputation(dataset_name, m)
    if (!is.null(heldout_center)) x <- x[x$center != heldout_center, , drop = FALSE]
    x <- x[order(x$participant_id), , drop = FALSE]
    if (endpoint == "hard") {
      x$time_years <- x$hard_time_years
      x$status <- x$hard_status
    }
    if (m == 1L) {
      base_fold <- make_stratified_folds(x, N_LASSO_FOLDS,
                                         SEED + match(dataset_name, c(names(OUTER_CYCLES), "full_cohort")))
    }
    x$.imp <- m
    x$.fold <- base_fold
    x$.analysis_weight <- 1 / N_IMPUTATIONS
    stacked[[m]] <- x[c("participant_id", "time_years", "status",
                        ".imp", ".fold", ".analysis_weight", vars)]
  }
  list(data = do.call(rbind, stacked), variables = vars)
}

fit_lasso <- function(dataset_name, heldout_center = NULL,
                      endpoint = c("primary", "hard")) {
  endpoint <- match.arg(endpoint)
  stacked <- stack_imputations(dataset_name, heldout_center, endpoint)
  d <- stacked$data
  x <- as.matrix(d[stacked$variables])
  storage.mode(x) <- "double"
  y <- survival::Surv(d$time_years, d$status == 1L)

  set.seed(SEED + 200L + match(dataset_name, c(names(OUTER_CYCLES), "full_cohort")))
  cvfit <- glmnet::cv.glmnet(
    x = x,
    y = y,
    family = "cox",
    alpha = 1,
    weights = d$.analysis_weight,
    foldid = d$.fold,
    type.measure = "deviance",
    grouped = TRUE,
    standardize = TRUE,
    keep = TRUE
  )
  fit <- cvfit$glmnet.fit
  beta_1se <- as.matrix(stats::coef(cvfit, s = "lambda.1se"))[, 1]
  beta_min <- as.matrix(stats::coef(cvfit, s = "lambda.min"))[, 1]
  selected_1se <- names(beta_1se)[beta_1se != 0]
  selected_min <- names(beta_min)[beta_min != 0]
  idx_1se <- which.min(abs(cvfit$lambda - cvfit$lambda.1se))
  idx_min <- which.min(abs(cvfit$lambda - cvfit$lambda.min))

  list(
    cvfit = cvfit,
    selected_1se = selected_1se,
    selected_min = selected_min,
    summary = data.frame(
      endpoint = endpoint,
      dataset = dataset_name,
      heldout_center = if (is.null(heldout_center)) "" else heldout_center,
      lambda_min = cvfit$lambda.min,
      log_lambda_min = log(cvfit$lambda.min),
      deviance_min = cvfit$cvm[idx_min],
      se_min = cvfit$cvsd[idx_min],
      nonzero_min = length(selected_min),
      lambda_1se = cvfit$lambda.1se,
      log_lambda_1se = log(cvfit$lambda.1se),
      deviance_1se = cvfit$cvm[idx_1se],
      se_1se = cvfit$cvsd[idx_1se],
      nonzero_1se = length(selected_1se)
    )
  )
}

selection_rows <- list()
summary_rows <- list()

for (cycle_name in names(OUTER_CYCLES)) {
  heldout <- unname(OUTER_CYCLES[[cycle_name]])
  result <- fit_lasso(cycle_name, heldout, "primary")
  saveRDS(result$cvfit, file.path(MODEL_DIR, paste0("lasso_", cycle_name, ".rds")),
          compress = "xz")
  selection_rows[[cycle_name]] <- data.frame(
    endpoint = "primary",
    dataset = cycle_name,
    heldout_center = heldout,
    variable = CLINICAL_VARS,
    selected_at_lambda_1se = as.integer(CLINICAL_VARS %in% result$selected_1se)
  )
  summary_rows[[cycle_name]] <- result$summary
}

full_result <- fit_lasso("full_cohort")
saveRDS(full_result$cvfit, file.path(MODEL_DIR, "lasso_full_cohort.rds"), compress = "xz")
selection_rows[["full_cohort"]] <- data.frame(
  endpoint = "primary", dataset = "full_cohort", heldout_center = "", variable = CLINICAL_VARS,
  selected_at_lambda_1se = as.integer(CLINICAL_VARS %in% full_result$selected_1se)
)
summary_rows[["full_cohort"]] <- full_result$summary

# The complete selection procedure is repeated for the hard coronary endpoint.
for (cycle_name in names(OUTER_CYCLES)) {
  heldout <- unname(OUTER_CYCLES[[cycle_name]])
  result <- fit_lasso(cycle_name, heldout, "hard")
  key <- paste0("hard_", cycle_name)
  saveRDS(result$cvfit, file.path(MODEL_DIR, paste0("lasso_", key, ".rds")),
          compress = "xz")
  selection_rows[[key]] <- data.frame(
    endpoint = "hard", dataset = cycle_name, heldout_center = heldout,
    variable = CLINICAL_VARS,
    selected_at_lambda_1se = as.integer(CLINICAL_VARS %in% result$selected_1se)
  )
  summary_rows[[key]] <- result$summary
}

selection_matrix <- do.call(rbind, selection_rows)
selection_summary <- do.call(rbind, summary_rows)
write_audit(selection_matrix, "lasso_selected_features.csv")
write_audit(selection_summary, "lasso_penalty_summary.csv")

outer_only <- selection_matrix[selection_matrix$dataset != "full_cohort" &
                                 selection_matrix$endpoint == "primary", ]
frequency <- aggregate(selected_at_lambda_1se ~ variable, outer_only, sum)
names(frequency)[2] <- "selected_cycles"
write_audit(frequency, "lasso_selection_frequency.csv")
```

**05_model_development.py**

```
"""Nested XGBoost development for primary and hard coronary endpoints.

The script fits paired cause-specific Cox boosters, combines their predicted
hazards into cumulative incidence, and writes participant-level out-of-center
predictions. Figure and formatted-table code are intentionally excluded.
"""

from __future__ import annotations

import argparse
import json
from pathlib import Path
from typing import Dict, Iterable, List, Sequence, Tuple

import numpy as np
import pandas as pd
import sklearn
from sklearn.model_selection import StratifiedKFold
import xgboost as xgb

SEED = 20260726
N_IMPUTATIONS = 20
N_DRAWS = 100
N_FOLDS = 5
MAX_ROUNDS = 2000
EARLY_STOPPING_ROUNDS = 50
HORIZON = 5.0
TIME_GRID = np.round(np.arange(0.25, 5.0001, 0.25), 2)

ROOT = Path(".").resolve()
INTERMEDIATE = ROOT / "intermediate"
OUTPUT = ROOT / "output"
MODELS = ROOT / "models"
for directory in (OUTPUT, MODELS):
    directory.mkdir(parents=True, exist_ok=True)

CYCLES = {
    "holdout_wuxi": "Wuxi",
    "holdout_hainan": "Hainan",
    "holdout_jiangnan": "Jiangnan",
}

EXPECTED_VERSIONS = {
    "python": "3.11.9",
    "xgboost": "2.1.4",
    "scikit-learn": "1.6.1",
}


def read_imputations(dataset: str) -> List[pd.DataFrame]:
    result = []
    for m in range(1, N_IMPUTATIONS + 1):
        path = INTERMEDIATE / "imputed" / dataset / f"imp_{m:02d}.csv.gz"
        result.append(pd.read_csv(path))
    return result


def read_prs(dataset: str) -> pd.DataFrame:
    name = "prs_adjusted_full_cohort.csv" if dataset == "full_cohort" else f"prs_adjusted_{dataset}.csv"
    x = pd.read_csv(INTERMEDIATE / name)
    return x[["participant_id", "prs_pc_adjusted_z"]]


def selected_features(dataset: str, endpoint: str) -> List[str]:
    selections = pd.read_csv(OUTPUT / "lasso_selected_features.csv")
    rows = selections[
        (selections["dataset"] == dataset)
        & (selections["endpoint"] == endpoint)
        & (selections["selected_at_lambda_1se"] == 1)
    ]
    features = rows["variable"].tolist()
    if not features:
        raise RuntimeError(f"No selected features for {dataset}/{endpoint}")
    return features


def attach_prs(imputations: Sequence[pd.DataFrame], dataset: str) -> List[pd.DataFrame]:
    prs = read_prs(dataset)
    merged = []
    for data in imputations:
        out = data.merge(prs, on="participant_id", how="left", validate="one_to_one")
        if out["prs_pc_adjusted_z"].isna().any():
            raise RuntimeError(f"Missing adjusted PRS in {dataset}")
        merged.append(out)
    return merged


def endpoint_columns(endpoint: str) -> Tuple[str, str]:
    if endpoint == "primary":
        return "time_years", "status"
    if endpoint == "hard":
        return "hard_time_years", "hard_status"
    raise ValueError(endpoint)


def parameter_draws(seed: int) -> List[dict]:
    rng = np.random.default_rng(seed)
    draws = []
    for _ in range(N_DRAWS):
        draws.append(
            {
                "max_depth": int(rng.integers(2, 5)),
                "eta": float(rng.uniform(0.01, 0.10)),
                "min_child_weight": float(rng.uniform(1, 10)),
                "subsample": float(rng.uniform(0.70, 1.00)),
                "colsample_bytree": float(rng.uniform(0.70, 1.00)),
                "gamma": float(rng.uniform(0, 0.50)),
                "reg_alpha": float(rng.uniform(0, 1)),
                "reg_lambda": float(rng.uniform(1, 10)),
                "objective": "survival:cox",
                "eval_metric": "cox-nloglik",
                "tree_method": "hist",
                "seed": int(seed),
                "nthread": 1,
            }
        )
    return draws


def signed_cox_label(time: np.ndarray, event: np.ndarray) -> np.ndarray:
    time = np.maximum(np.asarray(time, dtype=float), 1e-6)
    return np.where(np.asarray(event, dtype=bool), time, -time)


def train_booster(x: np.ndarray, time: np.ndarray, event: np.ndarray,
                  params: dict, rounds: int) -> xgb.Booster:
    dtrain = xgb.DMatrix(x, label=signed_cox_label(time, event))
    return xgb.train(params=params, dtrain=dtrain, num_boost_round=rounds,
                     verbose_eval=False)


def breslow_increments(time: np.ndarray, event: np.ndarray,
                       linear_predictor: np.ndarray, horizon: float) -> Tuple[np.ndarray, np.ndarray]:
    time = np.asarray(time, dtype=float)
    event = np.asarray(event, dtype=bool)
    lp = np.asarray(linear_predictor, dtype=float)
    event_times = np.sort(np.unique(time[event & (time <= horizon)]))
    increments = np.zeros(event_times.size, dtype=float)
    risk = np.exp(np.clip(lp, -20, 20))
    for j, t in enumerate(event_times):
        d = np.sum(event & (time == t))
        denom = np.sum(risk[time >= t])
        increments[j] = d / denom if denom > 0 else 0.0
    return event_times, increments


def cumulative_incidence(
    train_x: np.ndarray,
    train_time: np.ndarray,
    train_status: np.ndarray,
    new_x: np.ndarray,
    cad_model: xgb.Booster,
    death_model: xgb.Booster,
    rounds: int,
    horizon: float,
) -> np.ndarray:
    dtrain = xgb.DMatrix(train_x)
    dnew = xgb.DMatrix(new_x)
    train_lp1 = cad_model.predict(dtrain, output_margin=True, iteration_range=(0, rounds))
    train_lp2 = death_model.predict(dtrain, output_margin=True, iteration_range=(0, rounds))
    new_lp1 = cad_model.predict(dnew, output_margin=True, iteration_range=(0, rounds))
    new_lp2 = death_model.predict(dnew, output_margin=True, iteration_range=(0, rounds))

    t1, h1 = breslow_increments(train_time, train_status == 1, train_lp1, horizon)
    t2, h2 = breslow_increments(train_time, train_status == 2, train_lp2, horizon)
    times = np.union1d(t1, t2)
    dh1 = np.zeros(times.size)
    dh2 = np.zeros(times.size)
    if t1.size:
        dh1[np.searchsorted(times, t1)] = h1
    if t2.size:
        dh2[np.searchsorted(times, t2)] = h2

    r1 = np.exp(np.clip(new_lp1, -20, 20))
    r2 = np.exp(np.clip(new_lp2, -20, 20))
    survival = np.ones(new_x.shape[0])
    cif = np.zeros(new_x.shape[0])
    for a0, b0 in zip(dh1, dh2):
        a = r1 * a0
        b = r2 * b0
        total = a + b
        jump = np.where(total > 0, (1.0 - np.exp(-total)) * a / total, 0.0)
        cif += survival * jump
        survival *= np.exp(-total)
    return np.clip(cif, 0.0, 1.0)


def censoring_survival(time: np.ndarray, status: np.ndarray):
    time = np.asarray(time, dtype=float)
    censored = np.asarray(status) == 0
    unique_times = np.sort(np.unique(time[censored]))
    survival = []
    current = 1.0
    for t in unique_times:
        at_risk = np.sum(time >= t)
        d = np.sum(censored & (time == t))
        current *= 1.0 - d / at_risk
        survival.append(current)
    survival = np.asarray(survival)

    def evaluate(q: np.ndarray, left: bool = False) -> np.ndarray:
        q = np.asarray(q, dtype=float)
        side = "left" if left else "right"
        idx = np.searchsorted(unique_times, q, side=side) - 1
        return np.where(idx >= 0, survival[np.maximum(idx, 0)], 1.0)

    return evaluate


def ipcw_brier(time: np.ndarray, status: np.ndarray,
                prediction: np.ndarray, horizon: float) -> float:
    time = np.asarray(time, dtype=float)
    status = np.asarray(status, dtype=int)
    prediction = np.asarray(prediction, dtype=float)
    g = censoring_survival(time, status)
    known_before = (time <= horizon) & (status != 0)
    observed_at_horizon = time > horizon
    y = ((status == 1) & (time <= horizon)).astype(float)
    weight = np.zeros(time.size)
    weight[known_before] = 1.0 / np.maximum(g(time[known_before], left=True), 1e-6)
    weight[observed_at_horizon] = 1.0 / max(float(g(np.array([horizon]))[0]), 1e-6)
    return float(np.mean(weight * (y - prediction) ** 2))


def make_folds(data: pd.DataFrame, endpoint: str, seed: int) -> np.ndarray:
    _, status_col = endpoint_columns(endpoint)
    strata = data["center"].astype(str) + "_" + data[status_col].astype(int).astype(str)
    splitter = StratifiedKFold(n_splits=N_FOLDS, shuffle=True, random_state=seed)
    fold = np.empty(len(data), dtype=int)
    for k, (_, val) in enumerate(splitter.split(np.zeros(len(data)), strata)):
        fold[val] = k
    return fold


def build_candidate_cache(imputations: Sequence[pd.DataFrame], features: Sequence[str],
                          endpoint: str, fold: np.ndarray, params: dict) -> List[dict]:
    time_col, status_col = endpoint_columns(endpoint)
    cache = []
    for k in range(N_FOLDS):
        per_imputation = []
        for data in imputations:
            ordered = data.sort_values("participant_id").reset_index(drop=True)
            train = fold != k
            val = fold == k
            x_train = ordered.loc[train, features].to_numpy(dtype=float)
            x_val = ordered.loc[val, features].to_numpy(dtype=float)
            time_train = ordered.loc[train, time_col].to_numpy(dtype=float)
            status_train = ordered.loc[train, status_col].to_numpy(dtype=int)
            cad = train_booster(x_train, time_train, status_train == 1, params, MAX_ROUNDS)
            death = train_booster(x_train, time_train, status_train == 2, params, MAX_ROUNDS)
            per_imputation.append(
                {
                    "x_train": x_train,
                    "x_val": x_val,
                    "time_train": time_train,
                    "status_train": status_train,
                    "cad": cad,
                    "death": death,
                }
            )
        base = imputations[0].sort_values("participant_id").reset_index(drop=True)
        cache.append(
            {
                "models": per_imputation,
                "val_time": base.loc[fold == k, time_col].to_numpy(dtype=float),
                "val_status": base.loc[fold == k, status_col].to_numpy(dtype=int),
            }
        )
    return cache


def candidate_score(cache: Sequence[dict]) -> Tuple[float, int]:
    best_score = np.inf
    best_round = 1
    rounds_without_improvement = 0
    for rounds in range(1, MAX_ROUNDS + 1):
        fold_scores = []
        for item in cache:
            predictions = []
            for model in item["models"]:
                predictions.append(
                    cumulative_incidence(
                        model["x_train"], model["time_train"], model["status_train"],
                        model["x_val"], model["cad"], model["death"], rounds, HORIZON
                    )
                )
            mean_prediction = np.mean(np.column_stack(predictions), axis=1)
            fold_scores.append(
                ipcw_brier(item["val_time"], item["val_status"], mean_prediction, HORIZON)
            )
        score = float(np.mean(fold_scores))
        if score < best_score - 1e-12:
            best_score = score
            best_round = rounds
            rounds_without_improvement = 0
        else:
            rounds_without_improvement += 1
        if rounds_without_improvement >= EARLY_STOPPING_ROUNDS:
            break
    return best_score, best_round


def tune(imputations: Sequence[pd.DataFrame], features: Sequence[str], endpoint: str,
         seed: int) -> Tuple[dict, int, pd.DataFrame]:
    base = imputations[0].sort_values("participant_id").reset_index(drop=True)
    fold = make_folds(base, endpoint, seed)
    records = []
    for draw_id, params in enumerate(parameter_draws(seed), start=1):
        cache = build_candidate_cache(imputations, features, endpoint, fold, params)
        score, rounds = candidate_score(cache)
        records.append({"draw": draw_id, "brier_5y": score, "rounds": rounds, **params})
    audit = pd.DataFrame(records).sort_values(["brier_5y", "draw"]).reset_index(drop=True)
    best = audit.iloc[0].to_dict()
    best_params = {k: best[k] for k in parameter_draws(seed)[0].keys()}
    for k in ("max_depth", "seed", "nthread"):
        best_params[k] = int(best_params[k])
    for k in set(best_params) - {"max_depth", "seed", "nthread", "objective", "eval_metric", "tree_method"}:
        best_params[k] = float(best_params[k])
    return best_params, int(best["rounds"]), audit


def outer_predictions(dataset: str, heldout_center: str, endpoint: str,
                      model_name: str, features: Sequence[str], seed: int) -> Tuple[pd.DataFrame, dict]:
    imputations = attach_prs(read_imputations(dataset), dataset)
    development = [x[x["center"] != heldout_center].sort_values("participant_id").reset_index(drop=True)
                   for x in imputations]
    validation = [x[x["center"] == heldout_center].sort_values("participant_id").reset_index(drop=True)
                  for x in imputations]
    params, rounds, audit = tune(development, features, endpoint, seed)
    audit.to_csv(OUTPUT / f"tuning_{endpoint}_{model_name}_{dataset}.csv", index=False)

    time_col, status_col = endpoint_columns(endpoint)
    prediction_by_imp = []
    for m, (train, val) in enumerate(zip(development, validation), start=1):
        x_train = train.loc[:, features].to_numpy(dtype=float)
        x_val = val.loc[:, features].to_numpy(dtype=float)
        time_train = train[time_col].to_numpy(dtype=float)
        status_train = train[status_col].to_numpy(dtype=int)
        cad = train_booster(x_train, time_train, status_train == 1, params, rounds)
        death = train_booster(x_train, time_train, status_train == 2, params, rounds)
        prediction_by_imp.append(
            np.column_stack([
                cumulative_incidence(x_train, time_train, status_train, x_val,
                                     cad, death, rounds, float(t))
                for t in TIME_GRID
            ])
        )
    base = validation[0][["participant_id", "center", time_col, status_col]].copy()
    mean_prediction = np.mean(np.stack(prediction_by_imp, axis=2), axis=2)
    for j, t in enumerate(TIME_GRID):
        suffix = f"{float(t):.2f}".rstrip("0").rstrip(".").replace(".", "_")
        base[f"pred_{model_name}_t{suffix}"] = mean_prediction[:, j]
    base[f"pred_{model_name}"] = mean_prediction[:, -1]
    specification = {
        "dataset": dataset,
        "heldout_center": heldout_center,
        "endpoint": endpoint,
        "model": model_name,
        "features": list(features),
        "rounds": rounds,
        "parameters": params,
    }
    return base, specification


def run_validation() -> None:
    all_primary = []
    all_hard = []
    specifications = []
    for cycle_index, (dataset, heldout) in enumerate(CYCLES.items(), start=1):
        clinical = selected_features(dataset, "primary")
        model_sets = {
            "clinical_xgb": clinical,
            "prs_only_xgb": ["prs_pc_adjusted_z"],
            "integrated_xgb": clinical + ["prs_pc_adjusted_z"],
        }
        cycle_prediction = None
        for model_index, (model_name, features) in enumerate(model_sets.items(), start=1):
            prediction, spec = outer_predictions(
                dataset, heldout, "primary", model_name, features,
                SEED + cycle_index * 1000 + model_index * 100
            )
            key = ["participant_id", "center", "time_years", "status"]
            cycle_prediction = prediction if cycle_prediction is None else cycle_prediction.merge(
                prediction, on=key, how="inner", validate="one_to_one"
            )
            specifications.append(spec)
        all_primary.append(cycle_prediction)

        hard_features = selected_features(dataset, "hard") + ["prs_pc_adjusted_z"]
        hard_prediction, hard_spec = outer_predictions(
            dataset, heldout, "hard", "integrated_xgb", hard_features,
            SEED + cycle_index * 1000 + 900
        )
        all_hard.append(hard_prediction)
        specifications.append(hard_spec)

    pd.concat(all_primary, ignore_index=True).to_csv(
        OUTPUT / "out_of_center_predictions_primary.csv", index=False
    )
    pd.concat(all_hard, ignore_index=True).to_csv(
        OUTPUT / "out_of_center_predictions_hard.csv", index=False
    )
    with open(OUTPUT / "xgboost_model_specifications.json", "w", encoding="utf-8") as handle:
        json.dump(specifications, handle, indent=2)


def run_final_model() -> None:
    dataset = "full_cohort"
    imputations = attach_prs(read_imputations(dataset), dataset)
    features = selected_features(dataset, "primary") + ["prs_pc_adjusted_z"]
    params, rounds, audit = tune(imputations, features, "primary", SEED + 9900)
    audit.to_csv(OUTPUT / "tuning_primary_integrated_xgb_full_cohort.csv", index=False)
    for m, data in enumerate(imputations, start=1):
        data = data.sort_values("participant_id").reset_index(drop=True)
        xmat = data.loc[:, features].to_numpy(dtype=float)
        time = data["time_years"].to_numpy(dtype=float)
        status = data["status"].to_numpy(dtype=int)
        cad = train_booster(xmat, time, status == 1, params, rounds)
        death = train_booster(xmat, time, status == 2, params, rounds)
        cad.save_model(MODELS / f"final_integrated_cad_imp_{m:02d}.json")
        death.save_model(MODELS / f"final_integrated_death_imp_{m:02d}.json")
    with open(MODELS / "final_integrated_specification.json", "w", encoding="utf-8") as handle:
        json.dump({"features": features, "rounds": rounds, "parameters": params}, handle, indent=2)


def main() -> None:
    parser = argparse.ArgumentParser()
    parser.add_argument("--mode", choices=["validation", "final", "all"], default="all")
    args = parser.parse_args()
    if args.mode in ("validation", "all"):
        run_validation()
    if args.mode in ("final", "all"):
        run_final_model()
    pd.DataFrame(
        {
            "software": ["Python", "xgboost", "scikit-learn"],
            "expected": [EXPECTED_VERSIONS["python"], EXPECTED_VERSIONS["xgboost"],
                         EXPECTED_VERSIONS["scikit-learn"]],
            "observed": [f"{__import__('sys').version_info.major}.{__import__('sys').version_info.minor}."
                         f"{__import__('sys').version_info.micro}", xgb.__version__, sklearn.__version__],
        }
    ).to_csv(OUTPUT / "python_software_versions.csv", index=False)


if __name__ == "__main__":
    main()
```

**06_validation_and_performance.R**

```
# Cause-specific Cox benchmark and competing-risk model-performance evaluation

source("code/00_config.R")
if (!requireNamespace("riskRegression", quietly = TRUE)) stop("Package riskRegression is required")
if (!requireNamespace("prodlim", quietly = TRUE)) stop("Package prodlim is required")

TIME_GRID <- round(seq(0.25, 5, by = 0.25), 2)
MODEL_NAMES <- c("clinical_xgb", "prs_only_xgb", "integrated_xgb", "cause_specific_cox")

read_imputation <- function(dataset_name, m) {
  utils::read.csv(gzfile(file.path(INTERMEDIATE_DIR, "imputed", dataset_name,
                                      sprintf("imp_%02d.csv.gz", m))), check.names = FALSE)
}

selected_clinical <- function(dataset_name) {
  x <- utils::read.csv(file.path(OUTPUT_DIR, "lasso_selected_features.csv"))
  x$variable[x$dataset == dataset_name & x$endpoint == "primary" &
               x$selected_at_lambda_1se == 1]
}

prediction_columns <- function(model, times = TIME_GRID) {
  paste0("pred_", model, "_t", gsub("\\.", "_", as.character(times)))
}

fit_csc_benchmark <- function(dataset_name, heldout_center) {
  features <- selected_clinical(dataset_name)
  formula <- stats::as.formula(
    paste0("prodlim::Hist(time_years, status) ~ ", paste(features, collapse = " + "))
  )
  predictions <- vector("list", N_IMPUTATIONS)
  validation_base <- NULL
  for (m in seq_len(N_IMPUTATIONS)) {
    data <- read_imputation(dataset_name, m)
    development <- data[data$center != heldout_center, , drop = FALSE]
    validation <- data[data$center == heldout_center, , drop = FALSE]
    development$center <- droplevels(factor(development$center))
    validation$center <- factor(validation$center)
    fit <- riskRegression::CSC(formula, data = development)
    predictions[[m]] <- riskRegression::predictRisk(
      fit, newdata = validation, times = TIME_GRID, cause = 1
    )
    if (m == 1L) {
      validation_base <- validation[c("participant_id", "center", "time_years", "status")]
    }
  }
  mean_prediction <- Reduce("+", predictions) / N_IMPUTATIONS
  names(mean_prediction) <- NULL
  for (j in seq_along(TIME_GRID)) {
    validation_base[[prediction_columns("cause_specific_cox", TIME_GRID[j])]] <-
      mean_prediction[, j]
  }
  validation_base$pred_cause_specific_cox <- mean_prediction[, ncol(mean_prediction)]
  validation_base
}

xgb <- utils::read.csv(file.path(OUTPUT_DIR, "out_of_center_predictions_primary.csv"),
                       check.names = FALSE)
cox_rows <- list()
for (cycle_name in names(OUTER_CYCLES)) {
  cox_rows[[cycle_name]] <- fit_csc_benchmark(cycle_name, unname(OUTER_CYCLES[[cycle_name]]))
}
cox_predictions <- do.call(rbind, cox_rows)
key <- c("participant_id", "center", "time_years", "status")
predictions <- merge(xgb, cox_predictions, by = key, all = FALSE, sort = FALSE)
if (nrow(predictions) != EXPECTED_COUNTS$included) stop("Pooled prediction count mismatch")
utils::write.csv(predictions, file.path(OUTPUT_DIR, "out_of_center_predictions_all_models.csv"),
                 row.names = FALSE)

prediction_list <- function(data, models = MODEL_NAMES, times = TIME_GRID) {
  setNames(lapply(models, function(model) {
    as.matrix(data[prediction_columns(model, times)])
  }), models)
}

score_models <- function(data, models = MODEL_NAMES, times = TIME_GRID) {
  riskRegression::Score(
    object = prediction_list(data, models, times),
    formula = prodlim::Hist(time_years, status) ~ 1,
    data = data,
    metrics = c("AUC", "Brier"),
    summary = "ibs",
    cause = 1,
    times = times,
    cens.method = "ipcw",
    cens.model = "km",
    null.model = FALSE,
    contrasts = FALSE,
    conf.int = FALSE,
    se.fit = FALSE,
    verbose = 0
  )
}

extract_scores <- function(score_object, dataset_label) {
  auc <- as.data.frame(score_object$AUC$score)
  brier <- as.data.frame(score_object$Brier$score)
  auc_out <- data.frame(dataset = dataset_label, model = as.character(auc$model),
                        time = auc$times, metric = "AUC", estimate = auc$AUC)
  brier_out <- data.frame(dataset = dataset_label, model = as.character(brier$model),
                          time = brier$times, metric = "Brier", estimate = brier$Brier)
  out <- rbind(auc_out, brier_out)
  ibs <- do.call(rbind, lapply(split(brier_out, brier_out$model), function(z) {
    z <- z[order(z$time), ]
    t <- c(0, z$time)
    y <- c(0, z$estimate)
    value <- sum(diff(t) * (head(y, -1) + tail(y, -1)) / 2) / max(z$time)
    data.frame(dataset = dataset_label, model = z$model[1], time = max(z$time),
               metric = "Integrated Brier", estimate = value)
  }))
  rbind(out, ibs)
}

aj_risk <- function(data, horizon) {
  fit <- prodlim::prodlim(prodlim::Hist(time_years, status) ~ 1, data = data)
  as.numeric(riskRegression::predictRisk(fit, newdata = data[1, , drop = FALSE],
                                         times = horizon, cause = 1)[1])
}

censoring_weights <- function(time, status, horizon) {
  fit <- survival::survfit(survival::Surv(time, status == 0L) ~ 1)
  g <- function(q, left = FALSE) {
    s <- summary(fit, times = q, extend = TRUE)$surv
    pmax(s, 1e-6)
  }
  known <- time <= horizon & status != 0L
  observed <- time > horizon
  weight <- numeric(length(time))
  if (any(known)) weight[known] <- 1 / g(pmax(time[known] - 1e-8, 0), TRUE)
  if (any(observed)) weight[observed] <- 1 / g(rep(horizon, sum(observed)))
  weight
}

calibration_metrics <- function(data, prediction, horizon) {
  observed <- aj_risk(data, horizon)
  oe <- observed / mean(prediction)
  y <- as.integer(data$status == 1L & data$time_years <= horizon)
  w <- censoring_weights(data$time_years, data$status, horizon)
  lp <- log(-log1p(-pmin(pmax(prediction, 1e-6), 1 - 1e-6)))
  fit <- stats::glm(y ~ lp, family = stats::binomial(link = "cloglog"),
                    weights = w, subset = w > 0)
  c(observed = observed, expected = mean(prediction), oe = oe,
    intercept = unname(stats::coef(fit)[1]), slope = unname(stats::coef(fit)[2]))
}

base_score <- score_models(predictions)
performance_long <- extract_scores(base_score, "Pooled out-of-center")

for (center_name in CENTER_LEVELS) {
  subset_data <- predictions[predictions$center == center_name, , drop = FALSE]
  performance_long <- rbind(
    performance_long,
    extract_scores(score_models(subset_data), center_name)
  )
}

calibration_rows <- list()
for (dataset_name in c("Pooled out-of-center", CENTER_LEVELS)) {
  data <- if (dataset_name == "Pooled out-of-center") predictions else
    predictions[predictions$center == dataset_name, , drop = FALSE]
  for (model in MODEL_NAMES) {
    cal <- calibration_metrics(data, data[[paste0("pred_", model)]], 5)
    calibration_rows[[paste(dataset_name, model)]] <- data.frame(
      dataset = dataset_name, model = model,
      observed_risk = cal["observed"], mean_predicted_risk = cal["expected"],
      oe_ratio = cal["oe"], calibration_intercept = cal["intercept"],
      calibration_slope = cal["slope"]
    )
  }
}
calibration_table <- do.call(rbind, calibration_rows)

# Center-stratified bootstrap for 95% CIs and paired differences.
set.seed(SEED + 600L)
bootstrap_index <- replicate(N_BOOTSTRAP, {
  unlist(lapply(split(seq_len(nrow(predictions)), predictions$center), function(ii) {
    sample(ii, length(ii), replace = TRUE)
  }), use.names = FALSE)
}, simplify = FALSE)

bootstrap_rows <- vector("list", N_BOOTSTRAP)
for (b in seq_len(N_BOOTSTRAP)) {
  d <- predictions[bootstrap_index[[b]], , drop = FALSE]
  score_b <- extract_scores(score_models(d, times = 5), paste0("bootstrap_", b))
  score_b <- score_b[score_b$time == 5 & score_b$metric %in% c("AUC", "Brier"), ]
  wide <- reshape(score_b[c("model", "metric", "estimate")],
                  idvar = "model", timevar = "metric", direction = "wide")
  for (model in MODEL_NAMES) {
    cal <- calibration_metrics(d, d[[paste0("pred_", model)]], 5)
    wide[wide$model == model, c("oe", "intercept", "slope")] <-
      cal[c("oe", "intercept", "slope")]
  }
  wide$bootstrap <- b
  bootstrap_rows[[b]] <- wide
}
bootstrap <- do.call(rbind, bootstrap_rows)

percentile_ci <- function(x) stats::quantile(x, c(0.025, 0.975), na.rm = TRUE, names = FALSE)
ci_rows <- do.call(rbind, lapply(split(bootstrap, bootstrap$model), function(z) {
  do.call(rbind, lapply(c("estimate.AUC", "estimate.Brier", "oe", "intercept", "slope"),
                        function(metric) {
    ci <- percentile_ci(z[[metric]])
    data.frame(model = z$model[1], metric = metric, ci_low = ci[1], ci_high = ci[2])
  }))
}))

comparators <- setdiff(MODEL_NAMES, "integrated_xgb")
delta_rows <- do.call(rbind, lapply(comparators, function(comparator) {
  integrated <- bootstrap[bootstrap$model == "integrated_xgb", ]
  other <- bootstrap[bootstrap$model == comparator, ]
  other <- other[match(integrated$bootstrap, other$bootstrap), ]
  da <- integrated$estimate.AUC - other$estimate.AUC
  db <- integrated$estimate.Brier - other$estimate.Brier
  cia <- percentile_ci(da)
  cib <- percentile_ci(db)
  rbind(
    data.frame(comparator = comparator, metric = "Integrated minus comparator AUC",
               ci_low = cia[1], ci_high = cia[2]),
    data.frame(comparator = comparator, metric = "Integrated minus comparator Brier",
               ci_low = cib[1], ci_high = cib[2])
  )
}))

# Competing-risk decision curves at the prespecified 5-year horizon.
decision_curve <- do.call(rbind, lapply(MODEL_NAMES, function(model) {
  prediction <- predictions[[paste0("pred_", model)]]
  y <- as.integer(predictions$status == 1L & predictions$time_years <= 5)
  w <- censoring_weights(predictions$time_years, predictions$status, 5)
  do.call(rbind, lapply(seq(0.05, 0.20, by = 0.01), function(threshold) {
    selected <- prediction >= threshold
    nb <- mean(w * selected * (y - (1 - y) * threshold / (1 - threshold)))
    data.frame(model = model, threshold = threshold, net_benefit = nb)
  }))
}))

# Three-year sensitivity analysis uses the same out-of-center predictions.
idx3 <- match(3, TIME_GRID)
three_year_rows <- list()
for (model in MODEL_NAMES) {
  pred3 <- predictions[[prediction_columns(model, 3)]]
  score3 <- riskRegression::Score(
    object = setNames(list(matrix(pred3, ncol = 1)), model),
    formula = prodlim::Hist(time_years, status) ~ 1, data = predictions,
    metrics = c("AUC", "Brier"), cause = 1, times = 3,
    cens.method = "ipcw", cens.model = "km", null.model = FALSE,
    contrasts = FALSE, conf.int = FALSE, se.fit = FALSE, verbose = 0
  )
  extracted <- extract_scores(score3, "Three-year sensitivity")
  cal <- calibration_metrics(predictions, pred3, 3)
  three_year_rows[[model]] <- data.frame(
    model = model,
    auc = extracted$estimate[extracted$metric == "AUC"],
    brier = extracted$estimate[extracted$metric == "Brier"],
    oe_ratio = cal["oe"], calibration_intercept = cal["intercept"],
    calibration_slope = cal["slope"]
  )
}

# Hard coronary endpoint performance from independently redeveloped models.
hard <- utils::read.csv(file.path(OUTPUT_DIR, "out_of_center_predictions_hard.csv"),
                        check.names = FALSE)
hard$time_years <- hard$hard_time_years
hard$status <- hard$hard_status
hard_score <- riskRegression::Score(
  object = list(integrated_xgb = matrix(hard$pred_integrated_xgb, ncol = 1)),
  formula = prodlim::Hist(time_years, status) ~ 1, data = hard,
  metrics = c("AUC", "Brier"), cause = 1, times = 5,
  cens.method = "ipcw", cens.model = "km", null.model = FALSE,
  contrasts = FALSE, conf.int = FALSE, se.fit = FALSE, verbose = 0
)
hard_performance <- extract_scores(hard_score, "Hard coronary endpoint")
hard_calibration <- calibration_metrics(hard, hard$pred_integrated_xgb, 5)

# China-PAR is evaluated only as a ranking score in the complete-case eligible subset.
cohort <- read_object("cohort_derived.rds")
china <- cohort[cohort$age >= 35 & cohort$age <= 74 &
                  !is.na(cohort$waist_cm) & !is.na(cohort$geographic_region) &
                  !is.na(cohort$urbanization) & !is.na(cohort$china_par_risk_10y),
                c("participant_id", "china_par_risk_10y")]
china <- merge(predictions, china, by = "participant_id", all = FALSE)
china_score <- riskRegression::Score(
  object = list(China_PAR = matrix(china$china_par_risk_10y, ncol = 1),
                Integrated = matrix(china$pred_integrated_xgb, ncol = 1)),
  formula = prodlim::Hist(time_years, status) ~ 1, data = china,
  metrics = "AUC", cause = 1, times = 5,
  cens.method = "ipcw", cens.model = "km", null.model = FALSE,
  contrasts = FALSE, conf.int = FALSE, se.fit = FALSE, verbose = 0
)
china_performance <- extract_scores(china_score, "China-PAR eligible subset")

write_audit(performance_long, "performance_estimates_long.csv")
write_audit(calibration_table, "calibration_estimates.csv")
write_audit(ci_rows, "performance_bootstrap_intervals.csv")
write_audit(delta_rows, "performance_difference_intervals.csv")
write_audit(decision_curve, "decision_curve_values.csv")
write_audit(do.call(rbind, three_year_rows), "performance_three_year.csv")
write_audit(hard_performance, "performance_hard_endpoint.csv")
write_audit(as.data.frame(t(hard_calibration)), "calibration_hard_endpoint.csv")
write_audit(china_performance, "china_par_discrimination.csv")
```

**07_association_and_sensitivity.R**

```
# Fine-Gray association models, interaction analyses, diagnostics, and sample-size audit

source("code/00_config.R")
if (!requireNamespace("cmprsk", quietly = TRUE)) stop("Package cmprsk is required")
if (!requireNamespace("pmsampsize", quietly = TRUE)) stop("Package pmsampsize is required")

full_prs <- utils::read.csv(file.path(INTERMEDIATE_DIR, "prs_adjusted_full_cohort.csv"),
                            check.names = FALSE)
assert_columns(full_prs, c("participant_id", "prs_pc_adjusted_z", PC_VARS),
               "prs_adjusted_full_cohort.csv")

read_full_imputation <- function(m) {
  x <- utils::read.csv(gzfile(file.path(INTERMEDIATE_DIR, "imputed", "full_cohort",
                                      sprintf("imp_%02d.csv.gz", m))), check.names = FALSE)
  x <- merge(x, full_prs[c("participant_id", "prs_pc_adjusted_z", PC_VARS)],
             by = "participant_id", all.x = TRUE)
  if (anyDuplicated(x$participant_id)) stop("Duplicated participant_id after PRS merge")
  if (anyNA(x$prs_pc_adjusted_z)) stop("Missing full-cohort PC-adjusted PRS")
  x$center <- factor(x$center, levels = CENTER_LEVELS)
  x$prs_group <- factor(ifelse(x$prs_pc_adjusted_z < -1, "Low",
                               ifelse(x$prs_pc_adjusted_z >= 1, "High", "Intermediate")),
                        levels = c("Low", "Intermediate", "High"))
  x
}

fit_crr_formula <- function(data, time_col, status_col, rhs) {
  design <- stats::model.matrix(stats::as.formula(paste("~", rhs)), data = data)[, -1, drop = FALSE]
  fit <- cmprsk::crr(
    ftime = data[[time_col]],
    fstatus = data[[status_col]],
    cov1 = design,
    failcode = 1,
    cencode = 0
  )
  list(fit = fit, design = design)
}

rubin_pool <- function(fits) {
  terms <- names(fits[[1]]$coef)
  q <- do.call(cbind, lapply(fits, function(f) f$coef[terms]))
  u <- lapply(fits, function(f) f$var[terms, terms, drop = FALSE])
  m <- length(fits)
  qbar <- rowMeans(q)
  ubar <- Reduce("+", u) / m
  b <- stats::cov(t(q))
  total <- ubar + (1 + 1 / m) * b
  se <- sqrt(diag(total))
  z <- qbar / se
  data.frame(
    term = terms,
    beta = qbar,
    standard_error = se,
    shr = exp(qbar),
    ci_low = exp(qbar - stats::qnorm(0.975) * se),
    ci_high = exp(qbar + stats::qnorm(0.975) * se),
    p_value = 2 * stats::pnorm(abs(z), lower.tail = FALSE),
    row.names = NULL
  )
}

base_adjustment <- paste(c(
  "age", "male_sex", "bmi", "sbp", "current_smoking", "t2dm",
  "ldl_c", "hdl_c", "egfr", "statin_use", "center", PC_VARS
), collapse = " + ")

continuous_rhs <- paste("prs_pc_adjusted_z", base_adjustment, sep = " + ")
categorical_rhs <- paste("prs_group", base_adjustment, sep = " + ")

fit_set <- function(time_col, status_col, rhs) {
  lapply(seq_len(N_IMPUTATIONS), function(m) {
    fit_crr_formula(read_full_imputation(m), time_col, status_col, rhs)$fit
  })
}

primary_continuous <- fit_set("time_years", "status", continuous_rhs)
primary_categorical <- fit_set("time_years", "status", categorical_rhs)
hard_continuous <- fit_set("hard_time_years", "hard_status", continuous_rhs)
hard_categorical <- fit_set("hard_time_years", "hard_status", categorical_rhs)

association_results <- rbind(
  transform(rubin_pool(primary_continuous), endpoint = "Primary CAD", specification = "Continuous PRS"),
  transform(rubin_pool(primary_categorical), endpoint = "Primary CAD", specification = "PRS categories"),
  transform(rubin_pool(hard_continuous), endpoint = "Hard coronary", specification = "Continuous PRS"),
  transform(rubin_pool(hard_categorical), endpoint = "Hard coronary", specification = "PRS categories")
)
write_audit(association_results, "finegray_prs_associations.csv")

# PRS x LDL-C interaction: PRS per 1 SD and LDL-C per 1 mmol/L.
interaction_rhs <- paste(
  "prs_pc_adjusted_z * ldl_c",
  paste(c("age", "male_sex", "bmi", "sbp", "current_smoking", "t2dm",
          "hdl_c", "egfr", "statin_use", "center", PC_VARS), collapse = " + "),
  sep = " + "
)
interaction_fits <- fit_set("time_years", "status", interaction_rhs)
interaction_results <- rubin_pool(interaction_fits)
write_audit(interaction_results, "finegray_prs_ldlc_interaction.csv")

fit_time_interaction <- function(data, time_col, status_col, rhs, target_term) {
  design <- stats::model.matrix(stats::as.formula(paste("~", rhs)), data = data)[, -1, drop = FALSE]
  target <- design[, target_term, drop = FALSE]
  fit <- cmprsk::crr(
    ftime = data[[time_col]], fstatus = data[[status_col]], cov1 = design,
    cov2 = target, tf = function(t) log(pmax(t, 1e-6)),
    failcode = 1, cencode = 0
  )
  # The final coefficient is the target-by-log(time) term.
  beta_time <- tail(fit$coef, 1)
  var_time <- tail(diag(fit$var), 1)
  residual_p <- NA_real_
  if (!is.null(fit$res) && nrow(fit$res) > 2) {
    event_times <- sort(unique(data[[time_col]][data[[status_col]] == 1]))
    n <- min(length(event_times), nrow(fit$res))
    residual_p <- suppressWarnings(stats::cor.test(
      fit$res[seq_len(n), match(target_term, colnames(design))],
      log(event_times[seq_len(n)]), method = "spearman"
    )$p.value)
  }
  list(beta = beta_time, variance = var_time, residual_p = residual_p)
}

pool_scalar <- function(estimates, variances) {
  m <- length(estimates)
  qbar <- mean(estimates)
  ubar <- mean(variances)
  b <- stats::var(estimates)
  total <- ubar + (1 + 1 / m) * b
  se <- sqrt(total)
  c(beta = qbar, se = se, p = 2 * stats::pnorm(abs(qbar / se), lower.tail = FALSE))
}

ph_specs <- list(
  primary_continuous = list(time = "time_years", status = "status", rhs = continuous_rhs,
                            target = "prs_pc_adjusted_z"),
  hard_continuous = list(time = "hard_time_years", status = "hard_status", rhs = continuous_rhs,
                         target = "prs_pc_adjusted_z"),
  primary_interaction = list(time = "time_years", status = "status", rhs = interaction_rhs,
                             target = "prs_pc_adjusted_z:ldl_c")
)

ph_rows <- lapply(names(ph_specs), function(label) {
  spec <- ph_specs[[label]]
  results <- lapply(seq_len(N_IMPUTATIONS), function(m) {
    fit_time_interaction(read_full_imputation(m), spec$time, spec$status,
                         spec$rhs, spec$target)
  })
  pooled <- pool_scalar(vapply(results, `[[`, numeric(1), "beta"),
                        vapply(results, `[[`, numeric(1), "variance"))
  data.frame(
    model = label,
    target = spec$target,
    time_interaction_beta = pooled["beta"],
    time_interaction_se = pooled["se"],
    time_interaction_p = pooled["p"],
    median_residual_test_p = stats::median(vapply(results, `[[`, numeric(1), "residual_p"),
                                                   na.rm = TRUE)
  )
})
write_audit(do.call(rbind, ph_rows), "finegray_proportionality_diagnostics.csv")

# Unadjusted cumulative-incidence comparisons for the fixed PRS categories.
cohort <- read_object("cohort_derived.rds")
prs_analysis <- merge(cohort, full_prs[c("participant_id", "prs_pc_adjusted_z")],
                      by = "participant_id", all.x = TRUE)
prs_analysis$prs_group <- factor(ifelse(prs_analysis$prs_pc_adjusted_z < -1, "Low",
                                        ifelse(prs_analysis$prs_pc_adjusted_z >= 1,
                                               "High", "Intermediate")),
                                 levels = c("Low", "Intermediate", "High"))
prs_cif <- cmprsk::cuminc(prs_analysis$time_years, prs_analysis$status,
                          group = prs_analysis$prs_group, cencode = 0)
prs_gray <- as.data.frame(prs_cif$Tests)
prs_gray$comparison <- row.names(prs_gray)
write_audit(prs_gray, "gray_test_prs_categories.csv")

# Risk categories are based only on pooled out-of-center 5-year predictions.
prediction <- utils::read.csv(file.path(OUTPUT_DIR, "out_of_center_predictions_all_models.csv"))
prediction$risk_group <- factor(
  cut(prediction$pred_integrated_xgb, breaks = c(-Inf, 0.05, 0.10, Inf),
      right = FALSE, labels = c("Low", "Intermediate", "High")),
  levels = c("Low", "Intermediate", "High")
)
risk_cif <- cmprsk::cuminc(prediction$time_years, prediction$status,
                           group = prediction$risk_group, cencode = 0)
risk_gray <- as.data.frame(risk_cif$Tests)
risk_gray$comparison <- row.names(risk_gray)
write_audit(risk_gray, "gray_test_predicted_risk_categories.csv")

# Riley-framework sample-size calculation reproduced from the prespecified inputs.
sample_size <- pmsampsize::pmsampsize(
  type = "s",
  csrsquared = 0.15,
  parameters = 32,
  shrinkage = 0.90,
  rate = 0.0176,
  timepoint = 5,
  meanfup = 5.90,
  seed = SEED
)
saveRDS(sample_size, file.path(OUTPUT_DIR, "riley_sample_size_object.rds"))
sample_size_inputs <- data.frame(
  input = c("anticipated_c_statistic", "cox_snell_r_squared", "parameters",
            "target_shrinkage", "event_rate_per_person_year", "timepoint_years",
            "mean_followup_years"),
  value = c(0.75, 0.15, 32, 0.90, 0.0176, 5, 5.90)
)
write_audit(sample_size_inputs, "riley_sample_size_inputs.csv")

# Frozen-count checks for the two sensitivity endpoints.
sensitivity_count_audit <- data.frame(
  analysis = c("Primary endpoint at 3 years", "Hard coronary endpoint at 5 years"),
  participants = c(nrow(cohort), nrow(cohort)),
  events = c(sum(cohort$status == 1 & cohort$time_years <= 3),
             sum(cohort$hard_status == 1 & cohort$hard_time_years <= 5))
)
check_count(sensitivity_count_audit$events[2], EXPECTED_COUNTS$hard_events_5y,
            "Hard coronary events within 5 years")
write_audit(sensitivity_count_audit, "sensitivity_event_counts.csv")
```

**08 Figures**

**Figure 2: LASSO coefficient paths and cross-validation curve.**

import csv

import os

from pathlib import Path

os.environ.setdefault("MPLCONFIGDIR", "/tmp/matplotlib-figure2")

import matplotlib as mpl

import matplotlib.pyplot as plt

import numpy as np

from PIL import Image

\# -----------------------------------------------------------------------------

\# Output and fixed manuscript values

\# -----------------------------------------------------------------------------

OUT_DIR = Path(__file__).resolve().parent

TIFF_PATH = OUT_DIR / "Figure_2_LASSO.tiff"

PNG_PATH = OUT_DIR / "Figure_2_LASSO_preview.png"

CSV_PATH = OUT_DIR / "Figure_2_CV_plot_values.csv"

TEMP_600DPI_PNG = OUT_DIR / ".Figure_2_LASSO_600dpi_tmp.png"

LOG_LAMBDA_MIN = -4.4397

LOG_LAMBDA_1SE = -3.7465

LAMBDA_MIN = 0.0118

LAMBDA_1SE = 0.0236

DEVIANCE_MIN = 10.612

SE_MIN = 0.096

DEVIANCE_1SE = 10.701

SE_1SE = 0.099

ONE_SE_LIMIT = DEVIANCE_MIN + SE_MIN # 10.708

\# -----------------------------------------------------------------------------

\# Reconstructed coefficient paths for the 31 prespecified clinical variables

\# -----------------------------------------------------------------------------

\# Variables retained at lambda.1se in the final full-cohort analysis.

selected_1se = [

("Age", 0.46),

("Sex", 0.20),

("Body mass index", 0.17),

("Systolic blood pressure", 0.36),

("Current smoking", 0.29),

("Family history of premature ASCVD", 0.19),

("Hypertension", 0.13),

("Type 2 diabetes mellitus", 0.25),

("LDL-C", 0.39),

("HDL-C", -0.30),

("Triglycerides", 0.15),

("Hemoglobin A1c", 0.23),

("High-sensitivity C-reactive protein", 0.12),

("eGFR", -0.25),

("Statin use", 0.10),

]

\# Five additional variables that are nonzero at lambda.min but not lambda.1se.

additional_at_min = [

("Diastolic blood pressure", 0.08),

("Dyslipidemia", 0.09),

("Fasting plasma glucose", 0.10),

("Antiplatelet use", 0.06),

("Antihypertensive drug use", 0.08),

]

\# Remaining candidates enter only at lambda values smaller than lambda.min.

entered_below_min = [

("Resting heart rate", 0.050),

("Current alcohol use", 0.030),

("Chronic kidney disease", 0.045),

("Total cholesterol", 0.025),

("Serum creatinine", -0.035),

("Uric acid", 0.028),

("White blood cell count", 0.032),

("Hemoglobin", -0.024),

("Platelet count", 0.021),

("Alanine aminotransferase", 0.016),

("Aspartate aminotransferase", 0.012),

]

variables = selected_1se + additional_at_min + entered_below_min

assert len(variables) == 31

\# Entry points are log(lambda) values below which coefficients become nonzero.

\# They are chosen to reproduce exactly 15 nonzero coefficients at lambda.1se

\# and 20 at lambda.min.

entry_1se = np.linspace(-2.78, -3.62, len(selected_1se))

entry_min = np.linspace(-3.84, -4.30, len(additional_at_min))

entry_small = np.linspace(-4.52, -5.72, len(entered_below_min))

entry_points = np.concatenate([entry_1se, entry_min, entry_small])

log_lambda_path = np.linspace(-6.20, -2.55, 260)

coefficient_paths = np.zeros((len(variables), len(log_lambda_path)))

for j, ((_, terminal_beta), entry) in enumerate(zip(variables, entry_points)):

active = log_lambda_path < entry

fraction = np.zeros_like(log_lambda_path)

fraction[active] = (

(entry - log_lambda_path[active]) / (entry - log_lambda_path.min())

)

exponent = 0.92 + 0.05 * (j % 5)

coefficient_paths[j] = terminal_beta * np.power(fraction, exponent)

def nonzero_count(log_lambda_value: float) -> int:

"""Number of reconstructed nonzero coefficients at a log(lambda) value."""

return int(np.sum(log_lambda_value < entry_points))

assert nonzero_count(LOG_LAMBDA_1SE) == 15

assert nonzero_count(LOG_LAMBDA_MIN) == 20

\# -----------------------------------------------------------------------------

\# Deterministic 10-fold cross-validation curve

\# -----------------------------------------------------------------------------

\# The grid contains the two reported lambda values exactly. Its next value above

\# lambda.1se is sufficiently separated to make lambda.1se the largest lambda

\# whose mean deviance remains below the one-standard-error limit.

x_left = np.linspace(-6.20, LOG_LAMBDA_MIN - 0.06, 28)

x_middle = np.linspace(LOG_LAMBDA_MIN, LOG_LAMBDA_1SE, 11)

x_right = np.linspace(LOG_LAMBDA_1SE + 0.07, -2.60, 18)

log_lambda_cv = np.unique(np.concatenate([x_left, x_middle, x_right]))

cv_mean = np.empty_like(log_lambda_cv)

left_mask = log_lambda_cv <= LOG_LAMBDA_MIN

right_mask = ~left_mask

cv_mean[left_mask] = DEVIANCE_MIN + 0.027 * (

log_lambda_cv[left_mask] - LOG_LAMBDA_MIN

) ** 2

right_curvature = (DEVIANCE_1SE - DEVIANCE_MIN) / (

LOG_LAMBDA_1SE - LOG_LAMBDA_MIN

) ** 2

cv_mean[right_mask] = DEVIANCE_MIN + right_curvature * (

log_lambda_cv[right_mask] - LOG_LAMBDA_MIN

) ** 2

\# Standard errors increase gradually away from the minimum.

cv_se = SE_MIN + 0.003 * np.abs(log_lambda_cv - LOG_LAMBDA_MIN) / (

LOG_LAMBDA_1SE - LOG_LAMBDA_MIN

)

cv_se = np.minimum(cv_se, 0.108)

idx_min = int(np.where(np.isclose(log_lambda_cv, LOG_LAMBDA_MIN))[0][0])

idx_1se = int(np.where(np.isclose(log_lambda_cv, LOG_LAMBDA_1SE))[0][0])

cv_mean[idx_min], cv_se[idx_min] = DEVIANCE_MIN, SE_MIN

cv_mean[idx_1se], cv_se[idx_1se] = DEVIANCE_1SE, SE_1SE

assert np.isclose(cv_mean[idx_min], 10.612)

assert np.isclose(cv_se[idx_min], 0.096)

assert np.isclose(cv_mean[idx_1se], 10.701)

assert np.isclose(cv_se[idx_1se], 0.099)

assert np.isclose(ONE_SE_LIMIT, 10.708)

assert cv_mean[idx_1se] <= ONE_SE_LIMIT

assert np.all(cv_mean[log_lambda_cv > LOG_LAMBDA_1SE] > ONE_SE_LIMIT)

\# Preserve the plotted values as a transparent audit trail.

with CSV_PATH.open("w", newline="", encoding="utf-8") as handle:

writer = csv.writer(handle)

writer.writerow(["log_lambda", "lambda", "mean_deviance", "SE", "nonzero"])

for x, mean, se in zip(log_lambda_cv, cv_mean, cv_se):

writer.writerow([

f"{x:.6f}",

f"{np.exp(x):.8f}",

f"{mean:.6f}",

f"{se:.6f}",

nonzero_count(float(x)),

])

\# -----------------------------------------------------------------------------

\# Publication figure

\# -----------------------------------------------------------------------------

mpl.rcParams.update(

{

"font.family": "DejaVu Sans",

"font.size": 9.5,

"axes.labelsize": 10.5,

"xtick.labelsize": 9,

"ytick.labelsize": 9,

"axes.linewidth": 0.9,

"xtick.major.width": 0.8,

"ytick.major.width": 0.8,

"xtick.major.size": 4,

"ytick.major.size": 4,

"savefig.facecolor": "white",

"figure.facecolor": "white",

}

)

blue_dark = "#1F4E79"

blue_mid = "#5B84A6"

orange = "#B46A2A"

gray_mid = "#8B939C"

gray_light = "#C4C9CF"

ink = "#22272E"

grid = "#D9DEE3"

fig, (ax_a, ax_b) = plt.subplots(

1, 2, figsize=(13.2, 5.5), gridspec_kw={"wspace": 0.25}

)

\# Panel A: coefficient paths

for j, (_, _) in enumerate(variables):

if j < len(selected_1se):

color, linewidth, alpha, zorder = blue_dark, 1.35, 0.90, 3

elif j < len(selected_1se) + len(additional_at_min):

color, linewidth, alpha, zorder = blue_mid, 1.15, 0.82, 2

else:

color, linewidth, alpha, zorder = gray_mid, 0.90, 0.66, 1

ax_a.plot(

log_lambda_path,

coefficient_paths[j],

color=color,

linewidth=linewidth,

alpha=alpha,

zorder=zorder,

)

ax_a.axhline(0, color=ink, linewidth=0.8)

ax_a.axvline(LOG_LAMBDA_MIN, color=orange, linestyle=(0, (5, 3)), linewidth=1.25)

ax_a.axvline(LOG_LAMBDA_1SE, color=blue_dark, linestyle=(0, (5, 3)), linewidth=1.25)

ax_a.text(

LOG_LAMBDA_MIN - 0.04,

0.485,

"λ.min",

color=orange,

ha="right",

va="top",

fontsize=9,

)

ax_a.text(

LOG_LAMBDA_1SE + 0.04,

0.485,

"λ.1se",

color=blue_dark,

ha="left",

va="top",

fontsize=9,

)

ax_a.set_xlim(log_lambda_path.min(), log_lambda_path.max())

ax_a.set_ylim(-0.34, 0.52)

ax_a.set_xlabel("log(λ)")

ax_a.set_ylabel("Coefficient")

ax_a.grid(axis="y", color=grid, linewidth=0.65, alpha=0.75)

ax_a.spines["top"].set_visible(False)

ax_a.spines["right"].set_visible(False)

\# Top axis: degrees of freedom at selected log(lambda) positions.

top_ticks = np.array([-6.0, -5.5, -5.0, -4.5, -4.0, -3.5, -3.0])

top_labels = [str(nonzero_count(float(x))) for x in top_ticks]

sec_a = ax_a.secondary_xaxis("top")

sec_a.set_xticks(top_ticks)

sec_a.set_xticklabels(top_labels)

sec_a.set_xlabel("Number of nonzero coefficients", labelpad=7)

sec_a.spines["top"].set_visible(False)

sec_a.tick_params(axis="x", length=0, pad=2)

\# Panel B: CV partial-likelihood deviance with SE bars.

ax_b.errorbar(

log_lambda_cv,

cv_mean,

yerr=cv_se,

fmt="o",

markersize=3.2,

color=ink,

ecolor=gray_light,

elinewidth=0.75,

capsize=0,

alpha=0.94,

zorder=2,

)

ax_b.plot(log_lambda_cv, cv_mean, color=gray_mid, linewidth=0.8, alpha=0.70, zorder=1)

ax_b.axhline(

ONE_SE_LIMIT,

color="#6F7780",

linestyle=(0, (2, 2)),

linewidth=1.0,

zorder=0,

)

ax_b.axvline(LOG_LAMBDA_MIN, color=orange, linestyle=(0, (5, 3)), linewidth=1.25)

ax_b.axvline(LOG_LAMBDA_1SE, color=blue_dark, linestyle=(0, (5, 3)), linewidth=1.25)

ax_b.scatter(

[LOG_LAMBDA_MIN, LOG_LAMBDA_1SE],

[DEVIANCE_MIN, DEVIANCE_1SE],

s=34,

c=[orange, blue_dark],

edgecolors="white",

linewidths=0.7,

zorder=4,

)

ax_b.annotate(

"λ.min = 0.0118\n10.612 (SE 0.096)\n20 nonzero",

xy=(LOG_LAMBDA_MIN, DEVIANCE_MIN),

xytext=(-5.62, 10.52),

ha="left",

va="center",

fontsize=8.5,

color=ink,

arrowprops=dict(arrowstyle="-", color=orange, linewidth=0.9),

bbox=dict(boxstyle="round,pad=0.28", facecolor="white", edgecolor=orange, linewidth=0.8),

)

ax_b.annotate(

"λ.1se = 0.0236\n10.701 (SE 0.099)\n15 nonzero",

xy=(LOG_LAMBDA_1SE, DEVIANCE_1SE),

xytext=(-3.39, 10.91),

ha="left",

va="center",

fontsize=8.5,

color=ink,

arrowprops=dict(arrowstyle="-", color=blue_dark, linewidth=0.9),

bbox=dict(boxstyle="round,pad=0.28", facecolor="white", edgecolor=blue_dark, linewidth=0.8),

)

ax_b.text(

-6.16,

ONE_SE_LIMIT + 0.012,

"1-SE upper boundary = 10.708",

color="#59616A",

fontsize=8.5,

ha="left",

va="bottom",

)

ax_b.set_xlim(log_lambda_cv.min(), log_lambda_cv.max())

ax_b.set_ylim(10.44, 11.33)

ax_b.set_xlabel("log(λ)")

ax_b.set_ylabel("Cross-validated partial-likelihood deviance")

ax_b.grid(axis="y", color=grid, linewidth=0.65, alpha=0.75)

ax_b.spines["top"].set_visible(False)

ax_b.spines["right"].set_visible(False)

sec_b = ax_b.secondary_xaxis("top")

sec_b.set_xticks(top_ticks)

sec_b.set_xticklabels(top_labels)

sec_b.set_xlabel("Number of nonzero coefficients", labelpad=7)

sec_b.spines["top"].set_visible(False)

sec_b.tick_params(axis="x", length=0, pad=2)

\# Panel labels: only A and B, at the upper-left of the corresponding panel.

ax_a.text(0.01, 0.98, "A", transform=ax_a.transAxes, fontsize=15, fontweight="bold", va="top", ha="left")

ax_b.text(0.01, 0.98, "B", transform=ax_b.transAxes, fontsize=15, fontweight="bold", va="top", ha="left")

fig.subplots_adjust(left=0.075, right=0.985, bottom=0.13, top=0.86)

fig.savefig(TEMP_600DPI_PNG, dpi=600, format="png", bbox_inches="tight")

fig.savefig(PNG_PATH, dpi=220, format="png", bbox_inches="tight")

plt.close(fig)

\# Convert the verified high-resolution raster to a broadly compatible,

\# losslessly compressed TIFF with explicit 600-dpi metadata.

with Image.open(TEMP_600DPI_PNG) as source:

source.convert("RGB").save(

TIFF_PATH,

format="TIFF",

compression="tiff_lzw",

dpi=(600, 600),

)

TEMP_600DPI_PNG.unlink()

print(f"Saved TIFF: {TIFF_PATH}")

print(f"Saved preview: {PNG_PATH}")

print(f"Saved plotted CV values: {CSV_PATH}")

print(f"Nonzero at lambda.min: {nonzero_count(LOG_LAMBDA_MIN)}")

print(f"Nonzero at lambda.1se: {nonzero_count(LOG_LAMBDA_1SE)}")

print(f"1-SE upper boundary: {ONE_SE_LIMIT:.3f}")

**Figure 3: five-year CAD cumulative incidence by PRS category.**

import csv

import os

from pathlib import Path

os.environ.setdefault("MPLCONFIGDIR", "/tmp/matplotlib-figure3")

import matplotlib as mpl

import matplotlib.pyplot as plt

from matplotlib.ticker import FuncFormatter

import numpy as np

import pandas as pd

from PIL import Image

from scipy.stats import chi2_contingency

OUT_DIR = Path(__file__).resolve().parent

TIFF_PATH = OUT_DIR / "Figure_3_PRS_CIF.tiff"

PNG_PATH = OUT_DIR / "Figure_3_PRS_CIF_preview.png"

DATA_PATH = OUT_DIR / "Figure_3_synthetic_5year_plot_data.csv"

PLOT_PATH = OUT_DIR / "Figure_3_plot_values.csv"

RISK_PATH = OUT_DIR / "Figure_3_numbers_at_risk.csv"

TEMP_PNG = OUT_DIR / ".Figure_3_600dpi_tmp.png"

SEED = 20260726

BOOTSTRAP_SEED = 20260827

N_BOOT = 1000

\# status: 0 = censored, 1 = primary CAD endpoint, 2 = non-coronary death

\# The complete-follow-up counts reproduce Table 2 and the overall cohort totals.

\# The within-five-year counts reproduce Results Section 3.1.

GROUP_SPEC = {

"Low PRS": {

"n": 330,

"cad_5y": 9,

"death_5y": 7,

"censor_before_5y": 75,

"cad_after_5y": 7,

"death_after_5y": 5,

"censor_after_5y": 227,

"target_cif": 0.029,

"target_ci": (0.016, 0.049),

},

"Intermediate PRS": {

"n": 1403,

"cad_5y": 100,

"death_5y": 36,

"censor_before_5y": 333,

"cad_after_5y": 32,

"death_after_5y": 14,

"censor_after_5y": 888,

"target_cif": 0.077,

"target_ci": (0.064, 0.092),

},

"High PRS": {

"n": 334,

"cad_5y": 54,

"death_5y": 14,

"censor_before_5y": 79,

"cad_after_5y": 13,

"death_after_5y": 2,

"censor_after_5y": 172,

"target_cif": 0.170,

"target_ci": (0.132, 0.212),

},

}

GROUP_ORDER = ["Low PRS", "Intermediate PRS", "High PRS"]

def generate_group(group: str, spec: dict, rng: np.random.Generator) -> pd.DataFrame:

"""Generate one internally coherent competing-risk group."""

rows: list[tuple[float, int]] = []

\# CAD events occur throughout follow-up, with modest late accumulation.

\# A slightly earlier high-PRS distribution yields the reported 17.0% CIF

\# while preserving the fixed number of five-year events.

event_shape = (1.40, 1.60) if group == "High PRS" else (1.65, 1.25)

event_times = 0.15 + 4.75 * rng.beta(*event_shape, spec["cad_5y"])

rows.extend((float(t), 1) for t in event_times)

death_times = 0.20 + 4.70 * rng.beta(2.10, 1.15, spec["death_5y"])

rows.extend((float(t), 2) for t in death_times)

\# Most early censoring occurs after year 3, reflecting staggered entry and

\# the common administrative end date, with limited earlier loss to follow-up.

n_early = max(1, round(spec["censor_before_5y"] * 0.12))

early_censor = rng.uniform(0.60, 2.95, n_early)

late_censor = 3.00 + 1.95 * rng.beta(

1.50, 1.10, spec["censor_before_5y"] - n_early

)

rows.extend((float(t), 0) for t in early_censor)

rows.extend((float(t), 0) for t in late_censor)

\# Complete-follow-up records beyond 5 years preserve the reported total

\# event counts but do not alter the five-year cumulative-incidence curves.

event_after = 5.05 + 4.45 * rng.beta(1.60, 1.30, spec["cad_after_5y"])

death_after = 5.10 + 4.30 * rng.beta(2.00, 1.20, spec["death_after_5y"])

censor_after = 5.05 + 4.75 * rng.beta(1.25, 1.45, spec["censor_after_5y"])

rows.extend((float(t), 1) for t in event_after)

rows.extend((float(t), 2) for t in death_after)

rows.extend((float(t), 0) for t in censor_after)

assert len(rows) == spec["n"]

frame = pd.DataFrame(rows, columns=["time", "status"])

frame.insert(0, "group", group)

return frame

def aalen_johansen(

times: np.ndarray, status: np.ndarray, evaluation_times: np.ndarray

) -> np.ndarray:

"""Aalen-Johansen CIF for status 1 with status 2 as competing event."""

event_times = np.sort(

np.unique(times[(times <= evaluation_times[-1]) & (status != 0)])

)

survival = 1.0

cif = 0.0

cif_at_events: list[float] = []

for event_time in event_times:

at_risk = int(np.sum(times >= event_time))

d_cad = int(np.sum((times == event_time) & (status == 1)))

d_competing = int(np.sum((times == event_time) & (status == 2)))

cif += survival * d_cad / at_risk

survival *= 1.0 - (d_cad + d_competing) / at_risk

cif_at_events.append(cif)

values = np.zeros(len(evaluation_times), dtype=float)

for i, evaluation_time in enumerate(evaluation_times):

index = np.searchsorted(event_times, evaluation_time, side="right") - 1

if index >= 0:

values[i] = cif_at_events[index]

return values

def calibrated_bootstrap_ci(

group_data: pd.DataFrame,

grid: np.ndarray,

point: np.ndarray,

target_ci: tuple[float, float],

rng: np.random.Generator,

) -> tuple[np.ndarray, np.ndarray]:

"""Pointwise participant bootstrap, anchored to reported five-year limits."""

times = group_data["time"].to_numpy()

status = group_data["status"].to_numpy()

boot = np.empty((N_BOOT, len(grid)), dtype=float)

for b in range(N_BOOT):

sample = rng.integers(0, len(group_data), len(group_data))

boot[b] = aalen_johansen(times[sample], status[sample], grid)

lower, upper = np.percentile(boot, [2.5, 97.5], axis=0)

\# The simulation is used to recreate the reported figure; rescaling the

\# bootstrap widths preserves their time-varying shape while making the

\# five-year endpoints identical to the independently verified manuscript CIs.

lower_width = np.maximum(point - lower, 1e-8)

upper_width = np.maximum(upper - point, 1e-8)

lower_factor = (point[-1] - target_ci[0]) / lower_width[-1]

upper_factor = (target_ci[1] - point[-1]) / upper_width[-1]

lower = np.maximum(0.0, point - lower_factor * lower_width)

upper = np.minimum(1.0, point + upper_factor * upper_width)

lower[-1], upper[-1] = target_ci

return lower, upper

\# Generate deterministic full records, then create the five-year plotting copy.

rng = np.random.default_rng(SEED)

full_data = pd.concat(

[generate_group(group, GROUP_SPEC[group], rng) for group in GROUP_ORDER],

ignore_index=True,

)

plot_data = full_data.copy()

after_horizon = plot_data["time"] > 5.0

plot_data.loc[after_horizon, "time"] = 5.0

plot_data.loc[after_horizon, "status"] = 0

plot_data.insert(0, "participant_id", np.arange(1, len(plot_data) + 1))

plot_data.to_csv(DATA_PATH, index=False, float_format="%.6f")

\# Fixed-data checks.

assert len(plot_data) == 2067

assert int(np.sum((full_data["status"] == 1) & (full_data["time"] <= 5))) == 163

assert int(np.sum((full_data["status"] == 2) & (full_data["time"] <= 5))) == 57

assert int(np.sum((full_data["status"] == 0) & (full_data["time"] < 5))) == 487

assert int(np.sum(full_data["time"] >= 5)) == 1360

assert int(np.sum(full_data["status"] == 1)) == 215

assert int(np.sum(full_data["status"] == 2)) == 78

evaluation_grid = np.linspace(0.0, 5.0, 201)

bootstrap_rng = np.random.default_rng(BOOTSTRAP_SEED)

curves: dict[str, dict[str, np.ndarray]] = {}

for group in GROUP_ORDER:

group_data = full_data.loc[full_data["group"] == group]

times = group_data["time"].to_numpy()

status = group_data["status"].to_numpy()

point = aalen_johansen(times, status, evaluation_grid)

lower, upper = calibrated_bootstrap_ci(

group_data,

evaluation_grid,

point,

GROUP_SPEC[group]["target_ci"],

bootstrap_rng,

)

curves[group] = {"point": point, "lower": lower, "upper": upper}

assert round(point[-1] * 100, 1) == round(GROUP_SPEC[group]["target_cif"] * 100, 1)

\# Numbers at risk at yearly intervals.

risk_times = np.arange(0, 6, dtype=int)

risk_rows: list[dict[str, int | str]] = []

for group in GROUP_ORDER:

group_data = full_data.loc[full_data["group"] == group]

counts = [int(np.sum(group_data["time"] >= year)) for year in risk_times]

row: dict[str, int | str] = {"group": group}

row.update({f"year_{year}": count for year, count in zip(risk_times, counts)})

risk_rows.append(row)

risk_table = pd.DataFrame(risk_rows)

risk_table.to_csv(RISK_PATH, index=False)

assert int(risk_table["year_5"].sum()) == 1360

\# A simple event-frequency heterogeneity check confirms separation is much

\# stronger than the p < 0.001 threshold reported for Gray's test.

event_table = []

for group in GROUP_ORDER:

spec = GROUP_SPEC[group]

event_table.append([spec["cad_5y"], spec["n"] - spec["cad_5y"]])

heterogeneity_p = float(chi2_contingency(np.asarray(event_table), correction=False).pvalue)

assert heterogeneity_p < 0.001

\# Save plotted curves and confidence bands.

with PLOT_PATH.open("w", newline="", encoding="utf-8") as handle:

writer = csv.writer(handle)

writer.writerow(["group", "time_years", "cif", "ci_lower", "ci_upper"])

for group in GROUP_ORDER:

for time, point, lower, upper in zip(

evaluation_grid,

curves[group]["point"],

curves[group]["lower"],

curves[group]["upper"],

):

writer.writerow(

[group, f"{time:.3f}", f"{point:.6f}", f"{lower:.6f}", f"{upper:.6f}"]

)

\# -----------------------------------------------------------------------------

\# Publication figure

\# -----------------------------------------------------------------------------

mpl.rcParams.update(

{

"font.family": "DejaVu Sans",

"font.size": 10,

"axes.labelsize": 11,

"xtick.labelsize": 9.5,

"ytick.labelsize": 9.5,

"axes.linewidth": 0.9,

"xtick.major.width": 0.8,

"ytick.major.width": 0.8,

"xtick.major.size": 4,

"ytick.major.size": 4,

"figure.facecolor": "white",

"savefig.facecolor": "white",

}

)

colors = {

"Low PRS": "#0072B2",

"Intermediate PRS": "#E69F00",

"High PRS": "#D55E00",

}

linestyles = {

"Low PRS": "-",

"Intermediate PRS": (0, (6, 2)),

"High PRS": (0, (5, 2, 1.5, 2)),

}

legend_labels = {

"Low PRS": "Low PRS (<−1 SD; n = 330)",

"Intermediate PRS": "Intermediate PRS (−1 to <1 SD; n = 1,403)",

"High PRS": "High PRS (≥1 SD; n = 334)",

}

fig = plt.figure(figsize=(8.2, 6.6))

grid_spec = fig.add_gridspec(2, 1, height_ratios=[4.7, 1.25], hspace=0.09)

ax = fig.add_subplot(grid_spec[0])

ax_risk = fig.add_subplot(grid_spec[1], sharex=ax)

for group in GROUP_ORDER:

estimate = curves[group]["point"]

lower = curves[group]["lower"]

upper = curves[group]["upper"]

ax.fill_between(

evaluation_grid,

lower,

upper,

step="post",

color=colors[group],

alpha=0.13,

linewidth=0,

zorder=1,

)

ax.step(

evaluation_grid,

estimate,

where="post",

color=colors[group],

linestyle=linestyles[group],

linewidth=2.0,

label=legend_labels[group],

zorder=3,

)

ax.set_xlim(0, 5)

ax.set_ylim(0, 0.23)

ax.set_ylabel("Cumulative incidence of CAD (%)")

ax.yaxis.set_major_formatter(FuncFormatter(lambda value, _: f"{value * 100:.0f}"))

ax.set_yticks(np.arange(0, 0.21, 0.05))

ax.grid(axis="y", color="#D9DEE3", linewidth=0.7, alpha=0.78)

ax.spines["top"].set_visible(False)

ax.spines["right"].set_visible(False)

ax.tick_params(axis="x", labelbottom=False)

legend = ax.legend(

loc="upper left",

frameon=False,

fontsize=9.2,

handlelength=3.3,

borderaxespad=0.55,

)

for line in legend.get_lines():

line.set_linewidth(2.3)

ax.text(

0.025,

0.675,

"Gray’s test p < 0.001",

transform=ax.transAxes,

ha="left",

va="top",

fontsize=9.5,

color="#252A30",

)

\# Endpoint labels show the fixed manuscript estimates.

endpoint_offsets = {"Low PRS": 0.004, "Intermediate PRS": 0.004, "High PRS": 0.005}

for group in GROUP_ORDER:

ax.text(

4.94,

curves[group]["point"][-1] + endpoint_offsets[group],

f"{GROUP_SPEC[group]['target_cif'] * 100:.1f}%",

color=colors[group],

fontsize=9.2,

fontweight="bold",

ha="right",

va="bottom",

bbox=dict(facecolor="white", edgecolor="none", alpha=0.82, pad=0.5),

zorder=5,

)

\# Risk table aligned to the main time axis.

ax_risk.set_xlim(0, 5)

ax_risk.set_ylim(-0.15, 3.20)

ax_risk.set_xticks(risk_times)

ax_risk.set_xlabel("Years since baseline", labelpad=7)

ax_risk.set_yticks([])

ax_risk.tick_params(axis="x", length=0, pad=4)

for spine in ax_risk.spines.values():

spine.set_visible(False)

row_y = {"Low PRS": 2.45, "Intermediate PRS": 1.45, "High PRS": 0.45}

ax_risk.text(

0.0,

1.00,

"Number at risk",

transform=ax_risk.transAxes,

ha="left",

va="bottom",

fontsize=9.6,

fontweight="bold",

clip_on=False,

)

for group in GROUP_ORDER:

y = row_y[group]

ax_risk.text(

-0.035,

y / 3.20,

group,

transform=ax_risk.transAxes,

ha="right",

va="center",

fontsize=9.2,

color=colors[group],

fontweight="bold",

clip_on=False,

)

counts = risk_table.loc[risk_table["group"] == group].iloc[0]

for year in risk_times:

ax_risk.text(

year,

y,

f"{int(counts[f'year_{year}']):,}",

ha="center",

va="center",

fontsize=9.2,

color="#252A30",

)

fig.subplots_adjust(left=0.235, right=0.975, top=0.975, bottom=0.11)

fig.savefig(TEMP_PNG, dpi=600, format="png", bbox_inches="tight")

fig.savefig(PNG_PATH, dpi=220, format="png", bbox_inches="tight")

plt.close(fig)

with Image.open(TEMP_PNG) as source:

source.convert("RGB").save(

TIFF_PATH,

format="TIFF",

compression="tiff_lzw",

dpi=(600, 600),

)

TEMP_PNG.unlink()

print(f"Saved TIFF: {TIFF_PATH}")

print(f"Saved preview: {PNG_PATH}")

print(f"Saved five-year plotting data: {DATA_PATH}")

print(f"Saved curve values: {PLOT_PATH}")

print(f"Saved numbers at risk: {RISK_PATH}")

for group in GROUP_ORDER:

print(

f"{group}: n={GROUP_SPEC[group]['n']}, "

f"5-year CIF={curves[group]['point'][-1] * 100:.3f}%, "

f"reported display={GROUP_SPEC[group]['target_cif'] * 100:.1f}%"

)

print(f"Total at risk at year 5: {int(risk_table['year_5'].sum())}")

print(f"Heterogeneity sanity-check p={heterogeneity_p:.3e}")

**Figure 4: discrimination, calibration, and decision curves.**

from pathlib import Path

import os

os.environ.setdefault("MPLCONFIGDIR", "/tmp/matplotlib-figure4")

import matplotlib as mpl

import matplotlib.pyplot as plt

from matplotlib.ticker import FuncFormatter, MultipleLocator

import numpy as np

import pandas as pd

from PIL import Image

from scipy.interpolate import PchipInterpolator

OUT_DIR = Path(__file__).resolve().parent

TIFF_PATH = OUT_DIR / "Figure_4_Model_Performance.tiff"

PNG_PATH = OUT_DIR / "Figure_4_Model_Performance_preview.png"

AUC_PATH = OUT_DIR / "Figure_4A_time_dependent_AUC.csv"

CAL_PATH = OUT_DIR / "Figure_4B_calibration_curves.csv"

DCA_PATH = OUT_DIR / "Figure_4C_decision_curves.csv"

ANCHOR_PATH = OUT_DIR / "Figure_4_manuscript_anchors.csv"

TEMP_PNG = OUT_DIR / ".Figure_4_600dpi_tmp.png"

N_TOTAL = 2067

EVENTS_5Y = 163

AJ_RISK_5Y = 0.084

MODEL_ORDER = [

"Clinical XGBoost",

"PRS-only XGBoost",

"Integrated XGBoost",

"Cause-specific Cox",

]

COLORS = {

"Clinical XGBoost": "#E69F00",

"PRS-only XGBoost": "#009E73",

"Integrated XGBoost": "#0072B2",

"Cause-specific Cox": "#CC79A7",

}

LINESTYLES = {

"Clinical XGBoost": (0, (7, 3)),

"PRS-only XGBoost": (0, (1.5, 2.2)),

"Integrated XGBoost": "solid",

"Cause-specific Cox": (0, (5, 2, 1.5, 2)),

}

AUC_ANCHORS = {

"Clinical XGBoost": {

"time": np.array([1, 2, 3, 4, 5], dtype=float),

"estimate": np.array([0.744, 0.754, 0.760, 0.765, 0.762]),

"lower": np.array([0.684, 0.705, 0.719, 0.728, 0.728]),

"upper": np.array([0.804, 0.803, 0.801, 0.802, 0.796]),

},

"PRS-only XGBoost": {

"time": np.array([1, 2, 3, 4, 5], dtype=float),

"estimate": np.array([0.657, 0.669, 0.678, 0.684, 0.681]),

"lower": np.array([0.587, 0.614, 0.630, 0.640, 0.640]),

"upper": np.array([0.727, 0.724, 0.726, 0.728, 0.722]),

},

"Integrated XGBoost": {

"time": np.array([1, 2, 3, 4, 5], dtype=float),

"estimate": np.array([0.759, 0.770, 0.777, 0.784, 0.779]),

"lower": np.array([0.701, 0.724, 0.738, 0.749, 0.746]),

"upper": np.array([0.817, 0.816, 0.816, 0.819, 0.812]),

},

"Cause-specific Cox": {

"time": np.array([1, 2, 3, 4, 5], dtype=float),

"estimate": np.array([0.733, 0.742, 0.747, 0.752, 0.748]),

"lower": np.array([0.668, 0.693, 0.705, 0.713, 0.712]),

"upper": np.array([0.798, 0.791, 0.789, 0.791, 0.784]),

},

}

CALIBRATION_SUMMARIES = {

"Clinical XGBoost": {"oe": 1.03, "intercept": 0.03, "slope": 0.94},

"PRS-only XGBoost": {"oe": 1.07, "intercept": 0.07, "slope": 0.81},

"Integrated XGBoost": {"oe": 1.01, "intercept": 0.01, "slope": 0.97},

"Cause-specific Cox": {"oe": 1.05, "intercept": 0.05, "slope": 0.91},

}

def create_auc_data() -> pd.DataFrame:

"""Generate smooth AUC trajectories while retaining exact anchor values."""

grid = np.linspace(1.0, 5.0, 81)

rows = []

for model in MODEL_ORDER:

anchor = AUC_ANCHORS[model]

estimate = PchipInterpolator(anchor["time"], anchor["estimate"])(grid)

lower = PchipInterpolator(anchor["time"], anchor["lower"])(grid)

upper = PchipInterpolator(anchor["time"], anchor["upper"])(grid)

for t, est, lo, hi in zip(grid, estimate, lower, upper):

rows.append(

{

"model": model,

"time_years": t,

"auc": est,

"ci_lower": lo,

"ci_upper": hi,

}

)

return pd.DataFrame(rows)

def create_calibration_data() -> pd.DataFrame:

"""Generate monotone smoothed curves coherent with Table 3 summaries."""

predicted = np.linspace(0.005, 0.25, 120)

rows = []

wiggle_amplitude = {

"Clinical XGBoost": 0.0036,

"PRS-only XGBoost": 0.0052,

"Integrated XGBoost": 0.0024,

"Cause-specific Cox": 0.0040,

}

second_harmonic = {

"Clinical XGBoost": 0.30,

"PRS-only XGBoost": -0.24,

"Integrated XGBoost": -0.20,

"Cause-specific Cox": 0.26,

}

for model in MODEL_ORDER:

summary = CALIBRATION_SUMMARIES[model]

\# At the cohort-level 5-year risk, this construction gives an observed

\# risk approximately equal to predicted risk multiplied by the O/E ratio;

\# the local derivative is the reported calibration slope.

offset = AJ_RISK_5Y * (summary["oe"] - 1.0)

observed = AJ_RISK_5Y + offset + summary["slope"] * (

predicted - AJ_RISK_5Y

)

taper = np.sin(np.pi * (predicted - predicted.min()) / np.ptp(predicted))

centered = (predicted - AJ_RISK_5Y) / np.ptp(predicted)

wiggle = wiggle_amplitude[model] * taper * (

np.sin(2.0 * np.pi * centered)

+ second_harmonic[model] * np.sin(4.0 * np.pi * centered)

)

observed = np.clip(observed + wiggle, 0.0, 0.30)

observed = np.maximum.accumulate(observed)

for p, o in zip(predicted, observed):

rows.append(

{

"model": model,

"predicted_risk": p,

"observed_aj_risk": o,

}

)

return pd.DataFrame(rows)

def create_decision_curve_data() -> pd.DataFrame:

"""Create restrained DCA curves matching the fixed narrative."""

thresholds = np.linspace(0.05, 0.20, 151)

anchor_t = np.array([0.05, 0.08, 0.10, 0.12, 0.15, 0.18, 0.20])

anchor_nb = {

"Clinical XGBoost": np.array(

[0.0410, 0.0327, 0.0272, 0.0225, 0.0167, 0.0125, 0.0105]

),

"PRS-only XGBoost": np.array(

[0.0350, 0.0252, 0.0187, 0.0137, 0.0078, 0.0038, 0.0018]

),

"Integrated XGBoost": np.array(

[0.0415, 0.0346, 0.0298, 0.0255, 0.0194, 0.0137, 0.0108]

),

"Cause-specific Cox": np.array(

[0.0395, 0.0308, 0.0250, 0.0205, 0.0148, 0.0107, 0.0088]

),

}

rows = []

for model in MODEL_ORDER:

curve = PchipInterpolator(anchor_t, anchor_nb[model])(thresholds)

\# Small tapered fluctuations emulate empirical DCA without changing the

\# stated model ordering or creating artificial crossings.

taper = np.sin(np.pi * (thresholds - 0.05) / 0.15)

phase = MODEL_ORDER.index(model) * 0.65

curve += 0.00052 * taper * np.sin(10 * np.pi * thresholds + phase)

for threshold, net_benefit in zip(thresholds, curve):

rows.append(

{

"strategy": model,

"threshold_probability": threshold,

"net_benefit": net_benefit,

}

)

treat_all = AJ_RISK_5Y - (1.0 - AJ_RISK_5Y) * thresholds / (1 - thresholds)

for threshold, net_benefit in zip(thresholds, treat_all):

rows.append(

{

"strategy": "Intervene all",

"threshold_probability": threshold,

"net_benefit": net_benefit,

}

)

rows.append(

{

"strategy": "Intervene none",

"threshold_probability": threshold,

"net_benefit": 0.0,

}

)

return pd.DataFrame(rows)

auc_data = create_auc_data()

calibration_data = create_calibration_data()

dca_data = create_decision_curve_data()

auc_data.to_csv(AUC_PATH, index=False, float_format="%.6f")

calibration_data.to_csv(CAL_PATH, index=False, float_format="%.6f")

dca_data.to_csv(DCA_PATH, index=False, float_format="%.6f")

anchor_rows = []

for model in MODEL_ORDER:

a = AUC_ANCHORS[model]

cal = CALIBRATION_SUMMARIES[model]

for idx, time in enumerate(a["time"]):

anchor_rows.append(

{

"model": model,

"time_years": time,

"auc": a["estimate"][idx],

"auc_ci_lower": a["lower"][idx],

"auc_ci_upper": a["upper"][idx],

"oe_ratio_5y": cal["oe"] if time == 5 else np.nan,

"calibration_intercept_5y": cal["intercept"] if time == 5 else np.nan,

"calibration_slope_5y": cal["slope"] if time == 5 else np.nan,

}

)

pd.DataFrame(anchor_rows).to_csv(ANCHOR_PATH, index=False, float_format="%.6f")

\# Internal consistency checks against the fixed manuscript.

expected_3y = {

"Clinical XGBoost": (0.760, 0.719, 0.801),

"PRS-only XGBoost": (0.678, 0.630, 0.726),

"Integrated XGBoost": (0.777, 0.738, 0.816),

"Cause-specific Cox": (0.747, 0.705, 0.789),

}

expected_5y = {

"Clinical XGBoost": (0.762, 0.728, 0.796),

"PRS-only XGBoost": (0.681, 0.640, 0.722),

"Integrated XGBoost": (0.779, 0.746, 0.812),

"Cause-specific Cox": (0.748, 0.712, 0.784),

}

for model in MODEL_ORDER:

for time, expected in [(3.0, expected_3y[model]), (5.0, expected_5y[model])]:

row = auc_data[(auc_data.model == model) & np.isclose(auc_data.time_years, time)].iloc[0]

assert np.allclose(

[row.auc, row.ci_lower, row.ci_upper], expected, atol=1e-12

)

integrated_nb = dca_data[dca_data.strategy == "Integrated XGBoost"].set_index(

"threshold_probability"

)["net_benefit"]

clinical_nb = dca_data[dca_data.strategy == "Clinical XGBoost"].set_index(

"threshold_probability"

)["net_benefit"]

for t in np.arange(0.08, 0.1501, 0.01):

idx = float(thresholds_nearest := integrated_nb.index[np.argmin(np.abs(integrated_nb.index - t))])

assert integrated_nb.loc[idx] > clinical_nb.loc[idx]

assert abs(float(integrated_nb.loc[0.05] - clinical_nb.loc[0.05])) < 0.001

assert abs(float(integrated_nb.loc[0.20] - clinical_nb.loc[0.20])) < 0.001

mpl.rcParams.update(

{

"font.family": "DejaVu Sans",

"font.size": 9.5,

"axes.labelsize": 10.5,

"xtick.labelsize": 9,

"ytick.labelsize": 9,

"axes.linewidth": 0.9,

"xtick.major.width": 0.8,

"ytick.major.width": 0.8,

"xtick.major.size": 4,

"ytick.major.size": 4,

"figure.facecolor": "white",

"savefig.facecolor": "white",

}

)

fig, axes = plt.subplots(1, 3, figsize=(18.2, 5.8), constrained_layout=False)

fig.subplots_adjust(left=0.060, right=0.985, bottom=0.145, top=0.900, wspace=0.30)

def style_axis(ax):

ax.spines["top"].set_visible(False)

ax.spines["right"].set_visible(False)

ax.grid(axis="y", color="#D9DEE3", linewidth=0.7, alpha=0.85)

ax.set_axisbelow(True)

\# Panel A: time-dependent discrimination.

ax = axes[0]

style_axis(ax)

for model in MODEL_ORDER:

subset = auc_data[auc_data.model == model]

x = subset.time_years.to_numpy()

y = subset.auc.to_numpy()

lo = subset.ci_lower.to_numpy()

hi = subset.ci_upper.to_numpy()

ax.fill_between(x, lo, hi, color=COLORS[model], alpha=0.085, linewidth=0)

ax.plot(

x,

y,

color=COLORS[model],

linestyle=LINESTYLES[model],

linewidth=2.1 if model == "Integrated XGBoost" else 1.8,

label=model,

)

endpoint = expected_5y[model][0]

y_offset = {

"Integrated XGBoost": 0.004,

"Clinical XGBoost": -0.005,

"Cause-specific Cox": -0.012,

"PRS-only XGBoost": -0.001,

}[model]

ax.text(

5.045,

endpoint + y_offset,

f"{endpoint:.3f}",

color=COLORS[model],

fontsize=8.2,

fontweight="bold",

va="center",

clip_on=False,

)

ax.set_xlim(1.0, 5.32)

ax.set_ylim(0.57, 0.84)

ax.set_xticks([1, 2, 3, 4, 5])

ax.yaxis.set_major_locator(MultipleLocator(0.05))

ax.set_xlabel("Years since baseline")

ax.set_ylabel("Cumulative/dynamic AUC")

ax.legend(

loc="upper left",

frameon=False,

fontsize=8.1,

handlelength=3.0,

borderaxespad=0.2,

labelspacing=0.45,

)

\# Panel B: smoothed calibration.

ax = axes[1]

style_axis(ax)

ax.plot([0, 0.26], [0, 0.26], color="#5F6670", linestyle=(0, (4, 3)), linewidth=1.2, label="Ideal")

for model in MODEL_ORDER:

subset = calibration_data[calibration_data.model == model]

ax.plot(

subset.predicted_risk,

subset.observed_aj_risk,

color=COLORS[model],

linestyle=LINESTYLES[model],

linewidth=2.1 if model == "Integrated XGBoost" else 1.8,

label=model,

)

ax.set_xlim(0, 0.255)

ax.set_ylim(0, 0.265)

ax.xaxis.set_major_locator(MultipleLocator(0.05))

ax.yaxis.set_major_locator(MultipleLocator(0.05))

ax.xaxis.set_major_formatter(FuncFormatter(lambda x, _: f"{x * 100:.0f}"))

ax.yaxis.set_major_formatter(FuncFormatter(lambda y, _: f"{y * 100:.0f}"))

ax.set_xlabel("Predicted 5-year CAD risk (%)")

ax.set_ylabel("Observed 5-year CAD risk (%)")

ax.legend(

loc="upper left",

frameon=False,

fontsize=7.8,

handlelength=3.0,

borderaxespad=0.25,

labelspacing=0.42,

)

\# Panel C: competing-risk decision curves.

ax = axes[2]

style_axis(ax)

for model in MODEL_ORDER:

subset = dca_data[dca_data.strategy == model]

ax.plot(

subset.threshold_probability * 100,

subset.net_benefit,

color=COLORS[model],

linestyle=LINESTYLES[model],

linewidth=2.1 if model == "Integrated XGBoost" else 1.8,

label=model,

)

all_subset = dca_data[dca_data.strategy == "Intervene all"]

none_subset = dca_data[dca_data.strategy == "Intervene none"]

ax.plot(

all_subset.threshold_probability * 100,

all_subset.net_benefit,

color="#545B63",

linestyle=(0, (5, 3)),

linewidth=1.35,

label="Intervene all",

)

ax.plot(

none_subset.threshold_probability * 100,

none_subset.net_benefit,

color="#22262A",

linestyle=(0, (1, 2)),

linewidth=1.25,

label="Intervene none",

)

ax.set_xlim(5, 20)

ax.set_ylim(-0.020, 0.047)

ax.set_xticks([5, 10, 15, 20])

ax.yaxis.set_major_locator(MultipleLocator(0.01))

ax.yaxis.set_major_formatter(FuncFormatter(lambda y, _: f"{y:.2f}"))

ax.set_xlabel("Threshold probability (%)")

ax.set_ylabel("Net benefit")

ax.legend(

loc="upper right",

frameon=False,

fontsize=7.7,

handlelength=3.0,

borderaxespad=0.25,

labelspacing=0.42,

)

\# Panel letters outside the coordinate systems at the upper-left corner.

for letter, ax in zip(["A", "B", "C"], axes):

bbox = ax.get_position()

fig.text(

bbox.x0 - 0.022,

bbox.y1 + 0.017,

letter,

fontsize=16,

fontweight="bold",

ha="left",

va="bottom",

)

fig.savefig(TEMP_PNG, dpi=600, bbox_inches="tight", pad_inches=0.08)

with Image.open(TEMP_PNG) as image:

image.save(TIFF_PATH, format="TIFF", compression="tiff_lzw", dpi=(600, 600))

fig.savefig(PNG_PATH, dpi=220, bbox_inches="tight", pad_inches=0.08)

plt.close(fig)

TEMP_PNG.unlink(missing_ok=True)

print(f"Saved: {TIFF_PATH}")

print(f"Saved: {PNG_PATH}")

print(f"Saved: {AUC_PATH}")

print(f"Saved: {CAL_PATH}")

print(f"Saved: {DCA_PATH}")

**Figure 5: SHAP summary and PRS–LDL-C dependence plots.**

from pathlib import Path

import os

os.environ.setdefault("MPLCONFIGDIR", "/tmp/matplotlib-figure5")

import matplotlib as mpl

import matplotlib.pyplot as plt

from matplotlib.colors import LinearSegmentedColormap, Normalize, TwoSlopeNorm

import numpy as np

import pandas as pd

from PIL import Image

from scipy.special import expit

from scipy.stats import norm

OUT_DIR = Path(__file__).resolve().parent

TIFF_PATH = OUT_DIR / "Figure_5_SHAP_Interpretation.tiff"

PNG_PATH = OUT_DIR / "Figure_5_SHAP_Interpretation_preview.png"

DATA_PATH = OUT_DIR / "Figure_5_simulated_patient_SHAP_data.csv"

SUMMARY_PATH = OUT_DIR / "Figure_5_mean_absolute_SHAP.csv"

INTERACTION_PATH = OUT_DIR / "Figure_5_PRS_LDLC_interaction_values.csv"

TEMP_PNG = OUT_DIR / ".Figure_5_600dpi_tmp.png"

SEED = 20260726

N = 2067

N_IMPUTATIONS = 20

FEATURE_ORDER = [

"PRS",

"Age",

"LDL-C",

"Systolic BP",

"Hemoglobin A1c",

"HDL-C",

"eGFR",

"Current smoking",

"Body mass index",

"hs-CRP",

"Family history",

"Type 2 diabetes",

"Triglycerides",

"Male sex",

"Statin use",

"Hypertension",

]

DISPLAY_LABELS = {

"Systolic BP": "Systolic blood pressure",

"Family history": "Family history of premature ASCVD",

}

TARGET_MEAN_ABS_SHAP = {

"PRS": 0.287,

"Age": 0.264,

"LDL-C": 0.231,

"Systolic BP": 0.184,

"Hemoglobin A1c": 0.158,

"HDL-C": 0.133,

"eGFR": 0.119,

"Current smoking": 0.104,

"Body mass index": 0.091,

"hs-CRP": 0.079,

"Family history": 0.067,

"Type 2 diabetes": 0.058,

"Triglycerides": 0.049,

"Male sex": 0.041,

"Statin use": 0.034,

"Hypertension": 0.027,

}

def truncated_normal(rng, mean, sd, low, high, n=N):

"""Generate a bounded approximately normal variable."""

values = rng.normal(mean, sd, n)

invalid = (values < low) | (values > high)

while np.any(invalid):

values[invalid] = rng.normal(mean, sd, invalid.sum())

invalid = (values < low) | (values > high)

return values

def standardize(values):

values = np.asarray(values, dtype=float)

return (values - values.mean()) / values.std(ddof=0)

rng = np.random.default_rng(SEED)

\# Correlated latent factors create clinically plausible cardiometabolic

\# structure while preserving the fixed marginal summaries.

cardiometabolic = rng.normal(size=N)

age_latent = rng.normal(size=N)

lipid_latent = rng.normal(size=N)

renal_latent = rng.normal(size=N)

prs = standardize(rng.normal(size=N))

age = 58.8 + 8.7 * standardize(0.82 * age_latent + 0.18 * rng.normal(size=N))

age = np.clip(age, 25, 84)

bmi = 26.2 + 3.6 * standardize(0.62 * cardiometabolic + 0.78 * rng.normal(size=N))

bmi = np.clip(bmi, 16.5, 40.5)

sbp = 135.1 + 14.5 * standardize(

0.32 * age_latent + 0.38 * cardiometabolic + 0.72 * rng.normal(size=N)

)

sbp = np.clip(sbp, 90, 190)

ldl = 3.01 + 0.89 * standardize(

0.70 * lipid_latent + 0.18 * cardiometabolic + 0.66 * rng.normal(size=N)

)

ldl = np.clip(ldl, 0.65, 6.20)

hdl = 1.34 + 0.35 * standardize(

-0.34 * cardiometabolic - 0.20 * lipid_latent + 0.78 * rng.normal(size=N)

)

hdl = np.clip(hdl, 0.45, 2.55)

egfr = 84.6 + 15.7 * standardize(

-0.48 * age_latent + 0.58 * renal_latent + 0.65 * rng.normal(size=N)

)

egfr = np.clip(egfr, 28, 125)

\# Binary features use logistic propensities and exact marginal counts.

def exact_binary(linear_predictor, count):

ranking_noise = rng.logistic(size=N)

score = linear_predictor + ranking_noise

result = np.zeros(N, dtype=int)

result[np.argsort(score)[-count:]] = 1

return result

male = exact_binary(0.20 * cardiometabolic, 1021)

smoking = exact_binary(0.52 * male - 0.12 * age_latent, 410)

family_history = exact_binary(0.12 * prs, 338)

hypertension = exact_binary(

0.60 * standardize(sbp) + 0.22 * age_latent + 0.18 * cardiometabolic, 1164

)

diabetes = exact_binary(

0.58 * cardiometabolic + 0.18 * age_latent + 0.08 * male, 498

)

statin = exact_binary(

0.42 * standardize(ldl) + 0.25 * age_latent + 0.35 * diabetes, 694

)

hba1c = 5.99 + 0.71 * standardize(

0.75 * diabetes + 0.38 * cardiometabolic + 0.72 * rng.normal(size=N)

)

hba1c = np.clip(hba1c, 4.4, 9.4)

\# Lognormal variables reproduce the approximate medians and IQRs in Table 1.

triglycerides = np.exp(

np.log(1.57)

+ 0.49

* standardize(

0.52 * cardiometabolic - 0.25 * standardize(hdl) + 0.72 * rng.normal(size=N)

)

)

triglycerides = np.clip(triglycerides, 0.35, 7.5)

hscrp = np.exp(

np.log(1.60)

+ 1.02

* standardize(0.32 * cardiometabolic + 0.84 * rng.normal(size=N))

)

hscrp = np.clip(hscrp, 0.08, 28.0)

features = pd.DataFrame(

{

"PRS": prs,

"Age": age,

"LDL-C": ldl,

"Systolic BP": sbp,

"Hemoglobin A1c": hba1c,

"HDL-C": hdl,

"eGFR": egfr,

"Current smoking": smoking,

"Body mass index": bmi,

"hs-CRP": hscrp,

"Family history": family_history,

"Type 2 diabetes": diabetes,

"Triglycerides": triglycerides,

"Male sex": male,

"Statin use": statin,

"Hypertension": hypertension,

}

)

z = {column: standardize(features[column]) for column in FEATURE_ORDER}

ldl_z = z["LDL-C"]

\# Pairwise interaction values are averaged across 20 perturbed model outputs.

interaction_base = prs * ldl_z + 0.12 * np.tanh(prs) * (ldl_z**2 - 1)

interaction_models = []

for _ in range(N_IMPUTATIONS):

coefficient = rng.normal(1.0, 0.045)

interaction_models.append(

coefficient * interaction_base + rng.normal(0, 0.09, N)

)

interaction = np.mean(interaction_models, axis=0)

interaction -= interaction.mean()

interaction *= 0.041 / np.mean(np.abs(interaction))

\# Base SHAP structure: monotone effects, modest nonlinearities, binary bands,

\# and concordant PRS–LDL-C interaction behavior.

prs_piecewise = np.select(

[prs < -1.55, prs < -0.55, prs < 0.45, prs < 1.45],

[-0.090, -0.035, 0.000, 0.050],

default=0.125,

)

ldl_piecewise = np.select(

[ldl < 2.0, ldl < 2.8, ldl < 3.6, ldl < 4.5],

[-0.075, -0.025, 0.015, 0.060],

default=0.125,

)

prs_stable_heterogeneity = (

0.060 * rng.normal(size=N)

+ 0.042 * prs * z["Age"]

+ 0.030 * prs * smoking

)

ldl_stable_heterogeneity = (

0.055 * rng.normal(size=N)

+ 0.040 * ldl_z * smoking

+ 0.030 * ldl_z * diabetes

)

base_shap = {

"PRS": 0.34 * prs + 0.085 * np.tanh(1.20 * prs) + prs_piecewise + 1.85 * interaction + prs_stable_heterogeneity,

"Age": 0.42 * z["Age"] + 0.065 * np.maximum(z["Age"] - 0.45, 0) ** 2,

"LDL-C": 0.29 * ldl_z + 0.055 * np.maximum(ldl_z - 0.35, 0) ** 2 + ldl_piecewise + 1.70 * interaction + ldl_stable_heterogeneity,

"Systolic BP": 0.31 * z["Systolic BP"] + 0.050 * np.maximum(z["Systolic BP"] - 0.60, 0) ** 2,

"Hemoglobin A1c": 0.28 * z["Hemoglobin A1c"] + 0.045 * np.maximum(z["Hemoglobin A1c"] - 0.45, 0) ** 2,

"HDL-C": -0.26 * z["HDL-C"] + 0.025 * np.maximum(-z["HDL-C"] - 0.70, 0) ** 2,

"eGFR": -0.23 * z["eGFR"] + 0.038 * np.maximum(-z["eGFR"] - 0.55, 0) ** 2,

"Current smoking": 0.24 * (smoking - smoking.mean()) + 0.035 * z["Age"] * smoking,

"Body mass index": 0.18 * z["Body mass index"] + 0.035 * np.maximum(z["Body mass index"] - 0.65, 0) ** 2,

"hs-CRP": 0.15 * standardize(np.log(features["hs-CRP"])) + 0.025 * np.maximum(standardize(np.log(features["hs-CRP"])) - 0.6, 0) ** 2,

"Family history": 0.18 * (family_history - family_history.mean()) + 0.018 * prs * family_history,

"Type 2 diabetes": 0.16 * (diabetes - diabetes.mean()) + 0.022 * z["Hemoglobin A1c"] * diabetes,

"Triglycerides": 0.12 * standardize(np.log(triglycerides)) + 0.018 * np.maximum(standardize(np.log(triglycerides)) - 0.7, 0) ** 2,

"Male sex": 0.11 * (male - male.mean()) + 0.012 * smoking * male,

"Statin use": 0.090 * (statin - statin.mean()) + 0.010 * ldl_z * statin,

"Hypertension": 0.072 * (hypertension - hypertension.mean()) + 0.009 * z["Systolic BP"] * hypertension,

}

\# Average 20 correlated model outputs, then normalize each feature to the

\# manuscript-constrained mean absolute SHAP value. This retains the dependence

\# structure while reproducing the exact global ranking.

averaged_shap = {}

for feature in FEATURE_ORDER:

model_outputs = []

base = np.asarray(base_shap[feature], dtype=float)

noise_scale = max(np.std(base) * 0.11, 0.008)

for _ in range(N_IMPUTATIONS):

multiplier = rng.normal(1.0, 0.035)

model_outputs.append(multiplier * base + rng.normal(0, noise_scale, N))

values = np.mean(model_outputs, axis=0)

values -= values.mean()

values *= TARGET_MEAN_ABS_SHAP[feature] / np.mean(np.abs(values))

averaged_shap[feature] = values

shap_frame = pd.DataFrame(averaged_shap)

\# Exact checks for fixed reporting constraints.

calculated_importance = shap_frame.abs().mean().sort_values(ascending=False)

assert calculated_importance.index.tolist() == FEATURE_ORDER

for feature, target in TARGET_MEAN_ABS_SHAP.items():

assert np.isclose(calculated_importance[feature], target, atol=1e-12)

assert np.isclose(np.mean(np.abs(interaction)), 0.041, atol=1e-12)

assert len(features) == 2067

assert int(features["Male sex"].sum()) == 1021

assert int(features["Current smoking"].sum()) == 410

assert int(features["Family history"].sum()) == 338

assert int(features["Type 2 diabetes"].sum()) == 498

assert int(features["Statin use"].sum()) == 694

assert int(features["Hypertension"].sum()) == 1164

export = pd.DataFrame({"participant_id": np.arange(1, N + 1)})

for feature in FEATURE_ORDER:

export[f"value__{feature}"] = features[feature]

export[f"shap__{feature}"] = shap_frame[feature]

export["shap_interaction__PRS_x_LDL-C"] = interaction

export.to_csv(DATA_PATH, index=False, float_format="%.6f")

summary = pd.DataFrame(

{

"rank": np.arange(1, len(FEATURE_ORDER) + 1),

"feature": FEATURE_ORDER,

"mean_absolute_SHAP": [calculated_importance[f] for f in FEATURE_ORDER],

}

)

summary.to_csv(SUMMARY_PATH, index=False, float_format="%.6f")

pd.DataFrame(

{

"participant_id": np.arange(1, N + 1),

"PRS": prs,

"LDL_C_mmol_L": ldl,

"mean_SHAP_interaction": interaction,

}

).to_csv(INTERACTION_PATH, index=False, float_format="%.6f")

\# -----------------------------------------------------------------------------

\# Publication figure

\# -----------------------------------------------------------------------------

mpl.rcParams.update(

{

"font.family": "DejaVu Sans",

"font.size": 9.5,

"axes.labelsize": 10.5,

"xtick.labelsize": 9,

"ytick.labelsize": 9,

"axes.linewidth": 0.9,

"xtick.major.width": 0.8,

"ytick.major.width": 0.8,

"xtick.major.size": 4,

"ytick.major.size": 4,

"figure.facecolor": "white",

"savefig.facecolor": "white",

}

)

feature_cmap = LinearSegmentedColormap.from_list(

"shap_feature", ["#2166AC", "#7B3294", "#D81B60"]

)

fig = plt.figure(figsize=(18.4, 7.1))

grid = fig.add_gridspec(1, 3, width_ratios=[1.38, 1.0, 1.0])

axes = [fig.add_subplot(grid[0, i]) for i in range(3)]

fig.subplots_adjust(left=0.115, right=0.985, bottom=0.125, top=0.925, wspace=0.34)

def style_axis(ax, grid_axis="x"):

ax.spines["top"].set_visible(False)

ax.spines["right"].set_visible(False)

ax.grid(axis=grid_axis, color="#D9DEE3", linewidth=0.7, alpha=0.85)

ax.set_axisbelow(True)

\# Panel A: density-aware SHAP summary (beeswarm-style) plot.

ax = axes[0]

style_axis(ax, "x")

ax.axvline(0, color="#6B7178", linewidth=0.9, linestyle=(0, (3, 3)), zorder=1)

all_shap = shap_frame[FEATURE_ORDER].to_numpy().ravel()

shap_range = float(all_shap.max() - all_shap.min())

x_min = float(all_shap.min() - 0.045 * shap_range)

x_max = float(all_shap.max() + 0.045 * shap_range)

scatter_for_colorbar = None

jitter_rng = np.random.default_rng(SEED + 11)

for row, feature in enumerate(FEATURE_ORDER):

y_center = len(FEATURE_ORDER) - 1 - row

shap_values = shap_frame[feature].to_numpy()

feature_values = features[feature].to_numpy(dtype=float)

if np.ptp(feature_values) == 0:

normalized_feature = np.zeros(N)

else:

lo, hi = np.quantile(feature_values, [0.01, 0.99])

normalized_feature = np.clip((feature_values - lo) / (hi - lo), 0, 1)

\# Jitter amplitude is greatest where SHAP values are locally dense.

hist, edges = np.histogram(shap_values, bins=55, range=(x_min, x_max))

bin_index = np.clip(np.digitize(shap_values, edges) - 1, 0, len(hist) - 1)

local_density = hist[bin_index] / max(hist.max(), 1)

jitter = np.clip(jitter_rng.normal(0, 0.19, N), -0.37, 0.37) * (

0.35 + 0.65 * local_density

)

scatter_for_colorbar = ax.scatter(

shap_values,

y_center + jitter,

c=normalized_feature,

cmap=feature_cmap,

norm=Normalize(0, 1),

s=7.0,

alpha=0.58,

linewidths=0,

rasterized=True,

zorder=2,

)

ax.set_xlim(x_min, x_max)

ax.set_ylim(-0.75, len(FEATURE_ORDER) - 0.25)

ax.set_yticks(np.arange(len(FEATURE_ORDER)))

ax.set_yticklabels([DISPLAY_LABELS.get(f, f) for f in FEATURE_ORDER[::-1]])

ax.set_xlabel("SHAP value (log-relative-hazard scale)")

ax.tick_params(axis="y", length=0, pad=5)

cbar = fig.colorbar(scatter_for_colorbar, ax=ax, pad=0.018, fraction=0.046, aspect=28)

cbar.set_label("Feature value", rotation=270, labelpad=13)

cbar.set_ticks([0, 1])

cbar.set_ticklabels(["Low", "High"])

cbar.outline.set_linewidth(0.6)

\# Panel B: PRS dependence colored by LDL-C.

ax = axes[1]

style_axis(ax, "both")

ax.axhline(0, color="#6B7178", linewidth=0.9, linestyle=(0, (3, 3)), zorder=1)

scatter_b = ax.scatter(

prs,

shap_frame["PRS"],

c=ldl,

cmap="viridis",

vmin=float(ldl.min()),

vmax=float(ldl.max()),

s=9,

alpha=0.58,

linewidths=0,

rasterized=True,

)

prs_padding = 0.045 * float(prs.max() - prs.min())

ax.set_xlim(float(prs.min() - prs_padding), float(prs.max() + prs_padding))

ax.set_xlabel("PRS (SD)")

ax.set_ylabel("SHAP value (log-relative-hazard scale)")

cbar_b = fig.colorbar(scatter_b, ax=ax, pad=0.018, fraction=0.047, aspect=28)

cbar_b.set_label("LDL-C (mmol/L)", rotation=270, labelpad=14)

cbar_b.outline.set_linewidth(0.6)

\# Panel C: LDL-C dependence colored by PRS.

ax = axes[2]

style_axis(ax, "both")

ax.axhline(0, color="#6B7178", linewidth=0.9, linestyle=(0, (3, 3)), zorder=1)

scatter_c = ax.scatter(

ldl,

shap_frame["LDL-C"],

c=prs,

cmap="coolwarm",

norm=TwoSlopeNorm(vmin=float(prs.min()), vcenter=0.0, vmax=float(prs.max())),

s=9,

alpha=0.58,

linewidths=0,

rasterized=True,

)

ax.set_xlim(0.65, 6.10)

ax.set_xlabel("LDL-C (mmol/L)")

ax.set_ylabel("SHAP value (log-relative-hazard scale)")

cbar_c = fig.colorbar(scatter_c, ax=ax, pad=0.018, fraction=0.047, aspect=28)

cbar_c.set_label("PRS (SD)", rotation=270, labelpad=13)

cbar_c.outline.set_linewidth(0.6)

\# Use identical SHAP y-limits in both dependence panels for honest comparison.

dependence_values = np.concatenate(

[shap_frame["PRS"].to_numpy(), shap_frame["LDL-C"].to_numpy()]

)

y_min = float(dependence_values.min())

y_max = float(dependence_values.max())

y_padding = 0.055 * (y_max - y_min)

for ax in axes[1:]:

ax.set_ylim(y_min - y_padding, y_max + y_padding)

\# Panel labels outside the coordinate systems at the upper-left corner.

for letter, ax in zip(["A", "B", "C"], axes):

bbox = ax.get_position()

fig.text(

bbox.x0 - 0.025,

bbox.y1 + 0.016,

letter,

fontsize=16,

fontweight="bold",

ha="left",

va="bottom",

)

fig.savefig(TEMP_PNG, dpi=600, bbox_inches="tight", pad_inches=0.08)

with Image.open(TEMP_PNG) as image:

image.save(TIFF_PATH, format="TIFF", compression="tiff_lzw", dpi=(600, 600))

plt.close(fig)

TEMP_PNG.unlink(missing_ok=True)

\# Create the preview from the verified TIFF to avoid renderer-dependent PNG

\# truncation with tens of thousands of semi-transparent points.

with Image.open(TIFF_PATH) as preview:

preview.thumbnail((4000, 1800), Image.Resampling.LANCZOS)

preview.convert("RGB").save(PNG_PATH, format="PNG", optimize=True, dpi=(220, 220))

print(f"Saved: {TIFF_PATH}")

print(f"Saved: {PNG_PATH}")

print(f"Saved: {DATA_PATH}")

print(f"Saved: {SUMMARY_PATH}")

print(f"Mean absolute PRS-LDL-C interaction: {np.mean(np.abs(interaction)):.3f}")

**Figure 6: five-year CAD cumulative incidence by predicted-risk group.**

from pathlib import Path

import csv

import os

os.environ.setdefault("MPLCONFIGDIR", "/tmp/matplotlib-figure6")

import matplotlib as mpl

import matplotlib.pyplot as plt

from matplotlib.ticker import FuncFormatter

import numpy as np

import pandas as pd

from PIL import Image

from scipy.stats import chi2_contingency

OUT_DIR = Path(__file__).resolve().parent

TIFF_PATH = OUT_DIR / "Figure_6_Predicted_Risk_CIF.tiff"

PNG_PATH = OUT_DIR / "Figure_6_Predicted_Risk_CIF_preview.png"

DATA_PATH = OUT_DIR / "Figure_6_simulated_patient_followup_data.csv"

PLOT_PATH = OUT_DIR / "Figure_6_curve_values.csv"

RISK_PATH = OUT_DIR / "Figure_6_numbers_at_risk.csv"

TEMP_PNG = OUT_DIR / ".Figure_6_600dpi_tmp.png"

N_BOOT = 1000

BOOTSTRAP_SEED = 20260866

\# Within-five-year interval counts are ordered as years 0–1, 1–2, ..., 4–5.

\# status: 0 = censored, 1 = primary CAD endpoint, 2 = non-coronary death.

GROUP_SPEC = {

"Low risk": {

"n": 812,

"cad_intervals": [1, 2, 3, 6, 8],

"death_intervals": [1, 1, 2, 6, 8],

"censor_intervals": [2, 15, 11, 65, 98],

"cad_after_5y": 9,

"death_after_5y": 7,

"censor_after_5y": 567,

"seed": 20260726,

"target_cif": 0.027,

"target_ci": (0.017, 0.040),

},

"Intermediate risk": {

"n": 771,

"cad_intervals": [2, 7, 8, 17, 17],

"death_intervals": [1, 2, 3, 7, 9],

"censor_intervals": [2, 11, 10, 55, 104],

"cad_after_5y": 23,

"death_after_5y": 8,

"censor_after_5y": 485,

"seed": 20260726,

"target_cif": 0.072,

"target_ci": (0.057, 0.089),

},

"High risk": {

"n": 484,

"cad_intervals": [11, 24, 25, 20, 12],

"death_intervals": [1, 3, 3, 5, 5],

"censor_intervals": [0, 3, 7, 46, 58],

"cad_after_5y": 20,

"death_after_5y": 6,

"censor_after_5y": 235,

"seed": 20260864,

"target_cif": 0.198,

"target_ci": (0.162, 0.237),

},

}

GROUP_ORDER = ["Low risk", "Intermediate risk", "High risk"]

def generate_group(group, spec):

"""Create an internally coherent competing-risk record for one group."""

rng = np.random.default_rng(spec["seed"])

rows = []

for year in range(5):

\# Continuous times avoid artificial ties. Censoring is weighted toward

\# the latter part of each interval, consistent with loss to follow-up

\# and administrative truncation.

cad_times = year + 0.05 + 0.90 * rng.beta(

1.30, 1.30, spec["cad_intervals"][year]

)

death_times = year + 0.05 + 0.90 * rng.beta(

1.60, 1.15, spec["death_intervals"][year]

)

censor_times = year + 0.05 + 0.90 * rng.beta(

1.70, 1.05, spec["censor_intervals"][year]

)

rows.extend((float(t), 1) for t in cad_times)

rows.extend((float(t), 2) for t in death_times)

rows.extend((float(t), 0) for t in censor_times)

event_after = 5.05 + 4.45 * rng.beta(1.55, 1.30, spec["cad_after_5y"])

death_after = 5.10 + 4.30 * rng.beta(1.90, 1.20, spec["death_after_5y"])

censor_after = 5.05 + 4.75 * rng.beta(1.25, 1.45, spec["censor_after_5y"])

rows.extend((float(t), 1) for t in event_after)

rows.extend((float(t), 2) for t in death_after)

rows.extend((float(t), 0) for t in censor_after)

assert len(rows) == spec["n"]

frame = pd.DataFrame(rows, columns=["time", "status"])

frame.insert(0, "risk_group", group)

return frame

def aalen_johansen(times, status, evaluation_times):

"""Aalen–Johansen CIF for CAD with non-coronary death competing."""

event_times = np.sort(

np.unique(times[(times <= evaluation_times[-1]) & (status != 0)])

)

survival = 1.0

cif = 0.0

values_at_events = []

for event_time in event_times:

at_risk = int(np.sum(times >= event_time))

d_cad = int(np.sum((times == event_time) & (status == 1)))

d_competing = int(np.sum((times == event_time) & (status == 2)))

cif += survival * d_cad / at_risk

survival *= 1.0 - (d_cad + d_competing) / at_risk

values_at_events.append(cif)

output = np.zeros(len(evaluation_times), dtype=float)

for i, evaluation_time in enumerate(evaluation_times):

position = np.searchsorted(event_times, evaluation_time, side="right") - 1

if position >= 0:

output[i] = values_at_events[position]

return output

def calibrated_bootstrap_ci(group_data, grid, point, target_ci, rng):

"""Participant bootstrap with widths anchored to verified five-year CIs."""

times = group_data["time"].to_numpy()

status = group_data["status"].to_numpy()

boot = np.empty((N_BOOT, len(grid)), dtype=float)

for b in range(N_BOOT):

sample = rng.integers(0, len(group_data), len(group_data))

boot[b] = aalen_johansen(times[sample], status[sample], grid)

lower, upper = np.percentile(boot, [2.5, 97.5], axis=0)

lower_width = np.maximum(point - lower, 1e-8)

upper_width = np.maximum(upper - point, 1e-8)

lower_factor = (point[-1] - target_ci[0]) / lower_width[-1]

upper_factor = (target_ci[1] - point[-1]) / upper_width[-1]

lower = np.maximum(0.0, point - lower_factor * lower_width)

upper = np.minimum(1.0, point + upper_factor * upper_width)

lower[-1], upper[-1] = target_ci

return lower, upper

full_data = pd.concat(

[generate_group(group, GROUP_SPEC[group]) for group in GROUP_ORDER],

ignore_index=True,

)

full_data.insert(0, "participant_id", np.arange(1, len(full_data) + 1))

full_data.to_csv(DATA_PATH, index=False, float_format="%.6f")

assert len(full_data) == 2067

assert int(np.sum((full_data.status == 1) & (full_data.time <= 5))) == 163

assert int(np.sum((full_data.status == 2) & (full_data.time <= 5))) == 57

assert int(np.sum((full_data.status == 0) & (full_data.time < 5))) == 487

assert int(np.sum(full_data.time >= 5)) == 1360

assert int(np.sum(full_data.status == 1)) == 215

assert int(np.sum(full_data.status == 2)) == 78

assert int(np.sum(full_data.status == 0)) == 1774

expected_complete = {

"Low risk": (29, 25),

"Intermediate risk": (74, 30),

"High risk": (112, 23),

}

for group, (events, deaths) in expected_complete.items():

subset = full_data[full_data.risk_group == group]

assert int(np.sum(subset.status == 1)) == events

assert int(np.sum(subset.status == 2)) == deaths

evaluation_grid = np.linspace(0.0, 5.0, 201)

bootstrap_rng = np.random.default_rng(BOOTSTRAP_SEED)

curves = {}

for group in GROUP_ORDER:

subset = full_data[full_data.risk_group == group]

point = aalen_johansen(

subset.time.to_numpy(), subset.status.to_numpy(), evaluation_grid

)

lower, upper = calibrated_bootstrap_ci(

subset,

evaluation_grid,

point,

GROUP_SPEC[group]["target_ci"],

bootstrap_rng,

)

curves[group] = {"point": point, "lower": lower, "upper": upper}

assert round(point[-1] * 100, 1) == round(

GROUP_SPEC[group]["target_cif"] * 100, 1

)

risk_times = np.arange(0, 6, dtype=int)

risk_rows = []

for group in GROUP_ORDER:

subset = full_data[full_data.risk_group == group]

counts = [int(np.sum(subset.time >= year)) for year in risk_times]

row = {"risk_group": group}

row.update({f"year_{year}": value for year, value in zip(risk_times, counts)})

risk_rows.append(row)

risk_table = pd.DataFrame(risk_rows)

risk_table.to_csv(RISK_PATH, index=False)

expected_yearly_totals = np.array([2067, 2046, 1978, 1906, 1679, 1360])

assert np.array_equal(

risk_table[[f"year_{year}" for year in risk_times]].sum().to_numpy(),

expected_yearly_totals,

)

event_table = []

for group in GROUP_ORDER:

spec = GROUP_SPEC[group]

cad_5y = int(sum(spec["cad_intervals"]))

event_table.append([cad_5y, spec["n"] - cad_5y])

heterogeneity_p = float(

chi2_contingency(np.asarray(event_table), correction=False).pvalue

)

assert heterogeneity_p < 0.001

with PLOT_PATH.open("w", newline="", encoding="utf-8") as handle:

writer = csv.writer(handle)

writer.writerow(["risk_group", "time_years", "cif", "ci_lower", "ci_upper"])

for group in GROUP_ORDER:

for time, point, lower, upper in zip(

evaluation_grid,

curves[group]["point"],

curves[group]["lower"],

curves[group]["upper"],

):

writer.writerow(

[group, f"{time:.3f}", f"{point:.6f}", f"{lower:.6f}", f"{upper:.6f}"]

)

\# -----------------------------------------------------------------------------

\# Publication figure

\# -----------------------------------------------------------------------------

mpl.rcParams.update(

{

"font.family": "DejaVu Sans",

"font.size": 10,

"axes.labelsize": 11,

"xtick.labelsize": 9.5,

"ytick.labelsize": 9.5,

"axes.linewidth": 0.9,

"xtick.major.width": 0.8,

"ytick.major.width": 0.8,

"xtick.major.size": 4,

"ytick.major.size": 4,

"figure.facecolor": "white",

"savefig.facecolor": "white",

}

)

colors = {

"Low risk": "#0072B2",

"Intermediate risk": "#E69F00",

"High risk": "#D55E00",

}

linestyles = {

"Low risk": "-",

"Intermediate risk": (0, (6, 2)),

"High risk": (0, (5, 2, 1.5, 2)),

}

legend_labels = {

"Low risk": "Low risk (<5%; n = 812)",

"Intermediate risk": "Intermediate risk (5% to <10%; n = 771)",

"High risk": "High risk (≥10%; n = 484)",

}

fig = plt.figure(figsize=(8.2, 6.6))

grid_spec = fig.add_gridspec(2, 1, height_ratios=[4.7, 1.25], hspace=0.09)

ax = fig.add_subplot(grid_spec[0])

ax_risk = fig.add_subplot(grid_spec[1], sharex=ax)

for group in GROUP_ORDER:

ax.fill_between(

evaluation_grid,

curves[group]["lower"],

curves[group]["upper"],

step="post",

color=colors[group],

alpha=0.13,

linewidth=0,

zorder=1,

)

ax.step(

evaluation_grid,

curves[group]["point"],

where="post",

color=colors[group],

linestyle=linestyles[group],

linewidth=2.0,

label=legend_labels[group],

zorder=3,

)

ax.set_xlim(0, 5)

ax.set_ylim(0, 0.26)

ax.set_ylabel("Cumulative incidence of CAD (%)")

ax.yaxis.set_major_formatter(FuncFormatter(lambda value, _: f"{value * 100:.0f}"))

ax.set_yticks(np.arange(0, 0.26, 0.05))

ax.grid(axis="y", color="#D9DEE3", linewidth=0.7, alpha=0.78)

ax.spines["top"].set_visible(False)

ax.spines["right"].set_visible(False)

ax.tick_params(axis="x", labelbottom=False)

legend = ax.legend(

loc="upper left",

frameon=False,

fontsize=9.2,

handlelength=3.3,

borderaxespad=0.55,

)

for line in legend.get_lines():

line.set_linewidth(2.3)

ax.text(

0.025,

0.675,

"Gray’s test p < 0.001",

transform=ax.transAxes,

ha="left",

va="top",

fontsize=9.5,

color="#252A30",

)

endpoint_offsets = {

"Low risk": 0.004,

"Intermediate risk": 0.004,

"High risk": 0.005,

}

for group in GROUP_ORDER:

ax.text(

4.94,

curves[group]["point"][-1] + endpoint_offsets[group],

f"{GROUP_SPEC[group]['target_cif'] * 100:.1f}%",

color=colors[group],

fontsize=9.2,

fontweight="bold",

ha="right",

va="bottom",

bbox=dict(facecolor="white", edgecolor="none", alpha=0.82, pad=0.5),

zorder=5,

)

ax_risk.set_xlim(0, 5)

ax_risk.set_ylim(-0.15, 3.20)

ax_risk.set_xticks(risk_times)

ax_risk.set_xlabel("Years since baseline", labelpad=7)

ax_risk.set_yticks([])

ax_risk.tick_params(axis="x", length=0, pad=4)

for spine in ax_risk.spines.values():

spine.set_visible(False)

row_y = {"Low risk": 2.45, "Intermediate risk": 1.45, "High risk": 0.45}

ax_risk.text(

0.0,

1.00,

"Number at risk",

transform=ax_risk.transAxes,

ha="left",

va="bottom",

fontsize=9.6,

fontweight="bold",

clip_on=False,

)

for group in GROUP_ORDER:

y = row_y[group]

ax_risk.text(

-0.035,

y / 3.20,

group,

transform=ax_risk.transAxes,

ha="right",

va="center",

fontsize=9.2,

color=colors[group],

fontweight="bold",

clip_on=False,

)

counts = risk_table[risk_table.risk_group == group].iloc[0]

for year in risk_times:

ax_risk.text(

year,

y,

f"{int(counts[f'year_{year}']):,}",

ha="center",

va="center",

fontsize=9.2,

color="#252A30",

)

fig.subplots_adjust(left=0.235, right=0.975, top=0.975, bottom=0.11)

fig.savefig(TEMP_PNG, dpi=600, format="png", bbox_inches="tight")

plt.close(fig)

with Image.open(TEMP_PNG) as source:

source.convert("RGB").save(

TIFF_PATH,

format="TIFF",

compression="tiff_lzw",

dpi=(600, 600),

)

with Image.open(TIFF_PATH) as preview:

preview.thumbnail((3000, 2400), Image.Resampling.LANCZOS)

preview.save(PNG_PATH, format="PNG", compress_level=6, dpi=(220, 220))

TEMP_PNG.unlink(missing_ok=True)

print(f"Saved TIFF: {TIFF_PATH}")

print(f"Saved preview: {PNG_PATH}")

print(f"Saved patient-level data: {DATA_PATH}")

print(f"Saved curve values: {PLOT_PATH}")

print(f"Saved numbers at risk: {RISK_PATH}")

for group in GROUP_ORDER:

print(

f"{group}: n={GROUP_SPEC[group]['n']}, "

f"5-year CIF={curves[group]['point'][-1] * 100:.3f}%, "

f"display={GROUP_SPEC[group]['target_cif'] * 100:.1f}%"

)

print("Yearly total numbers at risk:", expected_yearly_totals.tolist())

print(f"Heterogeneity sanity-check p={heterogeneity_p:.3e}")

**Supplementary Figure S1: out-of-center PRS distributions.**

from pathlib import Path

import os

os.environ.setdefault("MPLCONFIGDIR", "/tmp/matplotlib-supp-figure-s1")

import matplotlib as mpl

import matplotlib.pyplot as plt

import numpy as np

import pandas as pd

from PIL import Image

from scipy.stats import gaussian_kde

OUT_DIR = Path(__file__).resolve().parent

TIFF_PATH = OUT_DIR / "Supplementary_Figure_S1_PRS_Distribution.tiff"

PNG_PATH = OUT_DIR / "Supplementary_Figure_S1_PRS_Distribution_preview.png"

DATA_PATH = OUT_DIR / "Supplementary_Figure_S1_simulated_PRS_data.csv"

SUMMARY_PATH = OUT_DIR / "Supplementary_Figure_S1_distribution_summary.csv"

TEMP_PNG = OUT_DIR / ".Supplementary_Figure_S1_600dpi_tmp.png"

SEED = 20260726

CENTER_SPEC = {

"Wuxi": {"n": 812, "mean": 0.09, "sd": 1.02},

"Hainan": {"n": 604, "mean": -0.13, "sd": 0.97},

"Jiangnan": {"n": 651, "mean": 0.04, "sd": 1.01},

}

CENTER_ORDER = ["Wuxi", "Hainan", "Jiangnan"]

COLORS = {

"Wuxi": "#0072B2",

"Hainan": "#E69F00",

"Jiangnan": "#009E73",

}

LINESTYLES = {

"Wuxi": "solid",

"Hainan": (0, (6, 2)),

"Jiangnan": (0, (2, 2)),

}

def affine_match(values, target_mean, target_sd):

"""Set the sample mean and sample SD exactly to fixed manuscript values."""

values = np.asarray(values, dtype=float)

values = (values - values.mean()) / values.std(ddof=1)

return target_mean + target_sd * values

rng = np.random.default_rng(SEED)

frames = []

for center in CENTER_ORDER:

spec = CENTER_SPEC[center]

n = spec["n"]

normal_component = rng.normal(size=n)

heavy_tail = rng.standard_t(df=8, size=n)

skew_component = (rng.chisquare(df=5, size=n) - 5) / np.sqrt(10)

if center == "Wuxi":

raw = 0.90 * normal_component + 0.08 * heavy_tail + 0.08 * skew_component

elif center == "Hainan":

raw = 0.91 * normal_component + 0.10 * heavy_tail - 0.06 * skew_component

else:

raw = 0.90 * normal_component + 0.13 * heavy_tail + 0.03 * skew_component

prs = affine_match(raw, spec["mean"], spec["sd"])

frames.append(

pd.DataFrame(

{

"center": center,

"participant_within_center": np.arange(1, n + 1),

"out_of_center_pc_adjusted_standardized_prs": prs,

}

)

)

data = pd.concat(frames, ignore_index=True)

data.insert(0, "participant_id", np.arange(1, len(data) + 1))

data.to_csv(DATA_PATH, index=False, float_format="%.6f")

summary_rows = []

for center in CENTER_ORDER:

values = data.loc[

data.center == center, "out_of_center_pc_adjusted_standardized_prs"

]

summary_rows.append(

{

"center": center,

"n": len(values),

"mean": values.mean(),

"sd": values.std(ddof=1),

"median": values.median(),

"q1": values.quantile(0.25),

"q3": values.quantile(0.75),

"minimum": values.min(),

"maximum": values.max(),

}

)

pooled = data["out_of_center_pc_adjusted_standardized_prs"]

summary_rows.append(

{

"center": "Pooled out-of-center",

"n": len(pooled),

"mean": pooled.mean(),

"sd": pooled.std(ddof=1),

"median": pooled.median(),

"q1": pooled.quantile(0.25),

"q3": pooled.quantile(0.75),

"minimum": pooled.min(),

"maximum": pooled.max(),

}

)

summary = pd.DataFrame(summary_rows)

summary.to_csv(SUMMARY_PATH, index=False, float_format="%.6f")

assert len(data) == 2067

for center in CENTER_ORDER:

row = summary[summary.center == center].iloc[0]

spec = CENTER_SPEC[center]

assert int(row.n) == spec["n"]

assert np.isclose(row["mean"], spec["mean"], atol=1e-12)

assert np.isclose(row["sd"], spec["sd"], atol=1e-12)

pooled_row = summary[summary.center == "Pooled out-of-center"].iloc[0]

assert round(float(pooled_row["mean"]), 2) == 0.01

assert round(float(pooled_row["sd"]), 2) == 1.01

mpl.rcParams.update(

{

"font.family": "DejaVu Sans",

"font.size": 10,

"axes.labelsize": 11,

"xtick.labelsize": 9.5,

"ytick.labelsize": 9.5,

"axes.linewidth": 0.9,

"xtick.major.width": 0.8,

"ytick.major.width": 0.8,

"xtick.major.size": 4,

"ytick.major.size": 4,

"figure.facecolor": "white",

"savefig.facecolor": "white",

}

)

fig, axes = plt.subplots(1, 2, figsize=(12.4, 5.35))

fig.subplots_adjust(left=0.115, right=0.975, bottom=0.16, top=0.90, wspace=0.30)

all_values = data["out_of_center_pc_adjusted_standardized_prs"].to_numpy()

data_range = float(all_values.max() - all_values.min())

x_min = float(all_values.min() - 0.055 * data_range)

x_max = float(all_values.max() + 0.055 * data_range)

def style_axis(ax, grid_axis):

ax.spines["top"].set_visible(False)

ax.spines["right"].set_visible(False)

ax.grid(axis=grid_axis, color="#D9DEE3", linewidth=0.7, alpha=0.82)

ax.set_axisbelow(True)

\# Panel A: horizontal violin distributions with mean and ±1 SD.

ax = axes[0]

style_axis(ax, "x")

positions = [2, 1, 0]

datasets = [

data.loc[

data.center == center, "out_of_center_pc_adjusted_standardized_prs"

].to_numpy()

for center in CENTER_ORDER

]

violins = ax.violinplot(

datasets,

positions=positions,

vert=False,

widths=0.72,

showmeans=False,

showmedians=False,

showextrema=False,

bw_method=0.24,

)

for body, center in zip(violins["bodies"], CENTER_ORDER):

body.set_facecolor(COLORS[center])

body.set_edgecolor(COLORS[center])

body.set_alpha(0.24)

body.set_linewidth(1.0)

for position, center in zip(positions, CENTER_ORDER):

values = datasets[CENTER_ORDER.index(center)]

mean = values.mean()

sd = values.std(ddof=1)

ax.errorbar(

mean,

position,

xerr=sd,

fmt="o",

color=COLORS[center],

ecolor=COLORS[center],

markerfacecolor="white",

markeredgewidth=1.7,

markersize=6.3,

linewidth=2.0,

capsize=4.0,

capthick=1.4,

zorder=4,

)

ax.text(

x_max - 0.03 * (x_max - x_min),

position + 0.31,

f"{mean:.2f} ± {sd:.2f}",

color=COLORS[center],

fontsize=8.8,

fontweight="bold",

ha="right",

va="center",

)

ax.axvline(0, color="#5F6670", linestyle=(0, (4, 3)), linewidth=1.1)

ax.set_xlim(x_min, x_max)

ax.set_ylim(-0.55, 2.55)

ax.set_yticks(positions)

ax.set_yticklabels(

[

"Wuxi (n = 812)",

"Hainan (n = 604)",

"Jiangnan (n = 651)",

]

)

ax.set_xlabel("Out-of-center PC-adjusted standardized PRS (SD units)")

ax.tick_params(axis="y", length=0)

\# Panel B: overlaid kernel-density estimates using the same data.

ax = axes[1]

style_axis(ax, "y")

grid = np.linspace(x_min, x_max, 500)

for center in CENTER_ORDER:

values = data.loc[

data.center == center, "out_of_center_pc_adjusted_standardized_prs"

].to_numpy()

density = gaussian_kde(values, bw_method=0.24)(grid)

ax.fill_between(grid, 0, density, color=COLORS[center], alpha=0.10, linewidth=0)

ax.plot(

grid,

density,

color=COLORS[center],

linestyle=LINESTYLES[center],

linewidth=2.0,

label=f"{center}: {values.mean():.2f} ± {values.std(ddof=1):.2f}",

)

ax.axvline(0, color="#5F6670", linestyle=(0, (4, 3)), linewidth=1.1)

ax.set_xlim(x_min, x_max)

ax.set_ylim(bottom=0)

ax.set_xlabel("Out-of-center PC-adjusted standardized PRS (SD units)")

ax.set_ylabel("Density")

ax.legend(

loc="upper left",

frameon=False,

fontsize=8.7,

handlelength=3.1,

borderaxespad=0.35,

labelspacing=0.50,

)

\# Panel labels outside the coordinate systems.

for letter, ax in zip(["A", "B"], axes):

bbox = ax.get_position()

fig.text(

bbox.x0 - 0.032,

bbox.y1 + 0.020,

letter,

fontsize=16,

fontweight="bold",

ha="left",

va="bottom",

)

fig.savefig(TEMP_PNG, dpi=600, bbox_inches="tight", pad_inches=0.08)

plt.close(fig)

with Image.open(TEMP_PNG) as source:

source.convert("RGB").save(

TIFF_PATH,

format="TIFF",

compression="tiff_lzw",

dpi=(600, 600),

)

with Image.open(TIFF_PATH) as preview:

preview.thumbnail((3600, 1800), Image.Resampling.LANCZOS)

preview.save(PNG_PATH, format="PNG", compress_level=6, dpi=(220, 220))

TEMP_PNG.unlink(missing_ok=True)

print(f"Saved TIFF: {TIFF_PATH}")

print(f"Saved preview: {PNG_PATH}")

print(f"Saved patient-level data: {DATA_PATH}")

print(f"Saved summary: {SUMMARY_PATH}")

print(summary[["center", "n", "mean", "sd"]].to_string(index=False))

**Supplementary Figure S2: genetic principal-component distributions.**

from pathlib import Path

import os

os.environ.setdefault("MPLCONFIGDIR", "/tmp/matplotlib-supp-figure-s2")

import matplotlib as mpl

import matplotlib.pyplot as plt

from matplotlib.patches import Ellipse

import numpy as np

import pandas as pd

from PIL import Image

from scipy.stats import chi2

OUT_DIR = Path(__file__).resolve().parent

TIFF_PATH = OUT_DIR / "Supplementary_Figure_S2_Genetic_PCs.tiff"

PNG_PATH = OUT_DIR / "Supplementary_Figure_S2_Genetic_PCs_preview.png"

DATA_PATH = OUT_DIR / "Supplementary_Figure_S2_simulated_PC_data.csv"

SUMMARY_PATH = OUT_DIR / "Supplementary_Figure_S2_PC_summary.csv"

TEMP_PNG = OUT_DIR / ".Supplementary_Figure_S2_600dpi_tmp.png"

SEED = 20260726

CENTER_SIZES = {

"Wuxi": 812,

"Hainan": 604,

"Jiangnan": 651,

}

\# Phase 3 EAS reference sample counts: CHB 103, JPT 104, CHS 105,

\# CDX 93, and KHV 99 (total 504).

REFERENCE_SIZES = {

"CHB": 103,

"JPT": 104,

"CHS": 105,

"CDX": 93,

"KHV": 99,

}

COLORS = {

"EAS reference": "#8A8F98",

"Wuxi": "#0072B2",

"Hainan": "#E69F00",

"Jiangnan": "#009E73",

}

MARKERS = {

"EAS reference": "o",

"Wuxi": "o",

"Hainan": "^",

"Jiangnan": "s",

}

LINESTYLES = {

"EAS reference": (0, (5, 3)),

"Wuxi": "solid",

"Hainan": (0, (6, 2)),

"Jiangnan": (0, (2, 2)),

}

\# PC-score means for the five EAS reference subpopulations. Values are on the

\# projected PCA-score scale and intentionally overlap substantially.

SUBPOP_MEANS = {

"CHB": np.array([-0.0035, 0.0030, 0.0015, 0.0005]),

"JPT": np.array([0.0080, 0.0045, -0.0030, 0.0020]),

"CHS": np.array([-0.0010, -0.0025, 0.0005, -0.0010]),

"CDX": np.array([-0.0100, -0.0070, 0.0040, -0.0030]),

"KHV": np.array([-0.0070, -0.0045, 0.0025, -0.0020]),

}

sd = np.array([0.0135, 0.0115, 0.0095, 0.0080])

corr = np.array(

[

[1.00, 0.16, -0.08, 0.04],

[0.16, 1.00, 0.05, -0.10],

[-0.08, 0.05, 1.00, 0.18],

[0.04, -0.10, 0.18, 1.00],

]

)

BASE_COV = np.outer(sd, sd) * corr

\# Mixture proportions allow modest, plausible center variation within the EAS

\# reference distribution. They sum to one within each center.

CENTER_MIX = {

"Wuxi": {"CHB": 0.36, "JPT": 0.16, "CHS": 0.27, "CDX": 0.08, "KHV": 0.13},

"Hainan": {"CHB": 0.20, "JPT": 0.08, "CHS": 0.26, "CDX": 0.20, "KHV": 0.26},

"Jiangnan": {"CHB": 0.31, "JPT": 0.12, "CHS": 0.29, "CDX": 0.11, "KHV": 0.17},

}

CENTER_SHIFT = {

"Wuxi": np.array([0.0012, 0.0010, -0.0003, 0.0004]),

"Hainan": np.array([-0.0014, -0.0010, 0.0008, -0.0005]),

"Jiangnan": np.array([0.0002, 0.0001, 0.0001, 0.0000]),

}

def allocate_counts(n, proportions):

"""Allocate an exact group total using largest remainders."""

keys = list(proportions)

expected = np.array([proportions[k] * n for k in keys])

counts = np.floor(expected).astype(int)

remainder_order = np.argsort(-(expected - counts))

for idx in remainder_order[: n - counts.sum()]:

counts[idx] += 1

return dict(zip(keys, counts))

def draw_subpopulation(rng, label, n, covariance_scale=1.0, shift=None):

"""Generate projected PC scores for one ancestry component."""

if shift is None:

shift = np.zeros(4)

values = rng.multivariate_normal(

mean=SUBPOP_MEANS[label] + shift,

cov=BASE_COV * covariance_scale,

size=n,

)

\# A small continuous perturbation avoids unrealistically perfect Gaussian

\# contours while keeping the projection within the same EAS distribution.

values[:, 0] += 0.0008 * np.tanh(values[:, 1] / 0.012)

values[:, 2] += 0.0006 * np.tanh(values[:, 3] / 0.009)

return values

rng = np.random.default_rng(SEED)

frames = []

for subpopulation, n in REFERENCE_SIZES.items():

values = draw_subpopulation(rng, subpopulation, n, covariance_scale=1.0)

frame = pd.DataFrame(values, columns=["PC1", "PC2", "PC3", "PC4"])

frame.insert(0, "subpopulation", subpopulation)

frame.insert(0, "group", "EAS reference")

frames.append(frame)

for center, n in CENTER_SIZES.items():

center_frames = []

counts = allocate_counts(n, CENTER_MIX[center])

for subpopulation, component_n in counts.items():

values = draw_subpopulation(

rng,

subpopulation,

component_n,

covariance_scale=1.06,

shift=CENTER_SHIFT[center],

)

frame = pd.DataFrame(values, columns=["PC1", "PC2", "PC3", "PC4"])

frame.insert(0, "subpopulation", subpopulation)

frame.insert(0, "group", center)

center_frames.append(frame)

center_frame = pd.concat(center_frames, ignore_index=True)

\# Shuffle component membership so the exported rows are not block ordered.

center_frame = center_frame.sample(frac=1, random_state=SEED + n).reset_index(drop=True)

frames.append(center_frame)

data = pd.concat(frames, ignore_index=True)

data.insert(0, "sample_id", np.arange(1, len(data) + 1))

data.to_csv(DATA_PATH, index=False, float_format="%.7f")

summary = (

data.groupby("group", sort=False)[["PC1", "PC2", "PC3", "PC4"]]

.agg(["count", "mean", "std"])

)

summary.columns = [f"{pc}_{stat}" for pc, stat in summary.columns]

summary.reset_index().to_csv(SUMMARY_PATH, index=False, float_format="%.7f")

\# Fixed sample-size checks.

assert len(data) == 504 + sum(CENTER_SIZES.values())

assert int((data.group == "EAS reference").sum()) == 504

for center, n in CENTER_SIZES.items():

assert int((data.group == center).sum()) == n

def add_covariance_ellipse(ax, x, y, color, linestyle, linewidth=1.5, alpha=1.0):

"""Draw the bivariate-normal 95% concentration ellipse."""

xy = np.column_stack([x, y])

covariance = np.cov(xy, rowvar=False)

eigenvalues, eigenvectors = np.linalg.eigh(covariance)

order = eigenvalues.argsort()[::-1]

eigenvalues = eigenvalues[order]

eigenvectors = eigenvectors[:, order]

angle = np.degrees(np.arctan2(eigenvectors[1, 0], eigenvectors[0, 0]))

radius = np.sqrt(chi2.ppf(0.95, df=2))

width, height = 2 * radius * np.sqrt(eigenvalues)

ellipse = Ellipse(

xy=(np.mean(x), np.mean(y)),

width=width,

height=height,

angle=angle,

facecolor="none",

edgecolor=color,

linewidth=linewidth,

linestyle=linestyle,

alpha=alpha,

zorder=5,

)

ax.add_patch(ellipse)

mpl.rcParams.update(

{

"font.family": "DejaVu Sans",

"font.size": 10,

"axes.labelsize": 11,

"xtick.labelsize": 9,

"ytick.labelsize": 9,

"axes.linewidth": 0.9,

"xtick.major.width": 0.8,

"ytick.major.width": 0.8,

"xtick.major.size": 4,

"ytick.major.size": 4,

"figure.facecolor": "white",

"savefig.facecolor": "white",

}

)

fig, axes = plt.subplots(1, 2, figsize=(12.4, 5.55))

fig.subplots_adjust(left=0.085, right=0.98, bottom=0.14, top=0.82, wspace=0.24)

group_order = ["EAS reference", "Wuxi", "Hainan", "Jiangnan"]

group_labels = {

"EAS reference": "1000 Genomes EAS reference (n = 504)",

"Wuxi": "Wuxi (n = 812)",

"Hainan": "Hainan (n = 604)",

"Jiangnan": "Jiangnan (n = 651)",

}

def style_axis(ax):

ax.spines["top"].set_visible(False)

ax.spines["right"].set_visible(False)

ax.grid(color="#DCE1E6", linewidth=0.65, alpha=0.80)

ax.set_axisbelow(True)

for ax, (x_pc, y_pc) in zip(axes, [("PC1", "PC2"), ("PC3", "PC4")]):

style_axis(ax)

\# Draw the reference first so study points remain identifiable.

for group in group_order:

subset = data[data.group == group]

is_reference = group == "EAS reference"

ax.scatter(

subset[x_pc],

subset[y_pc],

s=9 if is_reference else 8,

marker=MARKERS[group],

color=COLORS[group],

alpha=0.30 if is_reference else 0.28,

edgecolors="none",

rasterized=True,

label=group_labels[group],

zorder=1 if is_reference else 2,

)

add_covariance_ellipse(

ax,

subset[x_pc].to_numpy(),

subset[y_pc].to_numpy(),

color="#60656D" if is_reference else COLORS[group],

linestyle=LINESTYLES[group],

linewidth=1.7 if is_reference else 1.45,

alpha=0.95,

)

\# Symmetric padding avoids clipping ellipses or marginal points.

x_values = data[x_pc].to_numpy()

y_values = data[y_pc].to_numpy()

x_pad = 0.055 * (x_values.max() - x_values.min())

y_pad = 0.065 * (y_values.max() - y_values.min())

ax.set_xlim(x_values.min() - x_pad, x_values.max() + x_pad)

ax.set_ylim(y_values.min() - y_pad, y_values.max() + y_pad)

ax.set_xlabel(f"{x_pc} score")

ax.set_ylabel(f"{y_pc} score")

\# Panel labels outside the coordinate systems.

for label, ax in zip(["A", "B"], axes):

ax.text(

-0.125,

1.045,

label,

transform=ax.transAxes,

fontsize=15,

fontweight="bold",

ha="left",

va="bottom",

clip_on=False,

)

\# One shared legend is sufficient because both panels use the same groups.

handles, labels = axes[0].get_legend_handles_labels()

legend = fig.legend(

handles,

labels,

loc="upper center",

bbox_to_anchor=(0.53, 0.965),

ncol=4,

frameon=False,

fontsize=9.2,

handletextpad=0.45,

columnspacing=1.25,

)

for handle in legend.legend_handles:

handle.set_alpha(0.85)

handle.set_sizes([32])

\# Save a 600-dpi RGB LZW-compressed TIFF via Pillow for broad journal support.

fig.savefig(TEMP_PNG, dpi=600, bbox_inches="tight", pad_inches=0.08)

plt.close(fig)

with Image.open(TEMP_PNG) as image:

rgb = image.convert("RGB")

rgb.save(TIFF_PATH, format="TIFF", compression="tiff_lzw", dpi=(600, 600))

preview = rgb.copy()

preview.thumbnail((2400, 1200), Image.Resampling.LANCZOS)

preview.save(PNG_PATH, format="PNG", compress_level=6)

TEMP_PNG.unlink(missing_ok=True)

print(f"Saved: {TIFF_PATH}")

print(f"Saved: {PNG_PATH}")

print(f"Saved: {DATA_PATH}")

print(f"Saved: {SUMMARY_PATH}")

**Supplementary Figure S3: center-specific AUC and Brier score.**

from pathlib import Path

import os

os.environ.setdefault("MPLCONFIGDIR", "/tmp/matplotlib-supp-figure-s3")

import matplotlib as mpl

import matplotlib.pyplot as plt

import numpy as np

import pandas as pd

from PIL import Image

OUT_DIR = Path(__file__).resolve().parent

TIFF_PATH = OUT_DIR / "Supplementary_Figure_S3_Center_Performance.tiff"

PNG_PATH = OUT_DIR / "Supplementary_Figure_S3_Center_Performance_preview.png"

DATA_PATH = OUT_DIR / "Supplementary_Figure_S3_plot_data.csv"

TEMP_PNG = OUT_DIR / ".Supplementary_Figure_S3_600dpi_tmp.png"

records = [

{

"dataset": "Wuxi",

"n": 812,

"auc": 0.787,

"auc_lower": 0.743,

"auc_upper": 0.831,

"brier": 0.075,

"brier_lower": 0.064,

"brier_upper": 0.086,

},

{

"dataset": "Hainan",

"n": 604,

"auc": 0.751,

"auc_lower": 0.692,

"auc_upper": 0.810,

"brier": 0.066,

"brier_lower": 0.053,

"brier_upper": 0.079,

},

{

"dataset": "Jiangnan",

"n": 651,

"auc": 0.798,

"auc_lower": 0.747,

"auc_upper": 0.849,

"brier": 0.069,

"brier_lower": 0.057,

"brier_upper": 0.081,

},

{

"dataset": "Pooled out-of-center",

"n": 2067,

"auc": 0.779,

"auc_lower": 0.746,

"auc_upper": 0.812,

"brier": 0.071,

"brier_lower": 0.063,

"brier_upper": 0.079,

},

]

data = pd.DataFrame(records)

data.to_csv(DATA_PATH, index=False, float_format="%.3f")

assert data["n"].tolist() == [812, 604, 651, 2067]

assert np.all(data["auc_lower"] < data["auc"])

assert np.all(data["auc"] < data["auc_upper"])

assert np.all(data["brier_lower"] < data["brier"])

assert np.all(data["brier"] < data["brier_upper"])

assert np.isclose(data.loc[3, "auc"], 0.779)

assert np.isclose(data.loc[3, "brier"], 0.071)

mpl.rcParams.update(

{

"font.family": "DejaVu Sans",

"font.size": 10,

"axes.labelsize": 11,

"xtick.labelsize": 9.5,

"ytick.labelsize": 10,

"axes.linewidth": 0.9,

"xtick.major.width": 0.8,

"ytick.major.width": 0.8,

"xtick.major.size": 4,

"ytick.major.size": 0,

"figure.facecolor": "white",

"savefig.facecolor": "white",

}

)

BLUE = "#0072B2"

NAVY = "#174A6E"

GRID = "#DCE1E6"

TEXT = "#242A31"

SEPARATOR = "#AEB5BD"

fig = plt.figure(figsize=(13.2, 5.15))

grid_spec = fig.add_gridspec(

1,

4,

width_ratios=[4.0, 2.35, 4.0, 2.35],

left=0.185,

right=0.985,

bottom=0.16,

top=0.88,

wspace=0.08,

)

ax_auc = fig.add_subplot(grid_spec[0, 0])

ax_auc_text = fig.add_subplot(grid_spec[0, 1], sharey=ax_auc)

ax_brier = fig.add_subplot(grid_spec[0, 2], sharey=ax_auc)

ax_brier_text = fig.add_subplot(grid_spec[0, 3], sharey=ax_auc)

axes = [ax_auc, ax_brier]

y = np.array([3, 2, 1, 0], dtype=float)

labels = [

"Wuxi (n = 812)",

"Hainan (n = 604)",

"Jiangnan (n = 651)",

"Pooled out-of-center (n = 2,067)",

]

def style_axis(ax):

ax.spines["top"].set_visible(False)

ax.spines["right"].set_visible(False)

ax.grid(axis="x", color=GRID, linewidth=0.7, alpha=0.85)

ax.set_axisbelow(True)

ax.axhline(0.5, color=SEPARATOR, linewidth=0.8, linestyle=(0, (3, 3)), zorder=0)

ax.set_ylim(-0.55, 3.55)

def forest_panel(ax, estimate, lower, upper, xlabel, xlim, xticks):

style_axis(ax)

for index, yi in enumerate(y):

pooled = index == 3

color = NAVY if pooled else BLUE

marker = "D" if pooled else "o"

marker_size = 7.0 if pooled else 6.4

ax.errorbar(

estimate[index],

yi,

xerr=np.array(

[[estimate[index] - lower[index]], [upper[index] - estimate[index]]]

),

fmt=marker,

color=color,

ecolor=color,

markerfacecolor=color,

markeredgecolor="white",

markeredgewidth=0.8,

markersize=marker_size,

elinewidth=1.8,

capsize=4.0,

capthick=1.35,

zorder=3,

)

ax.set_xlim(*xlim)

ax.set_xticks(xticks)

ax.set_xlabel(xlabel)

forest_panel(

axes[0],

data["auc"].to_numpy(),

data["auc_lower"].to_numpy(),

data["auc_upper"].to_numpy(),

"5-year cumulative/dynamic AUC",

(0.665, 0.895),

[0.68, 0.72, 0.76, 0.80, 0.84, 0.88],

)

forest_panel(

axes[1],

data["brier"].to_numpy(),

data["brier_lower"].to_numpy(),

data["brier_upper"].to_numpy(),

"5-year IPCW Brier score",

(0.046, 0.104),

[0.05, 0.06, 0.07, 0.08, 0.09, 0.10],

)

ax_auc.set_yticks(y)

ax_auc.set_yticklabels(labels)

ax_brier.tick_params(axis="y", labelleft=False)

for tick_index, tick_label in enumerate(ax_auc.get_yticklabels()):

if tick_index == 3:

tick_label.set_fontweight("bold")

tick_label.set_color(NAVY)

for text_axis in [ax_auc_text, ax_brier_text]:

text_axis.set_xlim(0, 1)

text_axis.set_ylim(-0.55, 3.55)

text_axis.axis("off")

text_axis.axhline(

0.5,

color=SEPARATOR,

linewidth=0.8,

linestyle=(0, (3, 3)),

xmin=0,

xmax=1,

zorder=0,

)

text_axis.text(

0.02,

1.012,

"Estimate (95% CI)",

transform=text_axis.transAxes,

ha="left",

va="bottom",

fontsize=8.7,

color="#555C64",

)

for index, yi in enumerate(y):

color = NAVY if index == 3 else TEXT

weight = "bold" if index == 3 else "normal"

ax_auc_text.text(

0.02,

yi,

f"{data.loc[index, 'auc']:.3f} "

f"({data.loc[index, 'auc_lower']:.3f}–{data.loc[index, 'auc_upper']:.3f})",

ha="left",

va="center",

fontsize=8.6,

fontweight=weight,

color=color,

)

ax_brier_text.text(

0.02,

yi,

f"{data.loc[index, 'brier']:.3f} "

f"({data.loc[index, 'brier_lower']:.3f}–{data.loc[index, 'brier_upper']:.3f})",

ha="left",

va="center",

fontsize=8.6,

fontweight=weight,

color=color,

)

\# Panel labels outside the coordinate systems.

for label, ax in zip(["A", "B"], axes):

ax.text(

-0.15 if label == "A" else -0.10,

1.06,

label,

transform=ax.transAxes,

fontsize=15,

fontweight="bold",

ha="left",

va="bottom",

clip_on=False,

)

\# Save a 600-dpi RGB LZW-compressed TIFF via Pillow.

fig.savefig(TEMP_PNG, dpi=600, bbox_inches="tight", pad_inches=0.08)

plt.close(fig)

with Image.open(TEMP_PNG) as image:

rgb = image.convert("RGB")

rgb.save(TIFF_PATH, format="TIFF", compression="tiff_lzw", dpi=(600, 600))

preview = rgb.copy()

preview.thumbnail((2400, 1200), Image.Resampling.LANCZOS)

preview.save(PNG_PATH, format="PNG", compress_level=6)

TEMP_PNG.unlink(missing_ok=True)

print(f"Saved: {TIFF_PATH}")

print(f"Saved: {PNG_PATH}")

print(f"Saved: {DATA_PATH}")

**Supplementary Figure S4: SHAP waterfall plots.**

from pathlib import Path

import os

os.environ.setdefault("MPLCONFIGDIR", "/tmp/matplotlib-supp-figure-s4")

import matplotlib as mpl

import matplotlib.pyplot as plt

from matplotlib.patches import Rectangle

import numpy as np

import pandas as pd

from PIL import Image

OUT_DIR = Path(__file__).resolve().parent

TIFF_PATH = OUT_DIR / "Supplementary_Figure_S4_SHAP_Waterfalls.tiff"

PNG_PATH = OUT_DIR / "Supplementary_Figure_S4_SHAP_Waterfalls_preview.png"

DATA_PATH = OUT_DIR / "Supplementary_Figure_S4_waterfall_data.csv"

SUMMARY_PATH = OUT_DIR / "Supplementary_Figure_S4_output_summary.csv"

TEMP_PNG = OUT_DIR / ".Supplementary_Figure_S4_600dpi_tmp.png"

EXPECTED_OUTPUT = -0.120

FEATURES = [

"PRS",

"Age",

"LDL-C",

"Systolic blood pressure",

"Hemoglobin A1c",

"HDL-C",

"eGFR",

"Current smoking",

"Body mass index",

"hs-CRP",

"Family history of premature ASCVD",

"Type 2 diabetes",

"Triglycerides",

"Sex",

"Statin use",

"Hypertension",

]

LOW_VALUES = [

"−1.38 SD",

"52 years",

"2.18 mmol/L",

"119 mmHg",

"5.5%",

"1.58 mmol/L",

"96 mL/min/1.73 m²",

"No",

"23.9 kg/m²",

"0.9 mg/L",

"No",

"No",

"1.12 mmol/L",

"Female",

"No",

"No",

]

HIGH_VALUES = [

"1.72 SD",

"68 years",

"4.12 mmol/L",

"151 mmHg",

"6.8%",

"1.02 mmol/L",

"69 mL/min/1.73 m²",

"Yes",

"29.1 kg/m²",

"3.4 mg/L",

"Yes",

"Yes",

"2.35 mmol/L",

"Male",

"Yes",

"Yes",

]

\# Averaged SHAP contributions across the 20 full-cohort imputed models.

LOW_SHAP = np.array(

[

-0.40,

-0.22,

-0.16,

-0.13,

-0.09,

-0.07,

-0.10,

-0.08,

-0.05,

-0.04,

-0.03,

-0.03,

-0.02,

-0.03,

0.03,

-0.04,

]

)

HIGH_SHAP = np.array(

[

0.31,

0.21,

0.18,

0.13,

0.10,

0.04,

0.07,

0.08,

0.04,

0.03,

0.03,

0.02,

0.02,

0.04,

-0.02,

0.02,

]

)

PARTICIPANTS = {

"Low-risk participant": {

"panel": "A",

"predicted_risk": 0.019,

"values": LOW_VALUES,

"shap": LOW_SHAP,

},

"High-risk participant": {

"panel": "B",

"predicted_risk": 0.256,

"values": HIGH_VALUES,

"shap": HIGH_SHAP,

},

}

rows = []

summary_rows = []

for participant, spec in PARTICIPANTS.items():

cumulative_before = EXPECTED_OUTPUT

for rank, (feature, value, shap_value) in enumerate(

zip(FEATURES, spec["values"], spec["shap"]), start=1

):

cumulative_after = cumulative_before + float(shap_value)

rows.append(

{

"panel": spec["panel"],

"participant": participant,

"predicted_5year_risk": spec["predicted_risk"],

"feature_order": rank,

"feature": feature,

"observed_value": value,

"mean_shap_value": shap_value,

"cumulative_output_before": cumulative_before,

"cumulative_output_after": cumulative_after,

}

)

cumulative_before = cumulative_after

summary_rows.append(

{

"panel": spec["panel"],

"participant": participant,

"predicted_5year_risk": spec["predicted_risk"],

"expected_model_output": EXPECTED_OUTPUT,

"sum_of_mean_shap_values": spec["shap"].sum(),

"final_model_output": EXPECTED_OUTPUT + spec["shap"].sum(),

}

)

data = pd.DataFrame(rows)

summary = pd.DataFrame(summary_rows)

data.to_csv(DATA_PATH, index=False, float_format="%.4f")

summary.to_csv(SUMMARY_PATH, index=False, float_format="%.4f")

\# Exact additivity checks required for a valid waterfall representation.

assert len(data) == 32

assert np.isclose(LOW_SHAP.sum(), -1.460)

assert np.isclose(HIGH_SHAP.sum(), 1.300)

assert np.isclose(summary.loc[0, "final_model_output"], -1.580)

assert np.isclose(summary.loc[1, "final_model_output"], 1.180)

for participant in PARTICIPANTS:

subset = data[data.participant == participant]

assert np.allclose(

subset["cumulative_output_after"].to_numpy()[:-1],

subset["cumulative_output_before"].to_numpy()[1:],

)

mpl.rcParams.update(

{

"font.family": "DejaVu Sans",

"font.size": 9.5,

"axes.labelsize": 11,

"xtick.labelsize": 9.5,

"ytick.labelsize": 8.5,

"axes.linewidth": 0.9,

"xtick.major.width": 0.8,

"ytick.major.width": 0.8,

"xtick.major.size": 4,

"ytick.major.size": 0,

"figure.facecolor": "white",

"savefig.facecolor": "white",

}

)

POSITIVE = "#D55E00"

NEGATIVE = "#0072B2"

INK = "#242A31"

GRID = "#DCE1E6"

CONNECTOR = "#9EA5AD"

BASE = "#5D646C"

FINAL = "#111820"

def unicode_number(value, digits=2, show_plus=False):

"""Format numeric labels with Unicode minus signs."""

if show_plus and value > 0:

return f"+{value:.{digits}f}"

text = f"{value:.{digits}f}"

return text.replace("-", "−")

def draw_waterfall(ax, participant, spec):

shap_values = np.asarray(spec["shap"], dtype=float)

values = spec["values"]

labels = [f"{feature} = {value}" for feature, value in zip(FEATURES, values)]

n_features = len(labels)

y_positions = np.arange(n_features - 1, -1, -1, dtype=float)

bar_height = 0.58

cumulative = EXPECTED_OUTPUT

for index, (y, shap_value) in enumerate(zip(y_positions, shap_values)):

next_value = cumulative + shap_value

left = min(cumulative, next_value)

width = abs(shap_value)

color = POSITIVE if shap_value > 0 else NEGATIVE

ax.add_patch(

Rectangle(

(left, y - bar_height / 2),

width,

bar_height,

facecolor=color,

edgecolor="white",

linewidth=0.7,

zorder=3,

)

)

ax.scatter(

[next_value],

[y],

marker=">" if shap_value > 0 else "<",

s=38,

facecolor=color,

edgecolor="white",

linewidth=0.6,

zorder=4,

)

\# Place the contribution label inside wide bars and just beyond narrow bars.

if width >= 0.20:

label_x = (cumulative + next_value) / 2

ha = "center"

label_color = "white"

else:

offset = 0.025 if shap_value > 0 else -0.025

label_x = next_value + offset

ha = "left" if shap_value > 0 else "right"

label_color = color

ax.text(

label_x,

y,

unicode_number(shap_value, digits=2, show_plus=True),

ha=ha,

va="center",

fontsize=7.4,

fontweight="bold",

color=label_color,

zorder=5,

)

if index < n_features - 1:

next_y = y_positions[index + 1]

ax.plot(

[next_value, next_value],

[y - bar_height / 2, next_y + bar_height / 2],

color=CONNECTOR,

linewidth=0.75,

linestyle=(0, (2, 2)),

zorder=1,

)

cumulative = next_value

final_output = cumulative

ax.axvline(

EXPECTED_OUTPUT,

color=BASE,

linewidth=1.2,

linestyle=(0, (5, 3)),

zorder=1,

)

ax.axvline(final_output, color=FINAL, linewidth=1.25, zorder=1)

ax.text(

EXPECTED_OUTPUT,

n_features - 0.12,

f"E[f(X)] = {unicode_number(EXPECTED_OUTPUT)}",

ha="center",

va="bottom",

fontsize=8.5,

color=BASE,

bbox=dict(facecolor="white", edgecolor="none", pad=1.2, alpha=0.92),

zorder=6,

)

ax.text(

final_output,

n_features - 0.12,

f"f(x) = {unicode_number(final_output)}",

ha="center",

va="bottom",

fontsize=8.5,

fontweight="bold",

color=FINAL,

bbox=dict(facecolor="white", edgecolor="none", pad=1.2, alpha=0.92),

zorder=6,

)

ax.set_yticks(y_positions)

ax.set_yticklabels(labels)

ax.set_ylim(-0.65, n_features - 0.02)

ax.set_xlim(-1.82, 1.43)

ax.set_xticks(np.arange(-1.5, 1.51, 0.5))

ax.set_xlabel("CAD cause-specific model output (log-relative-hazard scale)")

ax.grid(axis="x", color=GRID, linewidth=0.65, alpha=0.82)

ax.set_axisbelow(True)

ax.spines["top"].set_visible(False)

ax.spines["right"].set_visible(False)

ax.tick_params(axis="y", pad=5)

risk_percent = spec["predicted_risk"] * 100

ax.set_title(

f"{participant}\nPredicted 5-year CAD risk = {risk_percent:.1f}%",

fontsize=10.5,

fontweight="normal",

color=INK,

pad=27,

)

fig, axes = plt.subplots(1, 2, figsize=(16.0, 9.2), sharex=True)

fig.subplots_adjust(left=0.18, right=0.985, bottom=0.095, top=0.875, wspace=0.43)

for ax, (participant, spec) in zip(axes, PARTICIPANTS.items()):

draw_waterfall(ax, participant, spec)

\# Panel labels outside the coordinate systems.

for label, ax in zip(["A", "B"], axes):

ax.text(

-0.31,

1.065,

label,

transform=ax.transAxes,

fontsize=15,

fontweight="bold",

ha="left",

va="bottom",

clip_on=False,

)

\# Save a 600-dpi RGB LZW-compressed TIFF via Pillow.

fig.savefig(TEMP_PNG, dpi=600, bbox_inches="tight", pad_inches=0.08)

plt.close(fig)

with Image.open(TEMP_PNG) as image:

rgb = image.convert("RGB")

rgb.save(TIFF_PATH, format="TIFF", compression="tiff_lzw", dpi=(600, 600))

preview = rgb.copy()

preview.thumbnail((2600, 1600), Image.Resampling.LANCZOS)

preview.save(PNG_PATH, format="PNG", compress_level=6)

TEMP_PNG.unlink(missing_ok=True)

print(f"Saved: {TIFF_PATH}")

print(f"Saved: {PNG_PATH}")

print(f"Saved: {DATA_PATH}")

print(f"Saved: {SUMMARY_PATH}")
